# Design, click synthesis, and biological evaluation of novel uracil-1,2,3-triazole hybrids as dual EGFR/CDK1 inhibitors: antiproliferative activity, mechanistic insights, and *in silico* studies

**DOI:** 10.1039/d6ra09088e

**Published:** 2026-11-03

**Authors:** Asmaa H. Mohamed, Ashraf A. Aly, Elshimaa M. N. Abdelhafez, Aya A. Fouly, Alan B. Brown, Sageda A. Gedamy

**Affiliations:** a Chemistry Department, Faculty of Science, Minia University 61519 Minia Egypt asmaa.hamouda@mu.edu.eg ashrafaly63@yahoo.com ashraf.shehata@mu.edu.eg ayaattia2588@gmail.com sagedaali332@yahoo.com; b Department of Medicinal Chemistry, Faculty of Pharmacy, Minia University 61519 Minia Egypt shimaanaguib_80@mu.edu.eg; c Medicinal Chemistry Department, Faculty of Pharmacy, Minia National University New Minia 61768 Egypt; d Department of Chemistry and Chemical Engineering, Florida Institute of Technology Melbourne FL 32901 USA abrown@fit.edu

## Abstract

A novel series of uracil–1,2,3-triazole hybrids was designed, synthesized, and evaluated as dual EGFR/CDK1 inhibitors to target cancer cell proliferation and survival. Synthesis was initiated *via* the nucleophilic substitution of 2-chloro-*N*-(1,3-dimethyl-2,6-dioxo-pyrimidin-4-yl)acetamide (3) with sodium azide, yielding the key intermediate 4. Subsequent copper-catalyzed azide–alkyne cycloadditions (CuAAC) with various terminal alkynes and 1,3-dicarbonyls afforded the target triazole analogues 6a,b, 8a–c, and 10b,c. *In vitro* antiproliferative screening against a panel of four human cancer cell lines (MCF-7, HT29, PANC1, A549) and one normal cell line (Vero) identified compound 10a as a potential candidate. Compound 10a exhibited potent dual inhibitory activity, with IC_50_ values of 0.082 µM and 0.131 µM for EGFR and CDK1, respectively; it was about 2.4-fold less potent than erlotinib against EGFR but about 4.8-fold more potent than roscovitine against CDK1. Mechanistically, 10a induced significant G0/G1-phase cell-cycle arrest (74.89% *versus* 60.04% in control) and increased apoptotic cells (early plus late apoptosis, 13.55% *versus* 1.16% in control) in HT29 cells, accompanied by upregulation of Caspase-3 and BAX, with a rise in Bcl-2 whose significance remains to be established. Molecular docking predicted that 10a can occupy the ATP-binding pockets of both EGFR and CDK1, and normal-mode analysis (iMODS) suggested that the docked complexes are structurally plausible. Computational ADMET profiling, however, flags several liabilities for 10a, including predicted hepatotoxicity, low predicted oral absorption and a short predicted half-life. These findings highlight the uracil–triazole scaffold, specifically compound 10a, as a promising starting point for the development of targeted dual EGFR/CDK1 anticancer therapeutics.

## Abstract 2

A novel series of uracil–1,2,3-triazole hybrids was designed, synthesized, and evaluated as dual EGFR/CDK1 inhibitors to target cancer cell proliferation and survival.

## Graphical abstract

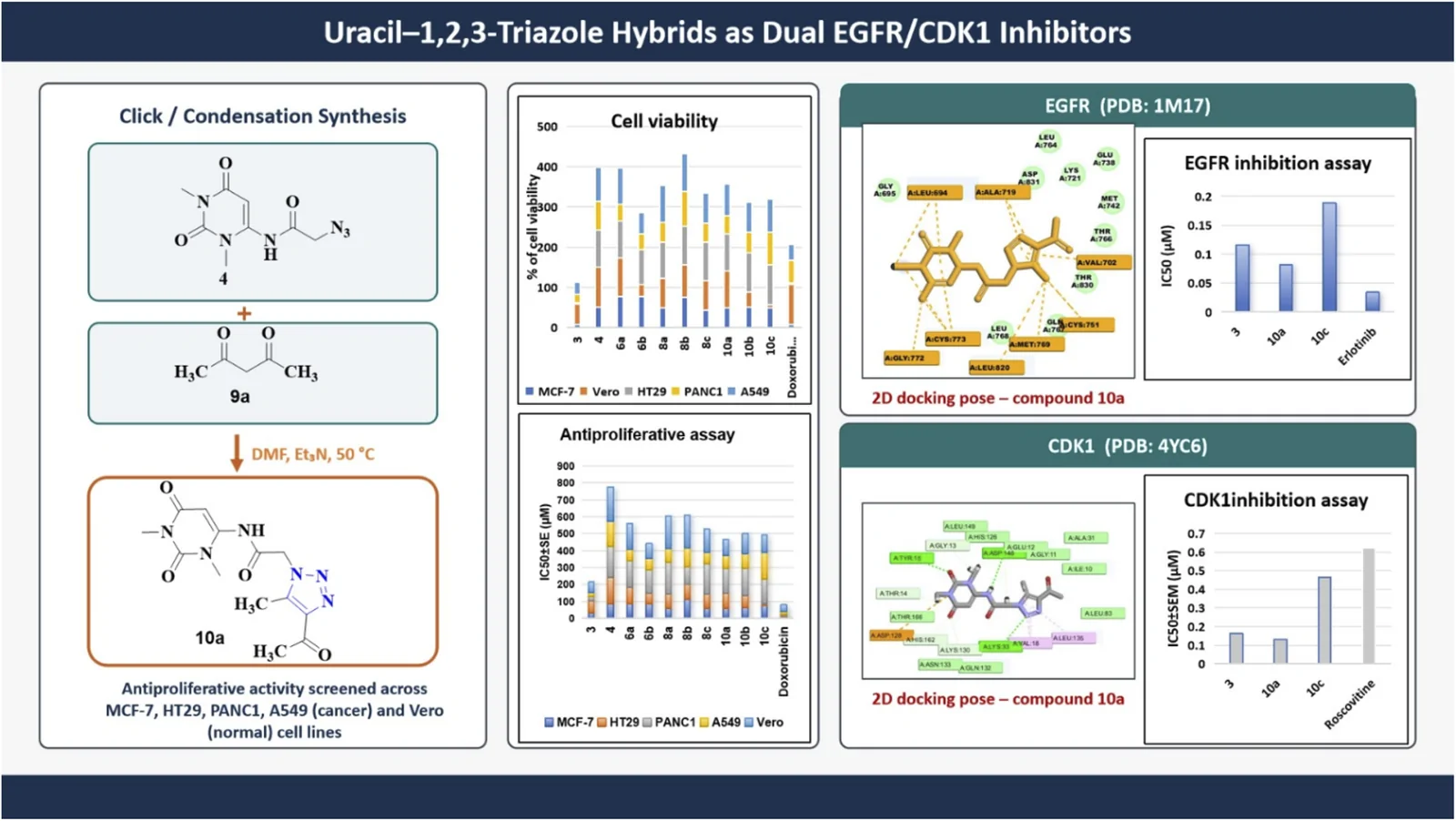

## 1. Introduction

Researchers have recently shown a great deal of interest in 1,2,3-triazoles and their derivatives. That is related to the utility of the former molecules in a variety of synthetic methods^1^ and have a wide range of possible pharmacological effects, including anticancer,^2^ antibacterial,^3^ antifungal,^4^ anti-inflammatory,^5^ anti-Alzheimer,^6^ antidiabetic,^7^ anti-oxidant^8^ activities.

Protein kinases, such as the epidermal growth factor receptor (EGFR) and the cyclin-dependent kinases (CDKs) including CDK1, play critical roles in cancer progression and drug resistance, and efforts have been made to regulate their activity in the treatment of cancer.^9^ The regulation of a number of important protein kinases, including the epidermal growth factor receptor (EGFR) and the cyclin-dependent kinases (CDKs) such as CDK1, is important for cancer progression and therapeutic resistance and has been targeted for cancer treatment. Single-target anti-cancer drugs can effectively treat the disease in the beginning, but their clinical efficacy is soon curtailed by drug resistance and cellular pathway compensation.^10–13^ The challenges have been overcome with a high likelihood of success by employing multi-targeting therapy.^14–16^ This will take the form of simultaneous inhibition of multiple kinase pathways by using hybrid molecular scaffolds to target EGFR and CDK networks simultaneously, successfully suppressing multiple, overlapping aspects of tumor growth and survival at once.^11,12,17,18^ In conclusion, multi-kinase inhibition provides a more powerful, long-lasting and effective anticancer effect than single-target inhibitors.^19^ Recent EGFR-inhibitor studies continue to highlight both the therapeutic value and the resistance liabilities of this target.^20^

The hybrid compound I (Fig. 1) was the most effective and highly antiproliferative hybrid with 50% growth inhibition concentration (GI_50_) of 25 nM. Furthermore, compound I was a very potent inhibitor of the EGFR enzyme with an IC_50_ of 73 nM and seemed to be a good candidate to efficiently induce apoptosis in cancer cells.^21^

**Fig. 1:**
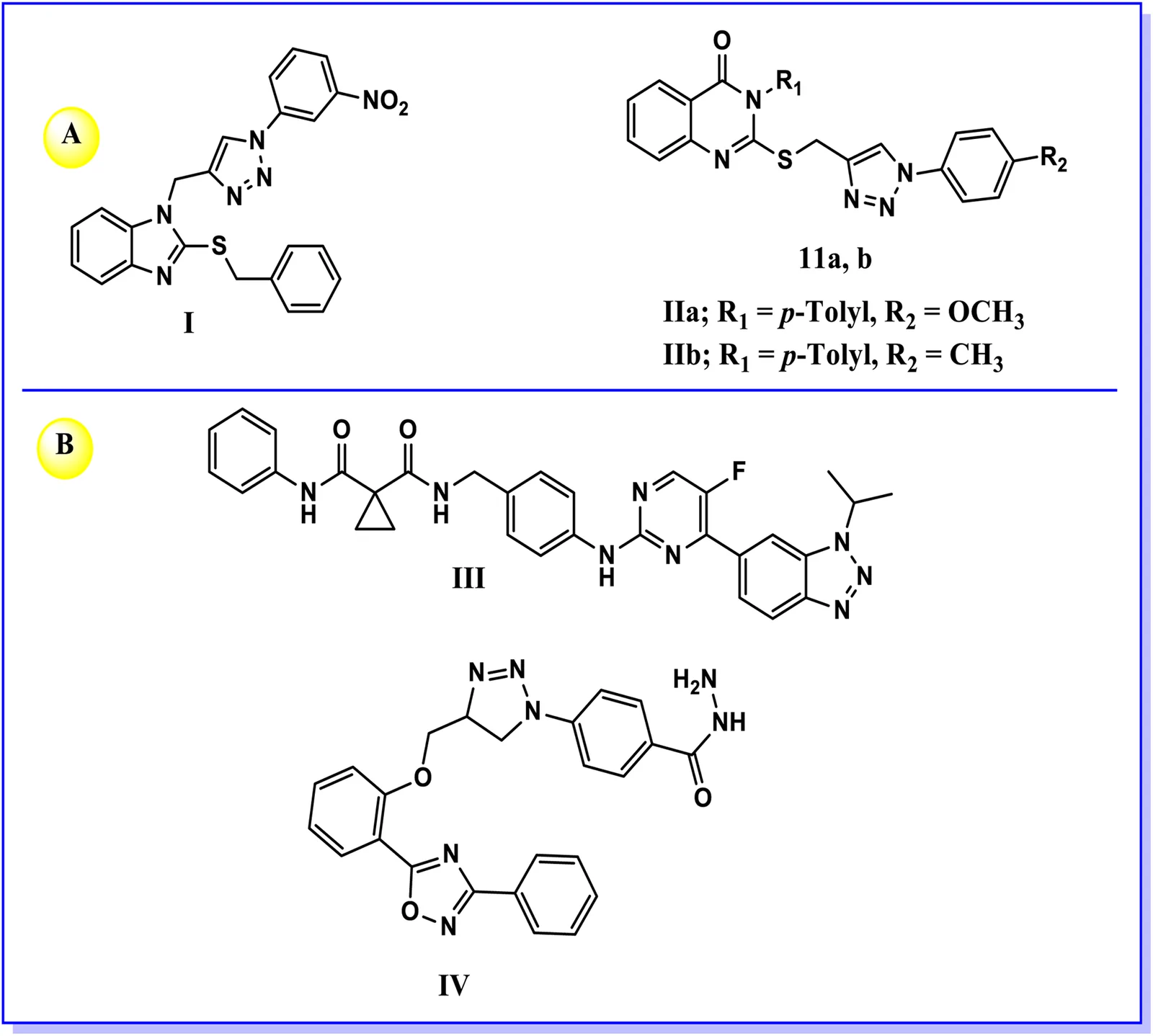
Reported 1,2,3-triazoles based anticancer drugs acting as (A) EGFR inhibitors and (B) CDK1 inhibitors.

It was found that compounds IIa and IIb (Fig. 1) were among the most potent synthesized agents; compound IIb particularly emerged as a potent antiproliferative agent, and a dual inhibitor of both EGFR and BRAF^V600E^. This compound has been identified as a potent inhibitor of the cell cycle of cancer cells at the G1 phase by biological analysis, which helps to inhibit the growth of tumor cells.^22^ In the study on pyrimidine-benzotriazole derivatives, III (Fig. 1) was proved to be the most promising and potent multi-kinase inhibitor. The molecular docking study revealed that the triazole group within III was crucial to form the critical hydrogen bond and favorable π–π interaction with the ATP binding pockets of CDKs. This allowed the compound to attain strong CDK inhibitory activities in the nanomolar range. In addition, *in vitro* and *in vivo* studies showed that III was highly inhibitory to the proliferation of cancer cells, colony formation and cell cycle progression. It was also able to significantly inhibit the growth of human tumor cells with a daily oral administration of 20 mg kg^−1^ in an SiHa xenograft mouse model without apparent toxicity.^23^ 1,2,4-Oxadiazole linked 1,2,3-triazole IV (Fig. 1) was reported as potent modulator of the EGFR/PI3K/mTOR signaling cascade.^19^ Compound IV proved to be one of the most active derivatives in the evaluation against A549 lung cancer and Caco-2 colon cancer cell lines. Compound IV was found to be the most effective inhibitor of PI3K, mTOR and EGFR and to cause strong upregulation of the tumor suppressor protein p53 in A549 cells. It also showed high levels of multi-kinase inhibition and high levels of activation of p53 in the Caco-2 cell line. In addition, molecular docking studies showed that compound IV has a higher binding affinity to the EGFR active site compared to the gefitinib reference drug, in agreement with its proposed mechanism of action.^19^ Compound V (Fig. 2) was found to be the most active derivative. It showed notable antiproliferative activity against breast, liver, and colon cancer cell lines with IC_50_ values of 5.1, 5.02 and 6.6 µM, respectively.^24^

**Fig. 2:**
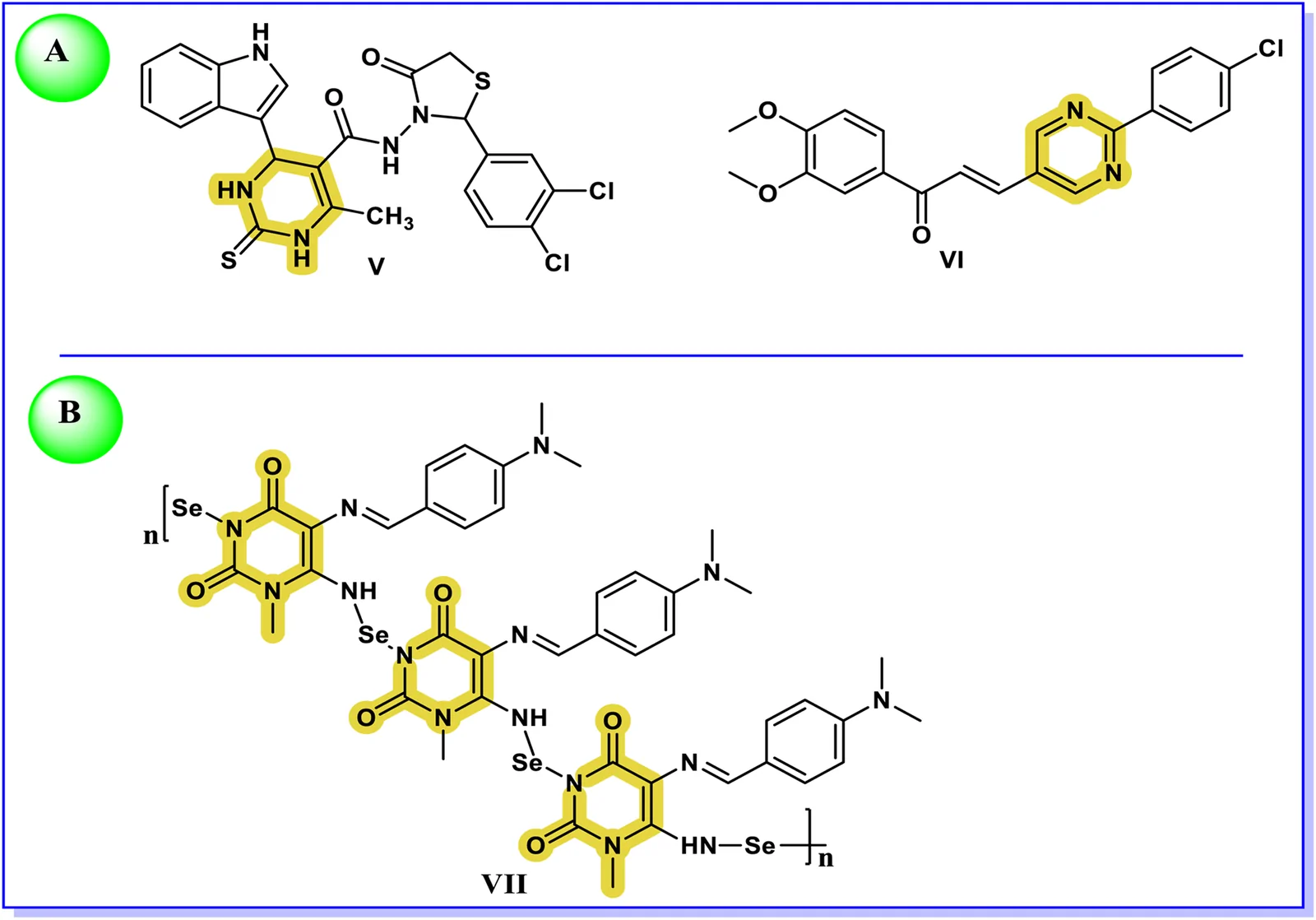
Reported pyrimidine/pyrimidine-2,4-dione based anticancer drugs acting as (A) EGFR inhibitors and (B) CDK1 inhibitors.

### 1.1. CDK1 inhibitors

Interestingly, compound V was the most active towards EGFR (IC_50_ = 0.25 µM) showing equipotency with the reference drug erlotinib. In addition, it exhibited as the highest *in vivo* anti-tumor activity in mice, demonstrated by molecular modeling of its docking inside the proper and stable active site of EGFR.^24^ Interest report indicated that compound VI (Fig. 2) exhibited high cytotoxicity in breast cancer (MCF-7) and lung cancer (A549) cell lines with IC_50_ value of 6.70 µM and 20.49 µM respectively, higher than the standard agent lapatinib.^25^ This was followed by the induced fit docking and molecular dynamics (MD) simulation of 300 ns which also demonstrated the high stability of B-4 with EGFR within a biomimicking environment, confirming that compound VI as a potent targeted EGFR inhibitor and apoptosis inducer.^24^ The selenium nanoform of pyrimidine derivative, compound VII (Fig. 2), proved to be the best dual inhibitor in this series. It showed an outstanding anti-proliferative effect in various cell lines (MCF-7, HepG-2, A549 cells) and was more than 93% effective than its non-nano equivalent. Highly active in inhibiting CDK1 with an IC_50_ of 0.47 µM, enzymatically. The pyrimidine core was found to be well docked in the ATP binding pocket of CDK1 through molecular docking. These results give a very pertinent structural context to consider the introduction of pyrimidine or new uracil scaffolds into dual-acting anticancer drugs.^26^

Aly *et al.*^27,28^ described the design, synthesis, and antiproliferative activity of uracil-based compounds and also 1,2,3-triazoles as possible dual inhibitors of EGFR and BRAF^V600E^.^29^

### 1.2. Rationale of designing target compounds

The design strategy used in this study is based on a binary-target inhibition approach, combining structural elements of two clinically validated kinase inhibitors roscovitine (a CDK1 inhibitor) and erlotinib (an EGFR inhibitor) in the same chemical scaffold that can simultaneously bind to both the CDK1 and EGFR active sites (Fig. 3). The purine core was simplified from roscovitine to maintain CDK1 recognition and its flexible hydroxymethyl-linker side chain was modified, maintaining key hydrogen-bonding interactions. The quinazoline-aniline pharmacophore necessary for binding with the EGFR was retained in erlotinib as a bioisosteric framework, and systematic substitution at the terminal aryl/heteroaryl group was performed to optimize the binding affinity and selectivity. The two pharmacophoric fragments were then merged together and elaborated to produce five different series (I–V; compounds 3, 4, 6a,b, 8a–c, and 10a–c; respectively) containing different groups that were structurally different to investigate different bioisosteric replacements and effects of substituents at the point of hybridization. The rational hybridization strategy aimed to result in dual acting antiproliferative compounds that were capable of blocking both cell-cycle progression by inhibiting CDK1 and proliferative signaling by inhibiting EGFR with the aim of achieving binary-function inhibition.

**Fig. 3:**
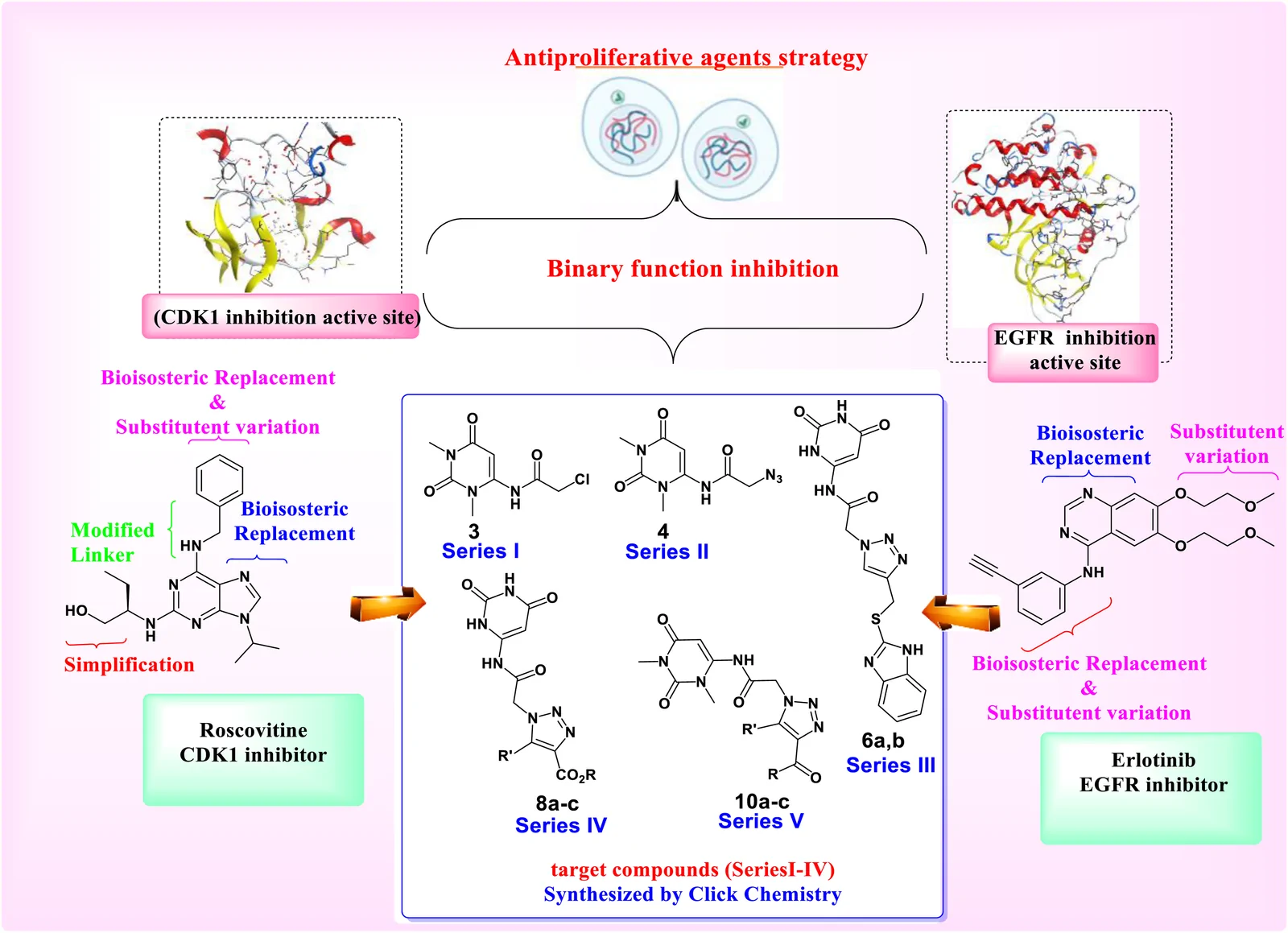
Rationale design of four series of 1,2,3-triazole/pyrimidine-1,2,4-dione based drugs (Series I–IV) for EGFR and CDK1 inhibition.

## 2. Results and discussion

### 2.1. Chemistry

This study outlines a two-step synthetic pathway for the preparation of 2-azido-*N*-(1,3-dimethyl-2,6-dioxo-1,2,3,6-tetrahydropyrimidin-4-yl)acetamide (4) starting from 6-amino-1,3-dimethyl-pyrimidine-2,4(1*H*,3*H*)-dione (1). The overall scheme demonstrates the functionalization of a pyrimidine core, a common scaffold in medicinal chemistry, to introduce an azide group, which is a versatile handle for further chemical modifications, particularly in biorthogonal reactions such as click chemistry.

The initial step involved the reaction of 6-amino-1,3-dimethylpyrimidine-2,4(1*H*,3*H*)-dione (1) with 2-chloroacetyl chloride (2). This reaction was carried out under mild conditions, specifically in dimethylformamide (DMF) using an ice bath for 2 h (Scheme 1). The product obtained from this reaction was 2-chloro-*N*-(1,3-dimethyl-2,6-dioxo-1,2,3,6-tetrahydropyrimidin-4-yl)acetamide (3). This transformation is a classic example of *N*-acylation, where the amino group of compound 1 acts as a nucleophile, attacking the carbonyl carbon of 2-chloroacetyl chloride (2), followed by the elimination of hydrogen chloride. The use of an ice bath suggests that the reaction is exothermic or that side reactions are minimized at lower temperatures, ensuring selective acylation of the primary amine. The resulting chloroacetamide derivative (3) serves as a crucial intermediate, possessing an electrophilic chlorine atom that can be readily displaced by various nucleophiles. In the second step, the chloroacetamide derivative (3) was subjected to a nucleophilic substitution reaction with sodium azide (NaN_3_). The reaction was performed in DMF at 90 °C for 2 h, yielding 2-azido-*N*-(1,3-dimethyl-2,6-dioxo-1,2,3,6-tetrahydro-pyrimidin-4-yl)acetamide (4) in 75% yield (Scheme 1). This reaction proceeds *via* an SN_2_ mechanism, where the azide anion displaces the chloride ion from the α-carbon of the acetamide moiety. The introduction of an azide group is a significant synthetic maneuver. Azide functionalities are well-known for their utility in click chemistry. This makes compound 4 a valuable building block for synthesizing more complex molecules, including potential pharmaceutical agents or biochemical probes.

**Scheme 1:**
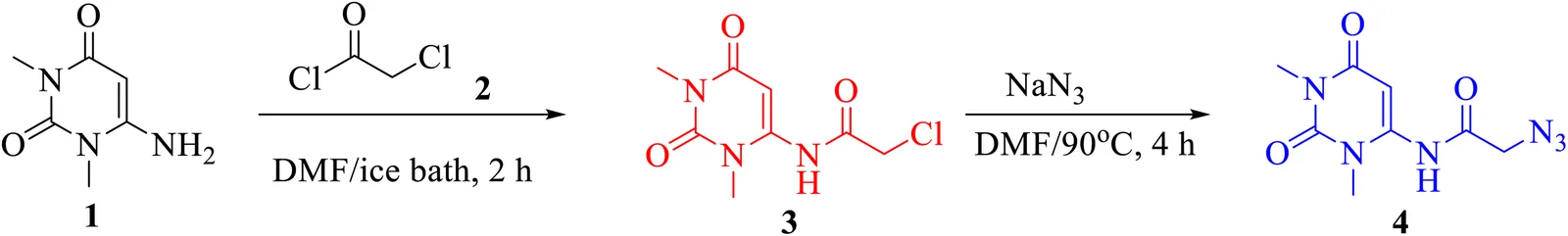
Synthesis of azido compound 4.

The molecular ion of compound 4 was observed at *m*/*z* = 238 (M^+^, 28%). The presence of azide groups is evident in the IR spectrum (strong peak at *ν*_max_ = 2110 cm^−1^). The ^1^H NMR spectrum of 4 showed five singlet signals for two methyl groups, CH_2_, CH-5 and NH group at *δ*_H_ = 3.12, 3.28, 4.52, 8.42 and 10.76 ppm, respectively. The ^13^C NMR spectrum of 4 revealed 2CH_3_, CH_2_, C-5, C-6, ^2^C=O, ^4^C=O amidine carbonyl carbons at *δ*_C_ = 27.5, 29.6, 51.6, 89.0, 149.5, 158.1, 161.2 and 192.2 ppm, respectively.

Subsequent synthesis of novel triazole-linked hybrid compounds 6a,b was achieved *via* a Copper(i)-catalyzed Azide–Alkyne Cycloaddition (CuAAC) reaction, commonly known as Click Chemistry. This highly efficient and regioselective reaction serves to connect the pyrimidine-2,4-dione scaffold, previously functionalized with an azide group of 4 (Scheme 2), with the prepared alkyne-substituted benzoxazole and benzimidazole derivatives 5a,b.^30,31^ The key transformation in this scheme is the CuAAC reaction of 2-azido-*N*-(1,3-dimethyl-2,6-dioxo-1,2,3,6-tetrahydro-pyrimidin-4-yl)acetamide (4) with 5a,b. The reaction was performed in dimethylformamide (DMF) at room temperature for 20 h, utilizing sodium ascorbate and copper(ii) sulfate (CuSO_4_) to generate the active copper(i) catalyst *in situ*.

**Scheme 2:**
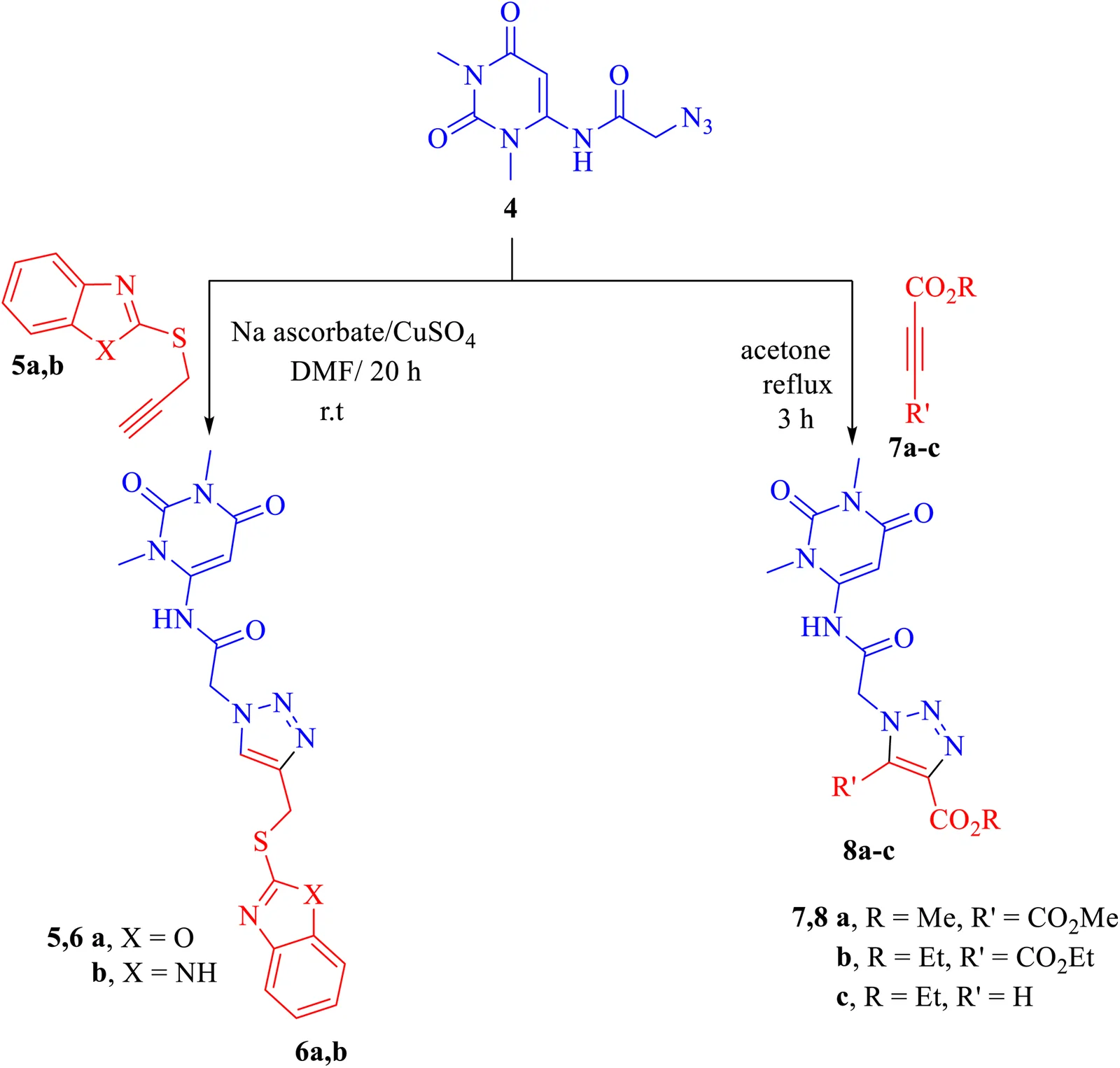
Synthesis of various 1,2,3-triazole derivatives 6a,b and 8a–c.

The azide group of 4 and the terminal alkyne group on compounds 5a,b are ideal partners for this [3 + 2] cycloaddition. The reaction proceeds with high regioselectivity, exclusively forming the 1,4-disubstituted 1,2,3-triazole ring. This triazole linkage acts as a stable and rigid linker between the two pharmacologically relevant moieties: the pyrimidine-2,4-dione and the benzoxazole/benzimidazole/benzothiazole systems. The characterization data, including ^1^H NMR, ^13^C NMR, mass spectrometry, and elemental analysis, confirmed the chemical structures of our new synthesized compounds 6a,b. All spectral data for synthesized products 6a,b clarify that they are formed from one molecule of terminal alkynes 5a,b and one molecule of 4 without any elimination. For example, ^1^H NMR spectral data of compound 6a was identified as 2-(4-((benzo[*d*]oxazol-2-ylthio)methyl)-1*H*-1,2,3-triazol-1-yl)-*N*-(2,6-dioxo-1,2,3,6-tetrahydro-pyrimidin-4-yl)acetamide revealed seven singlet signals at *δ*_H_ = 3.18, 3.31, 4.71, 5.76, 8.06, 8.42, 10.58 ppm due to the two methyl groups, SCH_2_, NCH_2_, CH-5, triazole CH_5_ and NH groups, respectively. The ^13^C NMR spectrum revealed represented signals at *δ*_C_ = 26.6, 27.6, 29.7, 58.4, 89.0, 125.7, 141.3, 158.1, 161.5, 163.6, 189.4 ppm corresponding to 2 CH_3_, SCH_2_, NCH_2_, C-5, triazol C-5, triazol C-4, C-6, ^2^C=O, ^4^C=O and NHCO, respectively.

Then we sought for the preparation of novel 1,2,3-triazole derivatives 8a–c from uracil-containing azide precursor 4 and various alkyne derivatives 7a–c (Scheme 2). The starting material, 2-azido-*N*-(1,3-dimethyl-2,6-dioxo-1,2,3,6-tetrahydropyrimidin-4-yl)acetamide (4), features of uracil core substituted with an acetamide bearing an azide group. This azide functionality is crucial for the subsequent cycloaddition reaction. The reaction proceeds with the treatment of azide 4 with dimethyl but-2-ynedioate (DMAD) and its analogues 7a–c under reflux conditions in acetone. The alkyne derivatives are characterized by varying ester groups (R and R′) at the terminal positions. The reaction yields the corresponding 1,2,3-triazole derivatives 8a–c. Specifically, the product dimethyl 1-(2-((1,3-dimethyl-2,6-dioxo-1,2,3,6-tetrahydropyrimidin-4-yl)amino)-2-oxoethyl)-1*H*-1,2,3-triazole-4,5-dicarboxylate (8a) was formed when DMAD (7a) was used. Scheme 2 shows that the reaction is a [3 + 2] cycloaddition between the azide and the alkyne, forming the five-membered 1,2,3-triazole ring. The corresponding compound 8a was isolated in 80% yield (Scheme 2). The IR spectrum was devoid of the azide absorption. The triazole ester carbonyls appeared at *ν*_max_ = 1736 and 1708 cm^−1^, amidine carbonyls appeared at *ν*_max_ = 1693, 1642 and 1614 cm^−1^, whereas the amino group absorbed at 3335 cm^−1^. The ^1^H NMR spectrum exhibited two singlet peaks, which appeared at *δ*_H_ = 3.20, 3.32 for the two *N*–CH_3_. Besides these, two singlet peaks at *δ*_H_ = 3.82, 3.89 corresponding to two triazole methyl ester protons. In addition, three singlet peaks at *δ*_H_ = 6.04, 8.49 and 10.45 ppm, each integrating for protons, corresponding to methylenes, uracil CH-5 and amidine NH, respectively. In ^13^C NMR spectrum also, there are five carbonyl carbon signals appeared at *δ*_C_ = 158.1, 160.6, 161.7 and 187.7 besides other signals like two N–CH_3_ at *δ*_C_ = 27.6 and 29.7 ppm, two methoxy groups appeared at *δ*_C_ = 52.6 and 53.2 ppm. In addition to five carbons appeared at *δ*_C_ = 59.3, 88.9, 130.4, 139.4 and 149.5 ppm for CH_2_, uracil C-5, triazole C-4, triazole C-5 and triazole C-6.

Scheme 3 illustrates the synthesis of novel 1,2,3-triazole derivatives 10a–c featuring uracil moiety. This transformation involves the reaction of 2-azido-*N*-(1,3-dimethyl-2,6-dioxo-1,2,3,6-tetrahydro-pyrimidin-4-yl)acetamide (4) with various 1,3-dicarbonyl compounds 9a–c. The reactions were conducted in ethanol at 50 °C, in the presence of triethylamine (Et_3_N) as a base. Three different 1,3-dicarbonyl compounds were employed to demonstrate the versatility of this reaction. For compound 9a, pentane-2,4-dione (acetylacetone) was used, leading to the formation of product 10a. When ethyl acetoacetate (9b) was reacted with azide 4, compound 10b was obtained. Similarly, the reaction of ethyl benzoylacetate (9c) with azide 4 yielded compound 10c. The general structure of the triazolo–pyrimidine-acetamide derivatives 10a–c indicates the successful formation of the 1,2,3-triazole ring. The structures for all obtained products are fully consistent with their spectral data including ^1^H NMR, ^13^C NMR spectrum, mass spectrometry, in addition to and elemental analysis. To confirm our results, we choose compound 10a, which was assigned as 2-(4-acetyl-5-methyl-1*H*-1,2,3-triazol-1-yl)-*N*-(1,3-dimethyl-2,6-dioxo-1,2,3,6-tetrahydropyrimidin-4-yl)acetamide. Mass spectrometry and elemental analysis for compound 10a indicate that it has the molecular formula of C_13_H_16_N_6_O_4_ with *m*/*z* 320, which indicate that it is the result of a combination between one molecule of 2-azido-*N*-(1,3-dimethyl-2,6-dioxo-1,2,3,6-tetrahydropyrimidin-4-yl)acetamide (4) and one molecule of acetyl acetone (9a) with the elimination of one molecule of H_2_O. The ^1^H NMR spectrum for compound 10a showed seven singlet signals with chemical shifts at *δ*_H_ = 10.50, 8.00, 5.79, 3.34, 3.30, 3.10 and 2.26 ppm, which were assigned as NH, H-5_triazol_, CH_2_, NCH_3_, COC*H*_3_, NC*H*_3_ and C*H*_3_, respectively. The four methyl groups were further confirmed from their ^13^C NMR spectrum, which gave signals at *δ*_C_ = 29.7 (NCH_3_), 27.7 (NCH_3_), 26.9 (COCH_3_), and 10.0 ppm (CH_3_). The reactions under both types of conditions are regioselective, and the regioselectivity of this type is consistent with the precedent reported by Zeghada *et al.*^32^

**Scheme 3:**
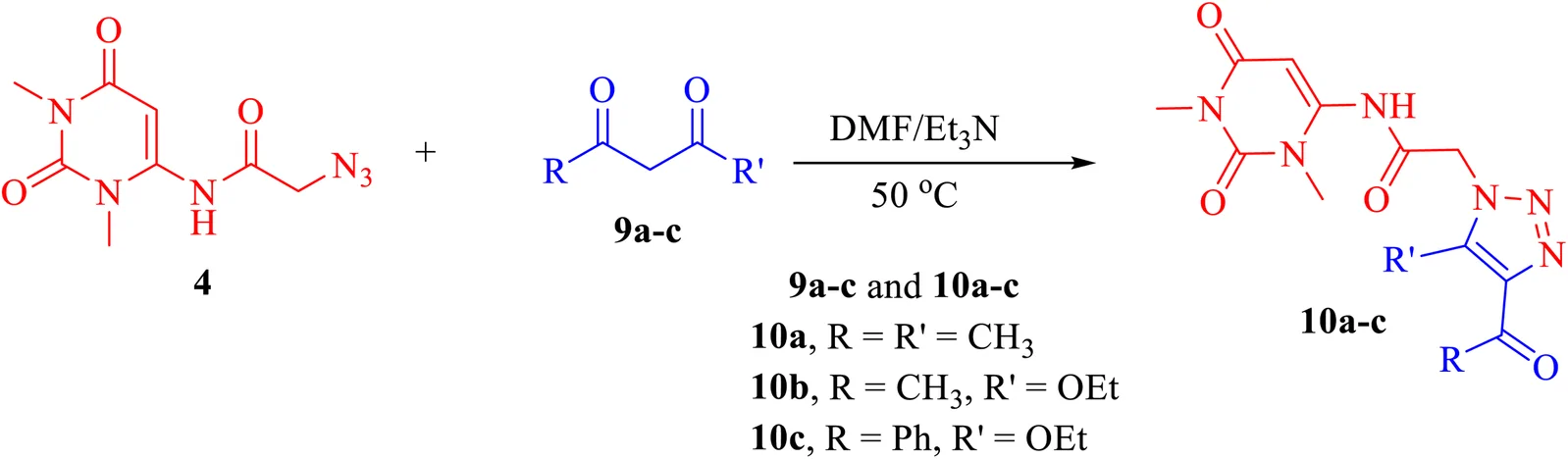
Synthesis of 1,2,3-triazolopyrimidinoacetamides 10a–c.

The proposed mechanism for the formation of compounds 10a–c through catalyzation of triethylamine (Et_3_N) during the condensation of an azide compound 4 with active-methylene 1,3-dicarbonyl compounds 9a–c, was based on an initially abstracting of the acidic methylene proton of the 1,3-dicarbonyl compound by Et_3_N to afford the resonance-stabilized enolate A. The carbon-centered enolate attacks the terminal electrophilic nitrogen of azide 4 with a proton transfer and tautomerization then generate a conjugated intermediate containing the three azide-derived nitrogen atoms and the two carbon atoms that become C4 and C5 of the triazole ring to form the cycloadduct 12. Et_3_N would then promote the subsequent proton-transfer and elimination steps. These dehydration reactions would then proceed in three steps: (i) a protonation at the keto carbonyl oxygen atom of 12, which corresponds to the most basic centers, (ii) an E1 elimination of water at the hydroxy group of the protonated intermediate 13, and (iii) a proton elimination at the vicinal C5 carbon atom of the formed intermediate 14 to re build the C4–C5 double bond of the enol, thus yielding the final 1,2,3-triazoles 10 (Scheme 4).

**Scheme 4:**
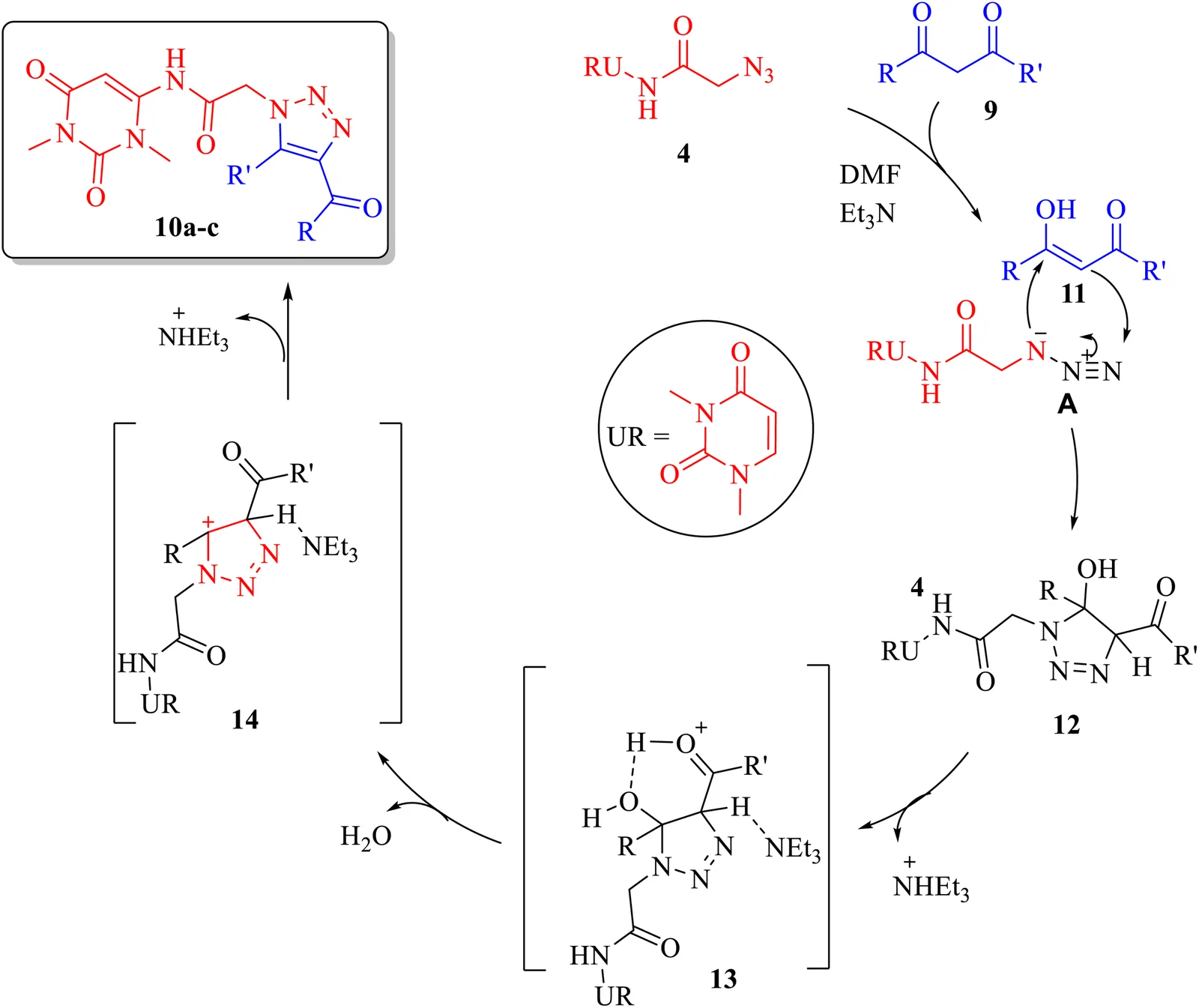
Proposed mechanism for the formation of compounds 10a–c.

### 2.2. Biology

#### 2.2.1. Cell viability assay

MTT assay was used to assess the viability of human cancer cell lines (MCF7, HT29, PANC1 and A549 cells) and the normal Vero cell line after 48 h incubation at the concentration tested.^15,33^ The synthesized compounds (3, 4, 6a,b, 8a–c and 10a–c) were tested for their *in vitro* anti-proliferative activity against four human cancer cell lines (MCF-7, HT29, PANC1, A549 cells) and one normal cell line (Vero cells) by cell viability assay, where doxorubicin was used as a reference drug (Table 1 and Fig. 4). The results showed a wide range of cytotoxicity for the derivatives tested. Compound 3 turned out to be the most remarkably potent compound, showing broad-spectrum activity with a very high inhibition of cell viability in all cancer lines (*e.g.*, 7.64% and 3.98% viability in MCF-7 and HT29 respectively). At the single screening concentration, compound 3 reduced PANC1 and A549 viability more than doxorubicin did. Moderate anti-proliferative profiles were observed in derivatives 6b, 8c and 10a with significant activity against PANC1 and MCF-7, respectively. On the other hand, the anti-cancer activity of compounds such as 8b and 10c was very low, with 10c showing an unfavorable anti-cancer activity in regards to normal Vero cells (6.23% viability). The overall average suppression of cancer cell viability across the four tested tumor lines, indicating the general descending order of potency of the synthesized compounds is: 3 > 6b > 8c > 10a > 10b > 8a > 4 > 6a > 10c > 8b.

**Table 1:** Results of cell viability assay of 3, 4, 6a, 6b, 8a–c and 10a–c

Compd	% Of cell viability				
	MCF-7	Vero	HT29	PANC1	A549
3	7.64	50.02	3.98	20.61	30.43
4	49.86	99.82	93.5	71.16	84.17
6a	76.43	97.14	91.71	41.51	90.04
6b	76.89	30.6	85.07	39.44	53.56
8a	48.9	72.85	91.21	48.53	92.27
8b	75.64	79.95	96.29	85.32	95.24
8c	43.65	72.44	95.38	46.92	75.63
10a	49.45	90.5	92.12	46.0	78.17
10b	50.64	36.88	97.57	51.07	74.83
10c	49.45	6.23	99.68	81.17	83.06
Doxorubicin	7.91	99.81	3.40	55.28	40.11

**Fig. 4:**
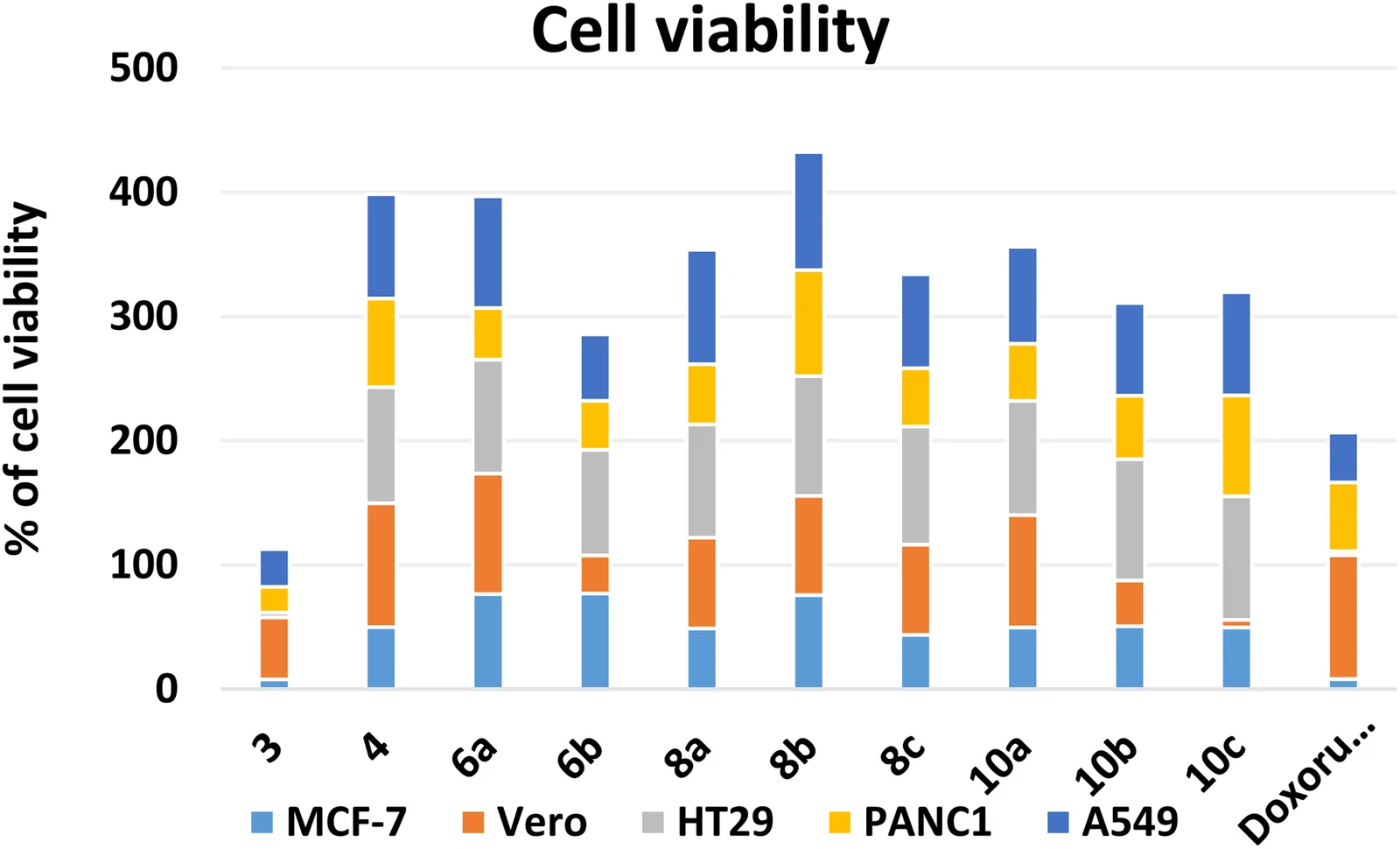
Percentage of cell viability of 3, 4, 6a, 6b, 8a–c and 10a–c.

#### 2.2.2. Antiproliferative assay

Half-maximal inhibitory concentration (IC_50_) is one of the most important parameters used to assess the cytotoxic activity of potential anti-cancer drugs; the lower the concentration of the drug required to inhibit the growth of half the population, the more potent the drug will be in terms of antiproliferative activity.^34,35^ The synthesized derivatives (3, 4, 6a,b, 8a–c, and 10a–c) were tested for their *in vitro* antiproliferative activity against four human cancer cell lines (MCF-7, HT29, PANC1, and A549) along with the normal Vero cell line, using doxorubicin as a standard chemotherapeutic drug standard. The results are summarized as half-maximal inhibitory concentration (IC_50_ in µM) in Table 2 and Fig. 5. Overall, the compounds showed a wide range of cytotoxicities. Compound 3 was the most effective broad-spectrum agent with relatively low IC_50_ values of 30.88, 74.89, 24.22 and 21.25 µM on the MCF-7, HT29, PANC1 and A549 cell lines, respectively. Notably, it was highly effective against PANC1 and A549 cells (IC_50_ = 24.22 and 21.25 µM), approaching the potency of doxorubicin (11.18 and 16.21 µM). In addition, a notable activity was found for compound 10c against the colon cancer cell line HT29 (IC_50_ = 9.38 µM) approaching the potency of doxorubicin (IC_50_ = 5.91 µM). It is interesting to note that 10c showed a much more favorable safety profile with low toxicity towards normal Vero cells (IC_50_ = 103.48 µM) as compared to doxorubicin (IC_50_ = 39.43 µM), giving a Vero/HT29 selectivity index of about 11, compared with about 7 for doxorubicin. Moderate to weak activities were also found with other derivatives, compounds 10a, 8c and 10b with moderate inhibition against MCF-7. In contrast, 8b was the least active against the cancer panel, and had high safety margins on normal Vero cells (IC_50_ > 190 µM), in addition to 4 and 8a. The general descending order of antiproliferative activity for the synthesized series is (based on the average IC_50_ values obtained on the four cancer cell lines): 3 > 6b > 10a > 10b > 10c > 8c > 6a > 8a > 8b > 4.

**Table 2:** Results of antiproliferative activity as IC_50_ ± SE (µM) of 3, 4, 6a, 6b, 8a–c and 10a–c

Compd	Antiproliferative activity IC_50_ ± SE (µM)				
	MCF-7	HT29	PANC1	A549	Vero
3	30.88 ± 0.19	74.89 ± 0.30	24.22 ± 0.12	21.25 ± 0.50	62.47 ± 0.87
4	86.54 ± 1.11	155.69 ± 0.95	181.20 ± 0.33	147.85 ± 4.58	205.06 ± 3.06
6a	83.75 ± 0.55	100.70 ± 0.53	156.76 ± 1.01	65.95 ± 1.68	152.59 ± 1.60
6b	85.94 ± 0.43	62.88 ± 0.70	137.58 ± 1.09	66.09 ± 1.37	88.89 ± 1.64
8a	59.94 ± 0.79	81.83 ± 0.20	190.23 ± 3.25	78.53 ± 0.80	194.65 ± 2.94
8b	113.98 ± 1.43	87.04 ± 0.78	103.35 ± 0.49	111.28 ± 1.19	192.48 ± 1.56
8c	60.36 ± 0.19	83.36 ± 0.55	180.37 ± 2.50	66.19 ± 2.16	136.50 ± 8.28
10a	57.99 ± 0.30	88.74 ± 0.35	149.88 ± 0.95	75.41 ± 0.25	95.99 ± 0.28
10b	62.15 ± 0.30	74.93 ± 5.28	156.81 ± 0.66	88.65 ± 1.23	119.38 ± 1.80
10c	75.32 ± 0.75	9.38 ± 0.31	147.81 ± 0.64	156.06 ± 2.52	103.48 ± 1.36
Doxorubicin	7.42 ± 0.07	5.91 ± 0.08	11.18 ± 0.09	16.21 ± 0.02	39.43 ± 0.16

**Fig. 5:**
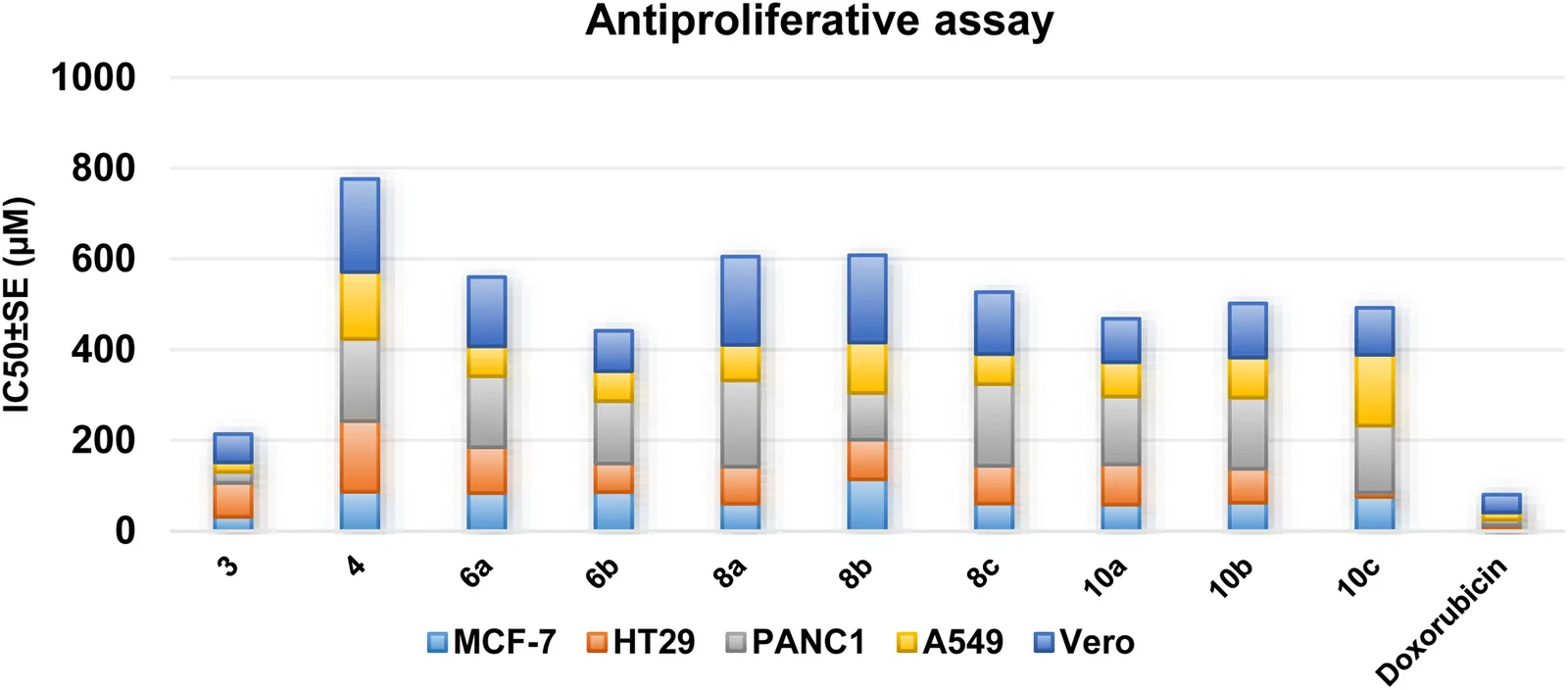
Antiproliferative activity as IC_50_ ± SE (µM) of compounds 3, 4, 6a, 6b, 8a–c and 10a–c.

#### 2.2.3. EGFR inhibitory assay

*In vitro* inhibitory activity of tested compounds 3, 10a, and 10c was studied using the standard reference drug erlotinib (Fig. 6) that inhibits the Epidermal Growth Factor Receptor (EGFR). As depicted in the data, all of the evaluated compounds exhibited good anti-EGFR activity with IC_50_ values in the sub-micromolar range. Compounds 10a was the most effective inhibitor of the series with an IC_50_ value of 0.082 ± 0.002 µM, approximately 2.4-fold less potent than the reference drug erlotinib with an IC_50_ value of 0.034 ± 0.001 µM. Compound 3 also demonstrated strong inhibitory activity with an IC_50_ of 0.116 ± 0.003 µM, compound 10c had the lowest relative activity of the three test compounds with an IC_50_ of 0.189 ± 0.005 µM. The results indicate that 10a is a sub-micromolar EGFR inhibitor, although less potent than erlotinib, and is presented as a starting point for structural optimization rather than as an equipotent EGFR agent.

**Fig. 6:**
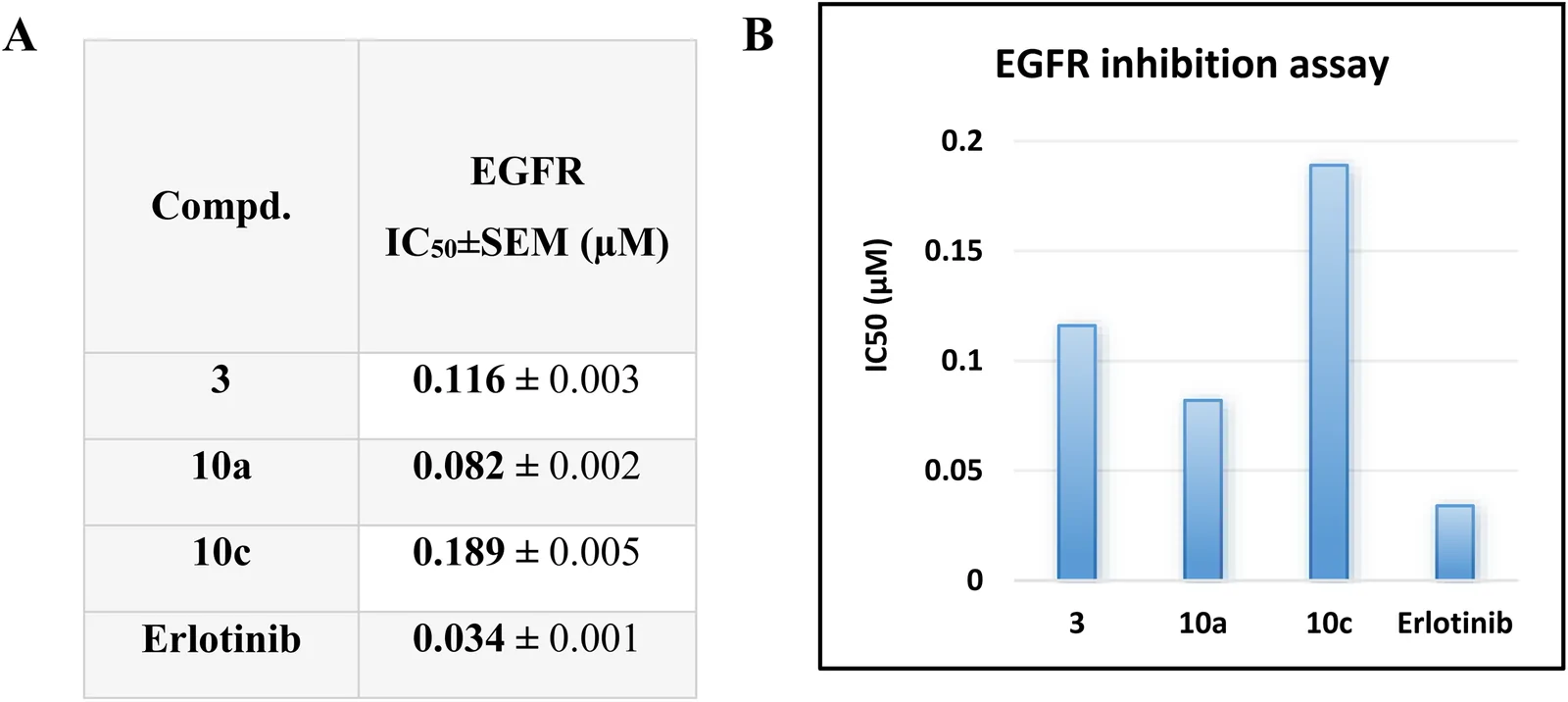
EGFR inhibition expressed (A) as IC__50__±SE (µM) of **1,2,**3-t riazole/ pyrimidine2,4-dione hybrids 3, 10a, 10c and Erlotinib as a reference. (B) refers to the corresponding histogram.

#### 2.2.4. CDK1 inhibitory assay

Synthetic compounds were found to be EGFR inhibitors and also tested against Cyclin-Dependent Kinase 1 (CDK1) with roscovitine as the reference compound (Fig. 7). To the credit of the three compounds tested 3, 10a, and 10c, all showed better inhibitory activity against the CDK1 enzyme than did the reference compound. As in the previous work, compound 10a was found to be the most active derivative in this assay, with an excellent IC_50_ value of 0.131 ± 0.004 µM, approximately 4.8-fold more potent than roscovitine (IC_50_ = 0.624 ± 0.029 µM). Compound 3 also showed outstanding activity with IC_50_ value of 0.163 ± 0.000 µM. Moreover, all the least active compound in this series, 10c (IC_50_ = 0.463 ± 0.017 µM) was able to beat the reference drug. These results indicate sub-micromolar CDK1 inhibition across the series, with 10a the most active member. As only a single wild-type enzyme was tested, these data do not establish selectivity or cellular CDK1 inhibition.

**Fig. 7:**
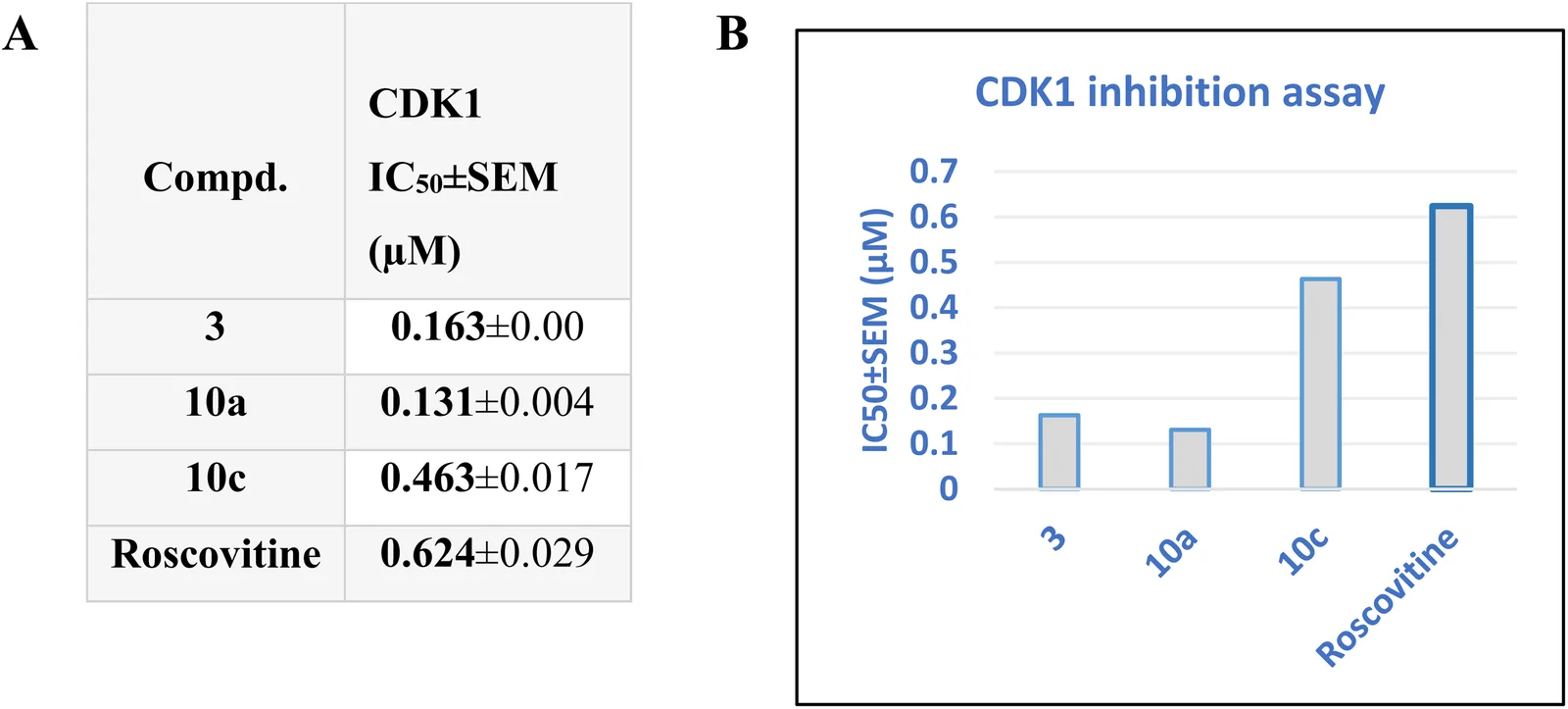
CDK1 inhibition expressed (A) as IC50±SE (µM) of 1,2,3-t riazole/ pyrimidine2,4-dione hybrids 3, 10a, 10c and roscovintine as a reference. (B) refers to the corresponding histogram.

#### 2.2.5. Caspase-3, BcL2 and BAX assay

The expression of important apoptotic markers (Caspase-3, Bcl-2 and BAX) were also analyzed in order to elucidate the underlying apoptotic mechanisms of the tested compounds in HT29 cells. IC_50_ and fold change of casp-3, BcL2 and BAX for 1,2,3-triazole/pyrimidine-2,4-dione hybrids 3, 10a, and 10c were as shown in Table 3 and Fig. 8. It was found that compound 10c is the most potent modulator among the agents tested to the assay as well as showed significant dose-dependent upregulation of these proteins. At its IC_50_ concentration (9.38 µM), 10c increased both the pro-apoptotic enzymes Caspase-3 (3.02-fold) and BAX (2.47-fold) when compared with the untreated control. Apparent is that this pro-apoptotic signal was accompanied by a 16.67-fold increase in the protein Bcl-2, which is anti-apoptotic, the origin of which is unknown; it may reflect a compensatory survival response, but oxidative stress was not measured. Compounds 10a and 3 also induced Caspase-3 and BAX less strongly: 1.92- and 1.93-fold for 10a at its IC_50_ (88.74 µM), and 1.79- and 1.89-fold for 3 at its IC_50_ (74.89 µM). Overall, caspase-3 was the most consistent apoptotic marker across compounds, with 10c the strongest inducer. Because Bcl-2 rose more than BAX, the BAX/Bcl-2 ratio (calculated from the mean values) decreased relative to control for all three compounds (for 10c from about 85 to about 13), so an intrinsic-pathway mechanism cannot be inferred from these markers; caspase activity and cleaved-caspase or PARP immunoblots are required.

**Table 3:** IC_50_ and fold change of casp-3, BcL2 and BAX for 1,2,3-triazole/pyrimidine-2,4-dione hybrids 3, 10a, and 10c

Code	Compd. conc. µM mL^−1^	Caspase-3		BcL2		BAX	
		IC_50_ ± SE ng mL^−1^	FLD	IC_50_ ± SE ng mL^−1^	FLD	IC_50_ ± SE ng mL^−1^	FLD
Control	—	0.038 ± 0.003	1	0.006 ± 0.002	1	0.510 ± 0.002	1
3	74.89	0.068 ± 0.002	1.789	0.039 ± 0.002	6.5	0.962 ± 0.002	1.886
	7.49	0.040 ± 0.004	1.053	0.017 ± 0.001	2.833	0.510 ± 0.001	1
10a	88.74	0.073 ± 0.004	1.921	0.021 ± 0.001	3.5	0.986 ± 0.001	1.933
	8.87	0.060 ± 0.003	1.579	0.021 ± 0.000	3.5	0.787 ± 0.002	1.543
10c	9.38	0.115 ± 0.000	3.026	0.100 ± 0.001	16.667	1.260 ± 0.002	2.471
	0.94	0.106 ± 0.003	2.789	0.039 ± 0.001	6.5	1.183 ± 0.002	2.320

**Fig. 8:**
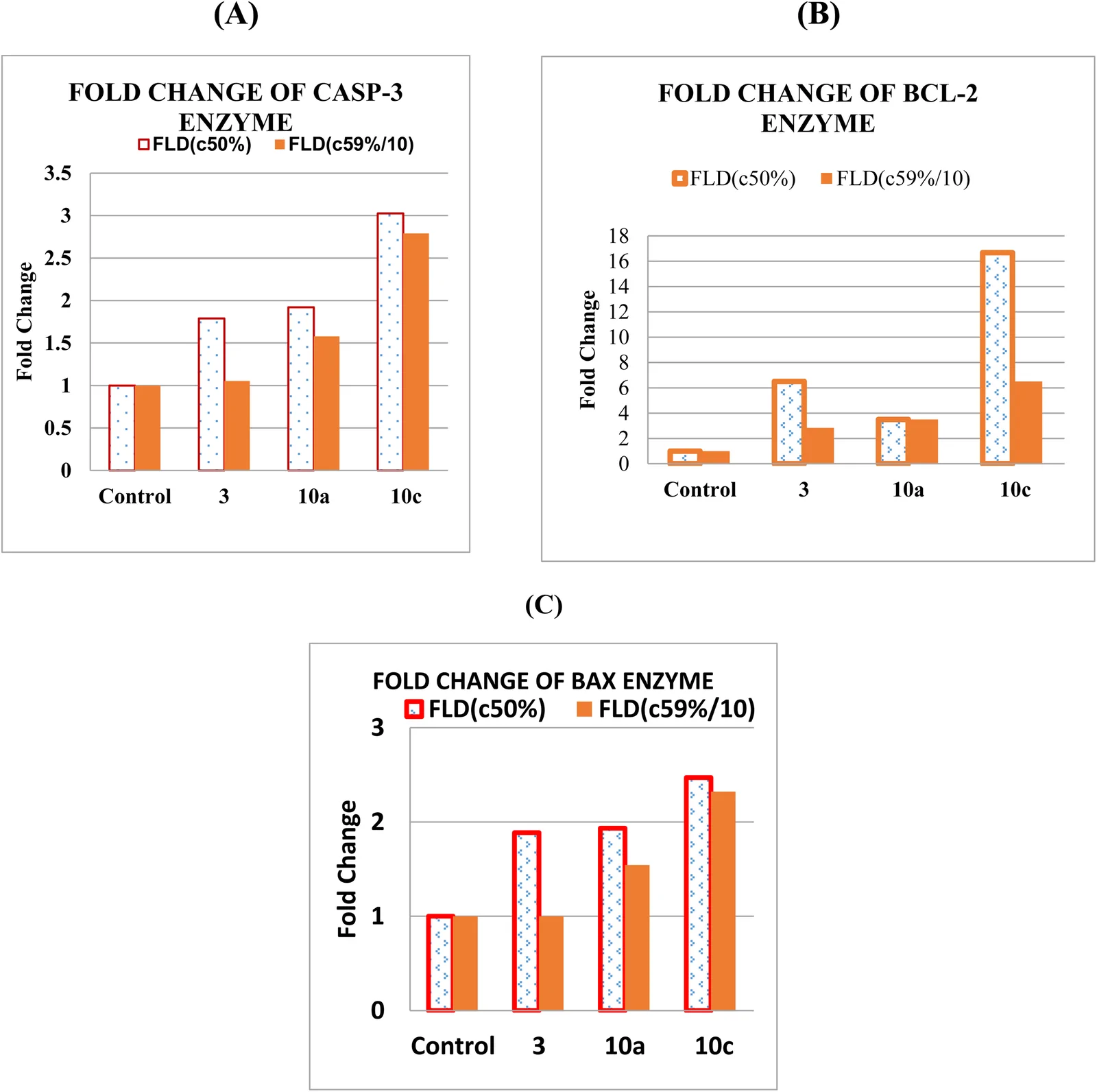
IC_50_ and fold change of (A) casp-3, (B) BcL2 and (C) BAX for 1,2,3-triazole/pyrimidine 2,4-dione hybrids 3, 10a and 10c.

#### 2.2.6. Cell cycle analysis

The results indicated that compound 10a was associated with accumulation at the G0/G1 phase of the cell cycle, with the proportion of cells in the G0/G1 phase increasing to 74.89%, compared to 60.04% in untreated control cells. The S-phase fraction decreased to 19.66% and the G2/M fraction to 5.45%, compared to 31.49% and 8.47%, respectively, in the control group. Consistent with this, compound 10a is associated with proliferation through G0/G1 accumulation. Because CDK1 mainly governs G2/M progression, G0/G1 accumulation is more consistent with suppression of EGFR-driven G1 progression (Table 4 and Fig. 9 and 10).

**Table 4:** The percentage of accumulation of cells at phases of cell cycle of HT29 cancer cells after treatment with 10a compared to control

Compd	DNA content			
	% G0–G1	% *S*	% G2/M	Comment
10a/HT29	74.89	19.66	5.45	Cell growth arrest @ G1/S
Cont. HT29	60.04	31.49	8.47	

**Fig. 9:**
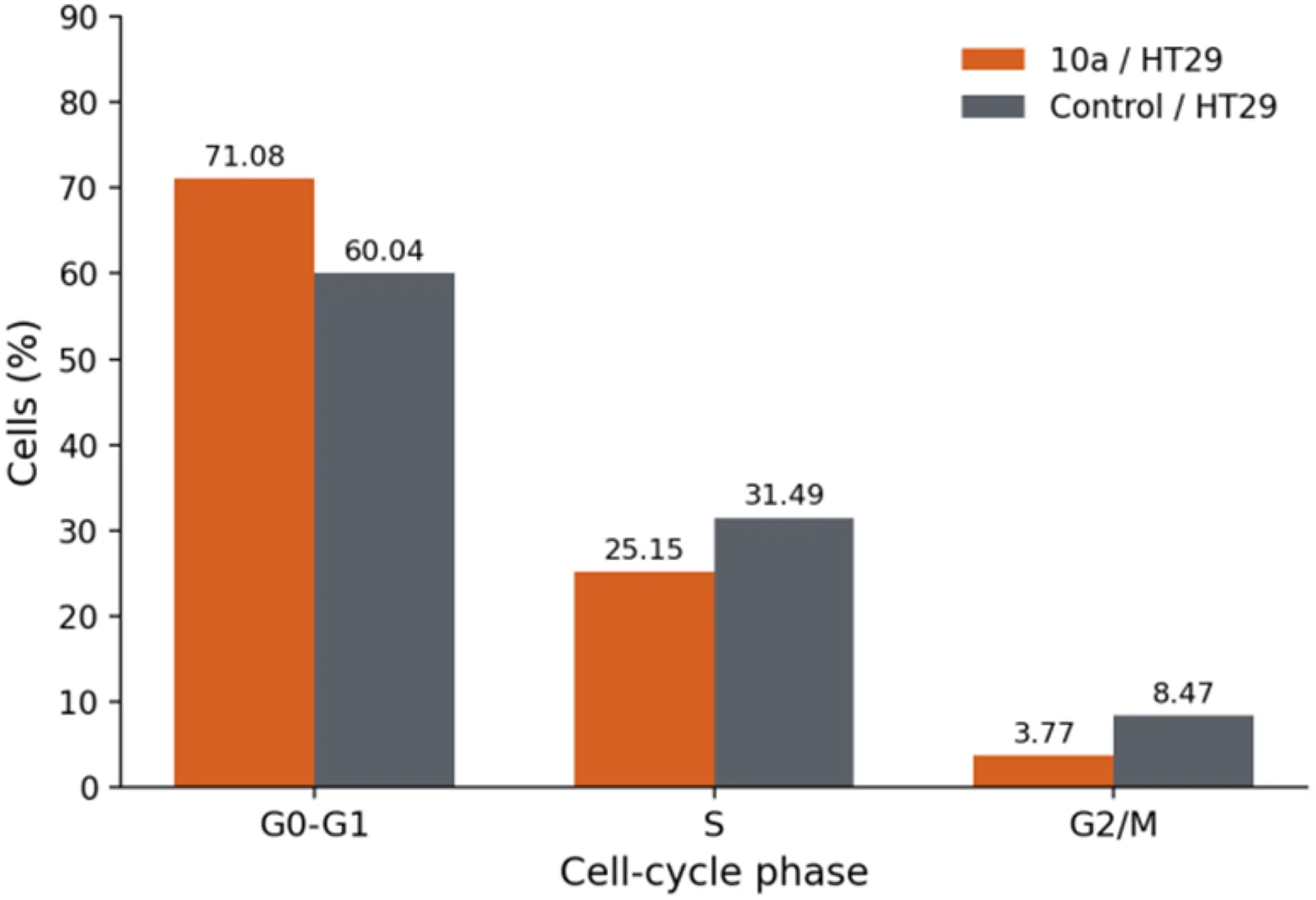
DNA content in cell cycle of HT29 cancer cells treated with 10a.

**Fig. 10:**
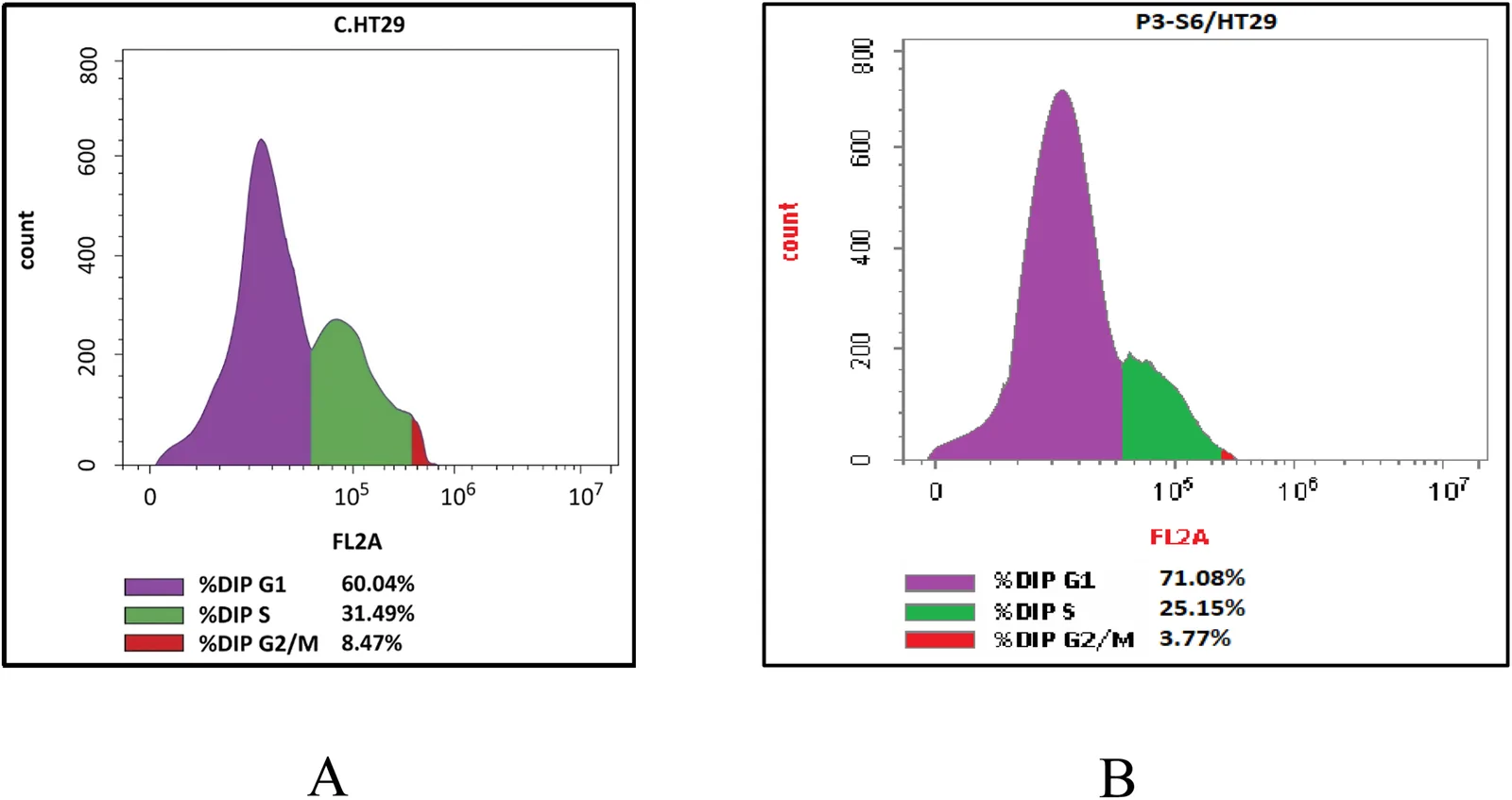
Cell cycle analysis of HT29 treated with DMSO control (A) and treated with 10a (B).

#### 2.2.7. Apoptosis analysis

The impact of compound 10a on cell death was evaluated through flow cytometry analysis, focusing on both apoptotic and necrotic stages in HT29 colorectal cancer cells. The results indicated that compound 10a induced cell death, with 3.61% of cells in the early apoptosis stage and 9.94% in the late apoptosis stage, giving 13.55% apoptotic cells in total. By comparison, untreated control cells exhibited minimal apoptosis, with only 0.91% in early apoptosis and 0.25% in late apoptosis, giving 1.16% apoptotic cells in total (3.76% when necrotic cells are included). Furthermore, necrosis was found to be higher in cells treated with compound 10a, with 3.87% of cells entering necrosis, compared to 2.60% in the control group. Thus, 10a increased the apoptotic fraction about 12-fold relative to control (13.55% *versus* 1.16%), with a smaller increase in necrosis (3.87% *versus* 2.60%) (Table 5 and Fig. 11 and 12).

**Table 5:** The effect of DMSO (control) and 10a on the percentage of Annexin V-FITC-positive staining in HT29 cells

Compd	Apoptosis			Necrosis
	Total	Early	Late	
10a/HT29	13.55	3.61	9.94	3.87
Cont. HT29	1.16	0.91	0.25	2.60

**Fig. 11:**
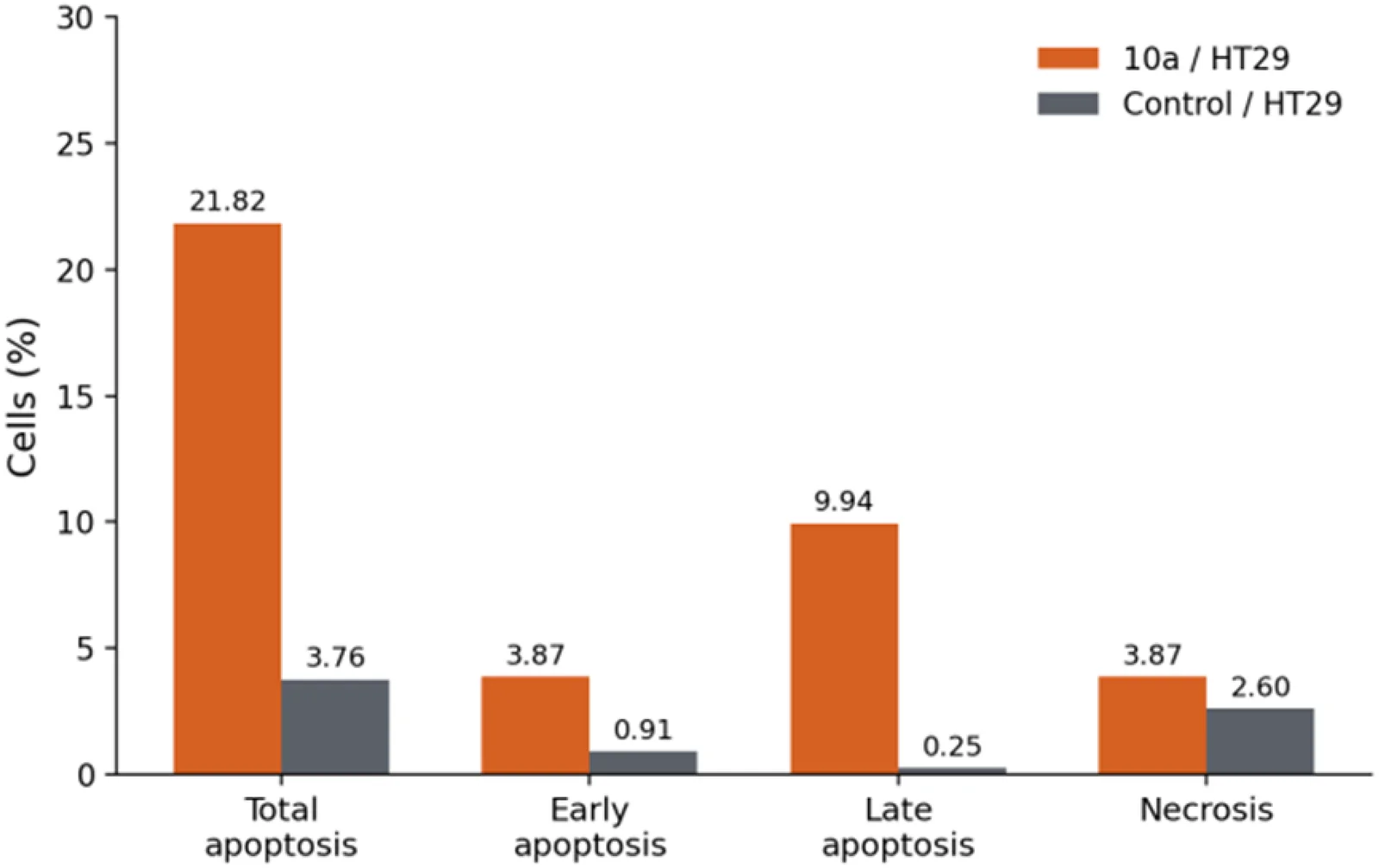
The ratio of early and late apoptosis and necrosis in HT29 cells upon treatment with 10a compared to DMSO control.

**Fig. 12:**
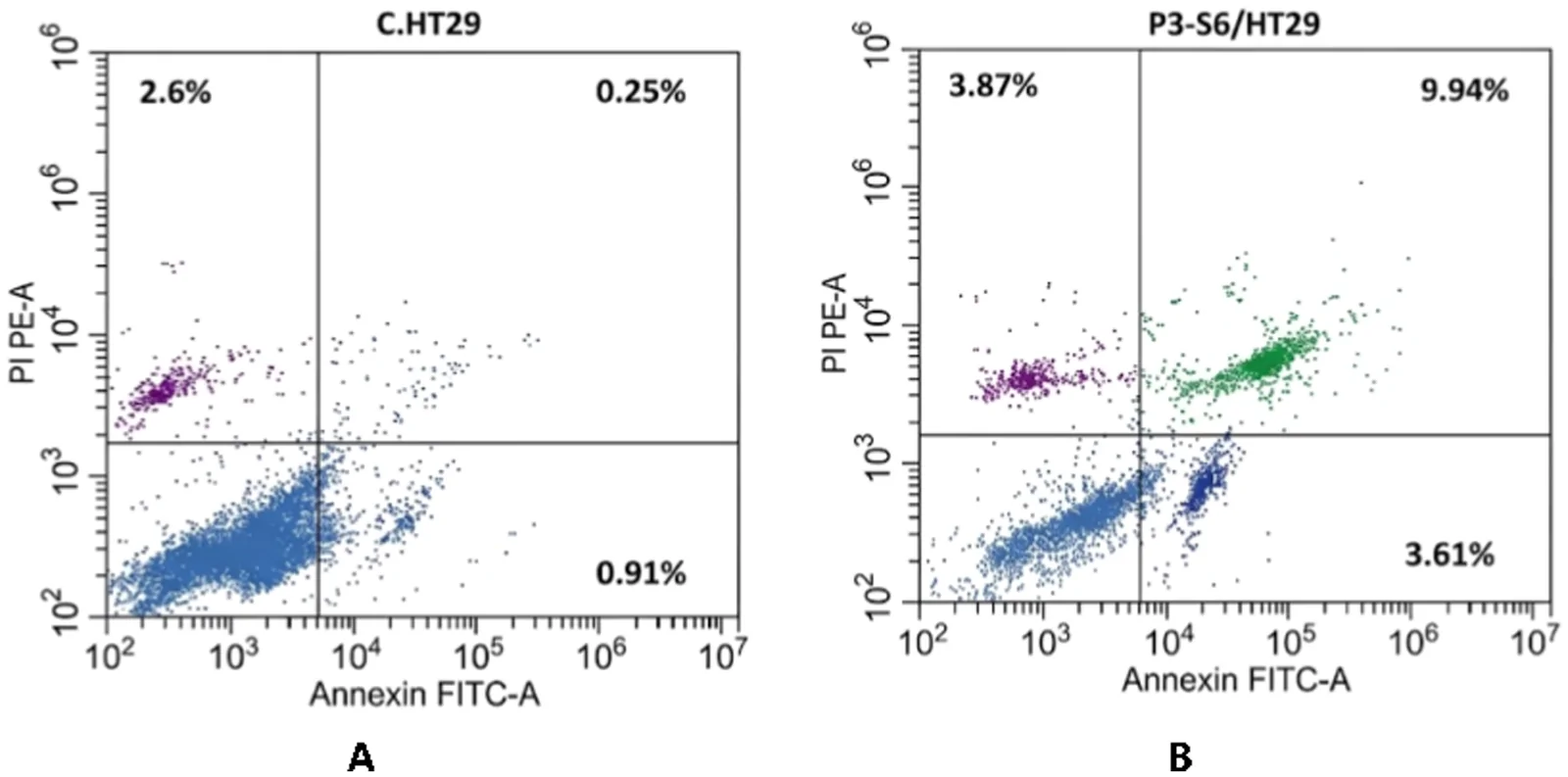
The effect of DMSO (A) and 10a (B) on the percentage of Annexin V-FITC-positive staining in HT29 cells.

#### 2.2.8. Docking studies

Molecular docking was used to predict plausible binding modes of compound 10a within the ATP-binding sites of EGFR (PDB: 1M17) and CDK1 (PDB: 4YC6). The docking protocol was first validated by redocking the co-crystallized ligands, erlotinib for EGFR and roscovitine for CDK1. The redocked poses reproduced the crystallographic conformations with heavy-atom RMSDs of 1.20 Å and 1.57 Å, respectively, both below the 2.0 Å threshold commonly used to judge pose reproduction (Table 6 and Fig. 13 and 14).^36,37^

**Table 6:** Docking scores, heavy-atom RMSDs and ligand–receptor interactions for erlotinib, roscovitine and 10a at the EGFR (PDB: 1M17) and CDK1 (PDB: 4YC6) active sites

Protein	Compd	Heavy-atom RMSD (Å)	*S* score (kcal mol^−1^)	Residue	C-Docker energy (kcal mol^−1^)	Interaction type	Length (Å)
EGFR	Erlotinib	1.20^a^	−8.99	MET 742	−0.8	H-donor	3.56
				CYS 773	−0.8	π–H	3.75
	10a	2.94	−6.64	MET 742	−0.4	H-donor	4.00
				CYS 773	−0.8	H-acceptor	3.14
				LEU 694	−0.7	π–H	4.36
				CYS 773	−0.9	π–H	4.60
CDK1	Roscovitine	1.57^a^	−7.23	ASP 146	−4.2	H-donor	2.91
				LYS 130	−0.7	π–H	3.48
				HIS 162	−0.0	π–π	3.97
	10a	1.70	−6.78	ASP 146	−4.2	H-donor	3.21
				ASP 146	−1.4	H-donor	3.31
				VAL 18	−0.6	π–H	4.34

a Heavy-atom RMSD of the redocked co-crystallized ligand. C-Docker energies are per-residue interaction contributions (kcal mol^−1^). Length is the interaction distance in Å.

**Fig. 13:**
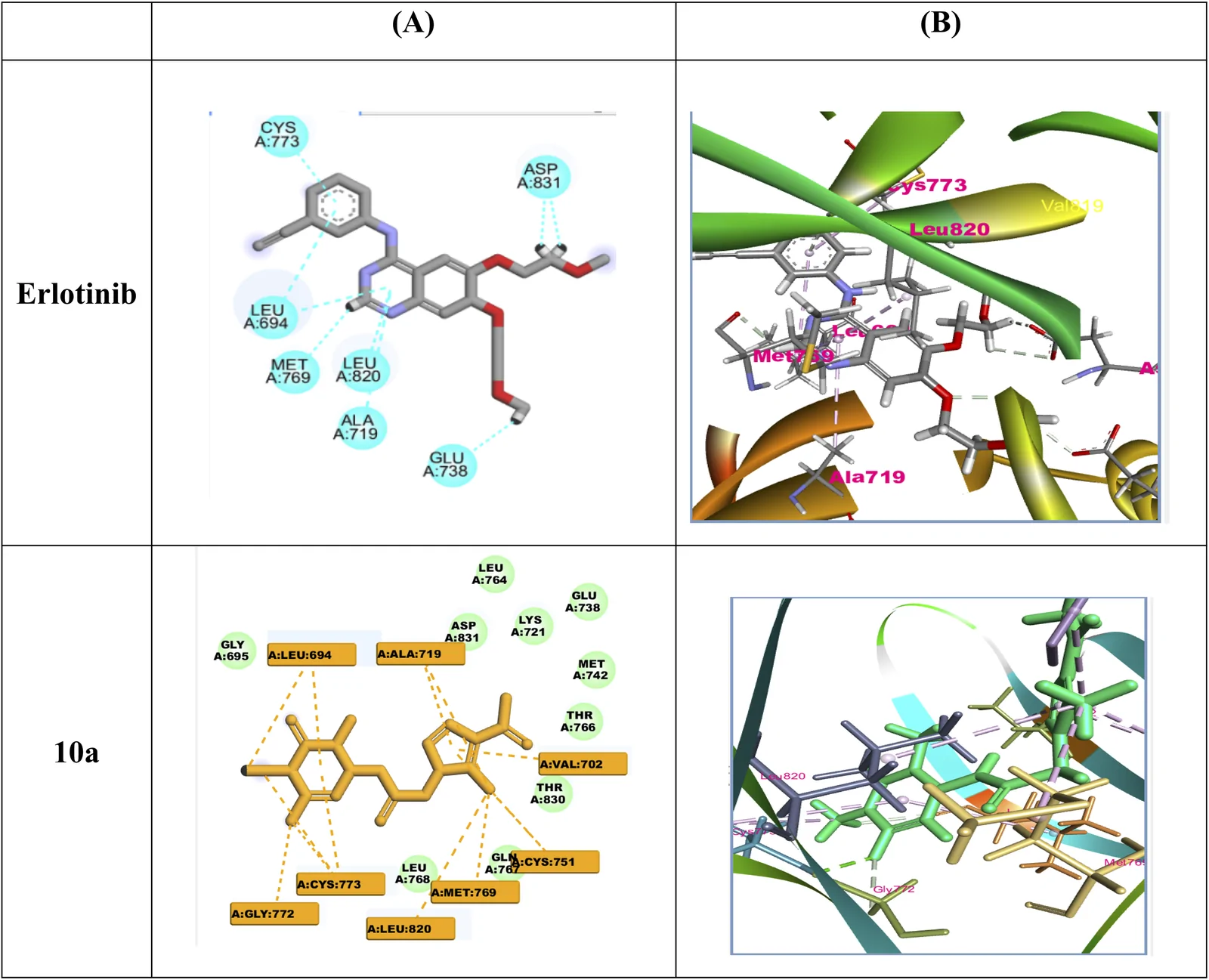
(A): 2D caption and (B): 3D caption of erlotinib and 10a showed the binding modes to the active site of EGFR active site (PDB: 1M17).

**Fig. 14:**
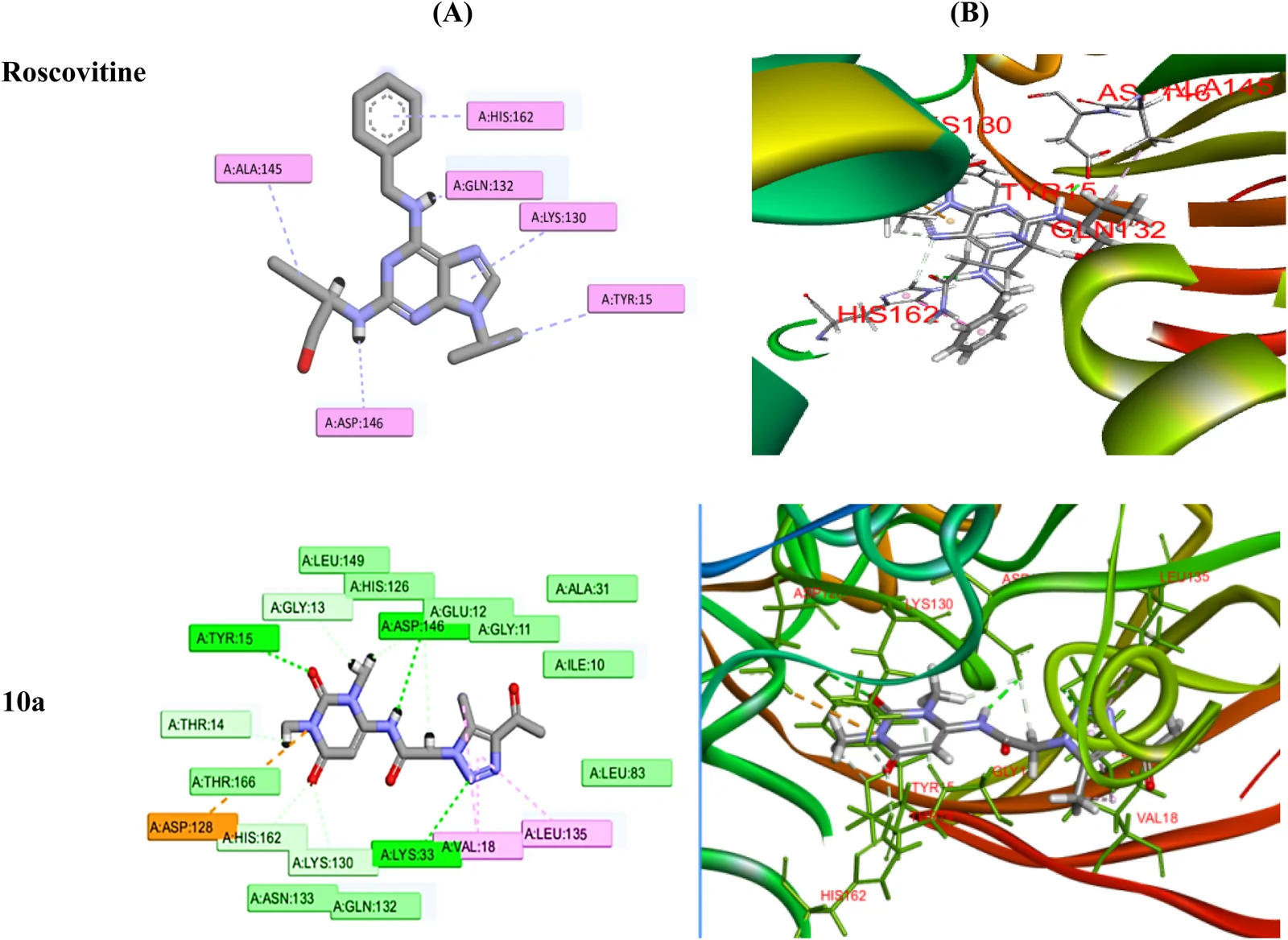
(A): 2D caption and (B): 3D caption of roscovitine and 10a showed the binding modes to the active site of CDK1 active site (PDB: 4YC6).

At the EGFR site, erlotinib gave an *S* score of −8.99 kcal mol^−1^, with a hydrogen-bond donor interaction to MET742 (3.56 Å) and a π–H interaction with CYS773 (3.75 Å). Compound 10a scored −6.64 kcal mol^−1^ and was predicted to retain the MET742 hydrogen-bond donor contact (4.00 Å) and to form a hydrogen-bond acceptor interaction with CYS773 (3.14 Å). It also made π–H contacts with LEU694 (4.36 Å) and CYS773 (4.60 Å). Its heavy-atom RMSD of 2.94 Å relative to the erlotinib pose indicates that 10a occupies a partly different region of the pocket than erlotinib, although it contacts the same key residues.

At the CDK1 site, roscovitine gave an *S* score of −7.23 kcal mol^−1^, with a hydrogen bond to ASP146 (2.91 Å), a π–H interaction with LYS130 (3.48 Å) and a π–π stacking interaction with HIS162 (3.97 Å). Compound 10a scored −6.78 kcal mol^−1^ and formed two hydrogen-bond donor contacts with ASP146 (3.21 Å and 3.31 Å), mirroring the hydrogen-bonding pattern of roscovitine, together with a π–H interaction with VAL18 (4.34 Å). Its heavy-atom RMSD of 1.70 Å relative to the roscovitine pose indicates a broadly similar binding orientation.

Docking scores are predictive only. The weaker EGFR score for 10a (−6.64 *versus* −8.99 kcal mol^−1^) is consistent with its weaker enzymatic inhibition (0.082 *versus* 0.034 µM for erlotinib). The CDK1 scores of 10a and roscovitine are close (−6.78 *versus* −7.23 kcal mol^−1^), whereas the enzyme assay shows about 4.8-fold greater potency for 10a (0.131 *versus* 0.624 µM). The docking model therefore does not explain the CDK1 potency difference, and the predicted binding modes require experimental confirmation.

#### 2.2.9. Normal Mode Analysis (iMODS)

Normal mode analysis (NMA) was performed with the iMODS server on both docked complexes (EGFR–10a and CDK1–10a). NMA models collective, low-frequency motions within an elastic-network approximation; it is not a molecular dynamics simulation and does not sample conformational ensembles over time. The molecular mobility and the structural dynamics of the docking complexes were calculated by using iMODS. iMODS is an online server that offers simple and flexible tools for the analysis of crucial dynamics, such as Eigen values, Variance, Covariance maps, *B*-factors and Elastic networks. The input files were the PDB files that were docked and submitted to the server and all parameters were left default until the results were displayed. iMODS uses Normal Mode Analysis (NMA) in combination with the coordinates of the docked complexes, to assess both the molecular motion and the structural flexibility of the biological macromolecules. The molecular motion and deformability are shown as colored arrows in every panel of Fig. 15A, with two clusters showing the directionality of NMA motion for both the EGFR–compound 10a and CDK1–compound 10a complexes. Interestingly, the CDK1–compound 10a complex has a distinct large red arrow, suggesting a more prominent conformational motion at the global level than in the EGFR complex with more localized conformational motions of its domains. Each residue (Cα atom) in the protein chain is shown to be distorted in a unique manner, with the colored hinges indicating the distinct nature of the distortions that contribute significantly to the deformability of the complex (Fig. 15B). In the case of the EGFR–compound 10a complex, the deformability values are fairly low and are relatively uniform for most of the residues (atom indices 0–260) with modest peaks generally located between residues 50–100 and 150–200. In contrast, the CDK1–compound 10a complex shows significantly stronger and more abundant deformability peaks throughout the sequence (from atom indices 0–320), indicating that compound 10a confers greater conformational flexibility on CDK1 as a whole. The server calculated the eigen values of 9.434286 × 10^−6^ for the EGFR–compound 10a complex and 1.313036 × 10^−4^ for the CDK1–compound 10a complex (Fig. 15D). Compared with the EGFR complex, the first normal mode for the EGFR–compound 10a complex has a considerably lower eigenvalue, indicating that the overall structure of the complex is more flexible. The higher value for the CDK1–compound 10a signifies a more rigid and more restricted molecular system in the lowest mode. Furthermore, the individual variance contributions of the normal modes are shown in Fig. 15E, where it is seen that the variance of each individual mode is inversely proportional to the eigenvalue. The first few modes of the low frequency modes contribute the largest percentage of variance (purple bars), and the cumulative variance (teal bars) quickly reaches 100% percent by the 6–8 modes, indicating that the most important modes of motion are well captured in the first few modes. The structural stability of docked molecules is shown in the *B*-factor plot (Fig. 15C) that compares the fluctuations observed in NMA with the *B*-factors from the experimental PDB structures (grey). The NMA-predicted *B*-factors for the EGFR–compound 10a complex show several large peaks, which are generally in good agreement with the crystal structures' experimental *B*-factors, indicating that the binding of compound 10a does not significantly alter the native flexibility profile of EGFR. In the case of the CDK1–compound 10a complex, the *B*-factors of NMA are larger, especially in the loop regions (residues 40–80 and 130–170). The PDB experimental data is similar, but less pronounced, as these regions of CDK1 are known to be dynamic, making it difficult to collect experimental data on them. C-Covariance Matrix: This is a colored display of correlated motions (red), uncorrelated motions (white) and anti-correlated motions (blue) in the covariance matrix (Fig. 15F). The correlation map for the EGFR–compound 10a complex shows that there are several large off-diagonal blocks of red color for the positive correlations, while there are large blocks of blue color for the anti-correlation. This indicates that the structural domains of EGFR move in an opposite but coordinated fashion when compound 10a binds to it. However, the diagonal-dominant red pattern of the 10a covariance map suggests more localized domain-specific correlated motions, and correlates more strongly with the bilobal architecture of CDK1. The elastic network model (Fig. 15G) signifies that the Cα atoms of the docking protein are connected by elastic springs of varying strength, with darker grey spots corresponding to stronger and firmer springs between pairs of atoms. The contact map for the EGFR–compound 10a complex reveals a widespread network of strong contacts, especially in the central active site region (residues 100–200), which is a reflection of the structural rigidity of the compound 10a at the active site region. The elastic network shows tight firm contacts around the N terminal lobe (0–100) and weaker, but still important contacts around the C terminal lobe (101–299) in the case of the CDK1–compound-10a complex. This is typical of the contact distribution observed within the CDK1 kinase pocket, and shows that compound 10a can be stably docked in the CDK1 binding pocket of both target proteins. Together, the NMA results indicate that the docked complexes are mechanically plausible in the normal-mode approximation. Stability over time was not assessed; a conventional MD simulation of at least 100 ns is required for that purpose and is listed as future work.

**Fig. 15:**
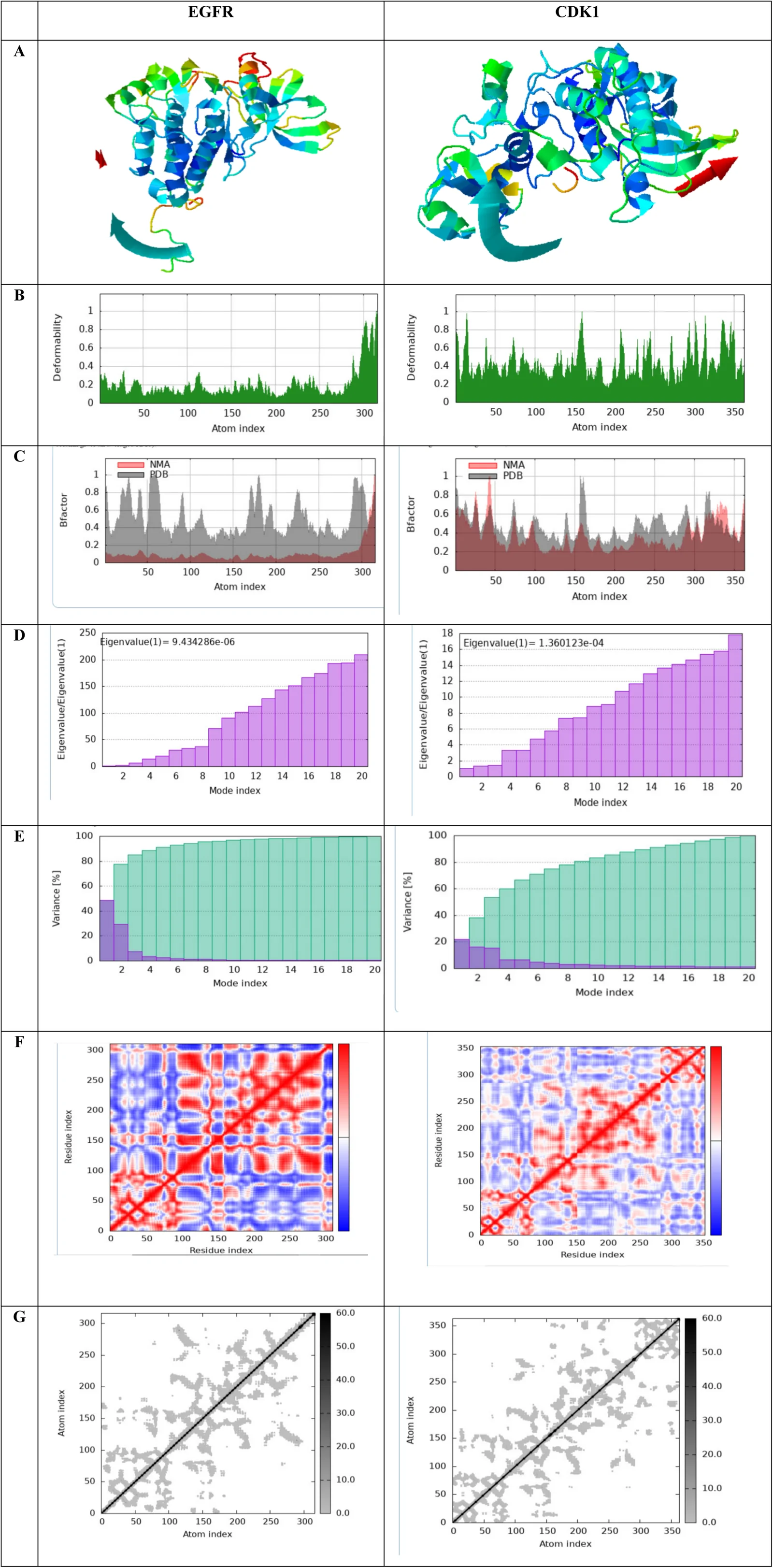
Results obtained from the docked complexes (1M17-ligand and -ligand, respectively) with compound 10a utilizing the iMODS server. (A), NMA of the docked complexes used to assess molecular mobility (two colored affine arrows indicating domain mobility); (B), deformability plots; (C), *B*-factor plots; (D), eigenvalues; (E), variance plots; (F), covariance matrix analysis; (G), elastic networks.

#### 2.2.10. Predicted ADMET studies

The identification of a promising therapeutic candidate requires not only potent biological activity but also favorable pharmacokinetic and toxicity profiles, collectively described as ADMET (Absorption, Distribution, Metabolism, Excretion, and Toxicity) properties.^35,38–40^ In recent years, several computational platforms have been developed to predict these parameters and facilitate early-stage drug discovery.^41^ Among these, ADMETlab 3.0 has emerged as a widely utilized and reliable web-based tool for evaluating the pharmacokinetic behavior of potential anticancer agents.^35^

Accordingly, the ADMET profile of compound 10a was assessed using ADMETlab 3.0. The radar plot indicates that 10a satisfies several drug-likeness rules (no Lipinski violations and no PAINS alerts), but it also shows several liabilities. However, the predicted topological polar surface area (TPSA ≈ 120.88 Å^2^) is high, which is generally associated with limited passive membrane permeability.

The predicted log *P* (−0.234) is low and predicted aqueous solubility (log *S* −1.442) is moderate. The predicted HIA and bioavailability score are both 0.0, Caco-2 permeability is weak (−4.945), and the predicted half-life is short (about 1.07 h). Importantly, the predicted hepatotoxicity (DILI 0.969; hepatotoxicity 0.612) is high and must be regarded as a serious liability. These *in silico* predictions do not establish a favorable pharmacokinetic profile; they identify the properties that future analogues must address (Fig. 16 and Table 7).

**Fig. 16:**
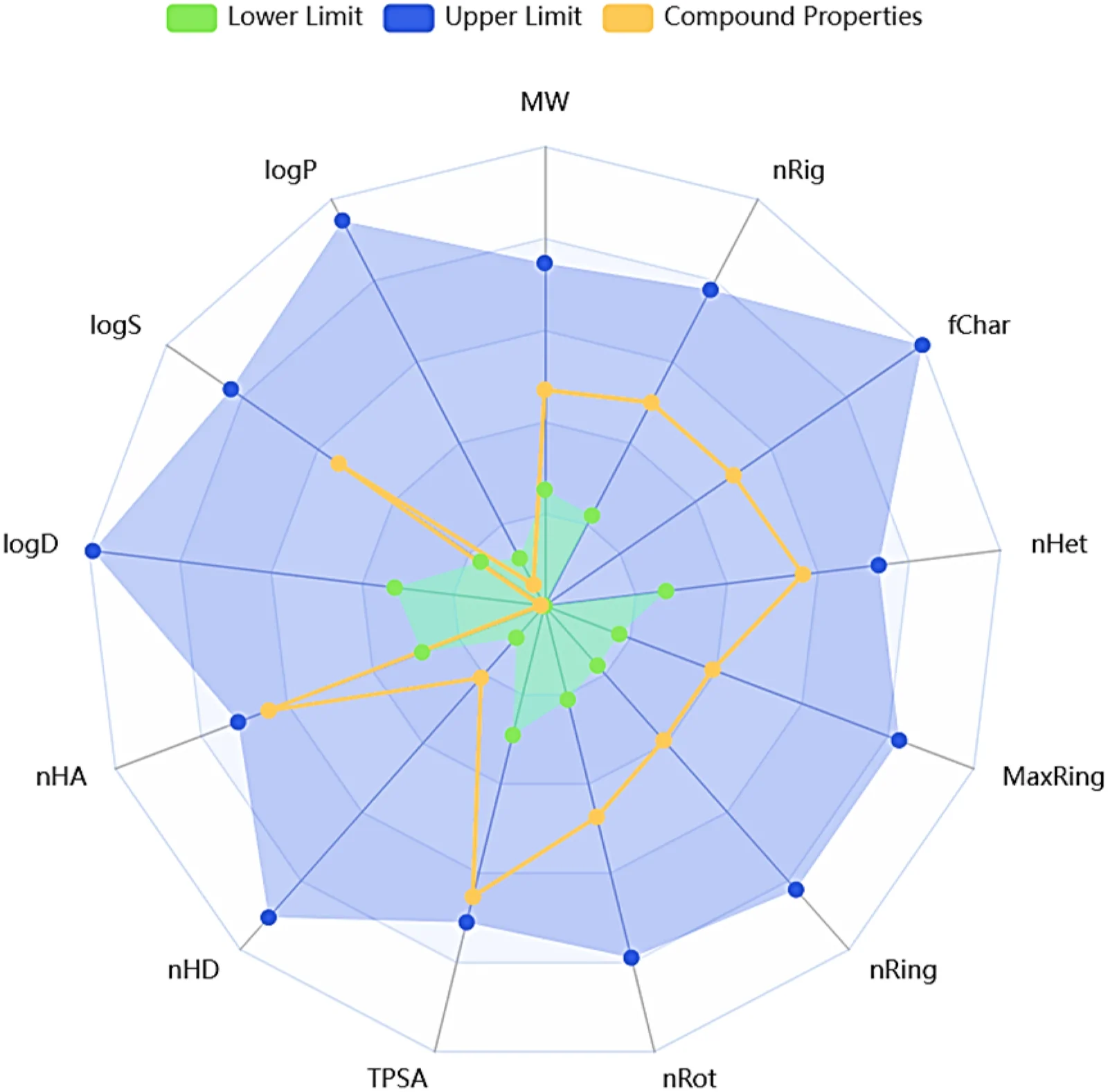
ADMET-based pharmacokinetic profile mapping of compound 10a generated using ADMETlab 3.0.

**Table 7:** Predicted physicochemical properties, ADMET profile, drug-likeness, and toxicity parameters of compound 10a obtained using ADMETlab 3.0

Property	10a
**Physicochemical properties**	
Molecular weight (g mol^−1^)	320.12
Heavy atoms (*n*Het)	10.0
Aromatic atoms/*n*Rig	15.0
Rotatable bonds (*n*Rot)	5.0
H-bond acceptors (*n*HA)	10.0
H-bond donors (*n*HD)	1.0
TPSA (Å^2^)	120.88
Molar refractivity/volume	298.978

**Lipophilicity**	
Log *P*	−0.234
Log *D* (pH 7.4)	0.032

**Water solubility**	
Log *S*	−1.442

**Pharmacokinetics**	
HIA	0.0
PPB	88.459%
VD	0.604 L kg^−1^
Fu	13.445%
BBB permeation	0.975 (yes)
Caco-2 permeability	−4.945
MDCK permeability	2.229 × 10^−5^ cm s^−1^
P-gp inhibitor	0.0

**Metabolism**	
CYP2C19 inhibitor	0.0
CYP2C9 inhibitor	0.0
CYP3A4 inhibitor	0.0
CYP2D6 inhibitor	0.0

**Excretion**	
Clearance (CLplasma)	1.496 mL min^−1^ kg^−1^
Half-life (*T*_1_/_2_)	1.068 h

**Toxicity**	
hERG	0.054
AMES	0.412
DILI	0.969
Hepatotoxicity	0.612
Carcinogenicity	0.402
Skin sensitization	0.174

**Drug-likeness**	
Lipinski rule	Accepted (0 alerts)
Bioavailability score	0.0
QED	0.708
SA score	2.0
PAINS alerts	0 alerts

#### 2.2.11. Structure-activity relationship (SAR)

This series of C-4′ triazole-substituted uracils delineates a clear relationship between structural modification, cellular fate, and dual EGFR/CDK1 inhibition. The chloroacetamide precursor, compound 3, exhibited aggressive, broad-spectrum cytotoxicity with only moderate enzymatic inhibition. This profile is consistent with, but not proven to arise from, nonspecific reactivity of the electrophilic chloroacetamide group; thiol reactivity and kinome selectivity of 3 were not measured, so compound 3 is treated here as a synthetic intermediate and mechanistic reference rather than a lead. Converting the azide into triazoles 6a,b narrowed this activity spectrum. The benzimidazole derivative 6b was more active than its benzoxazole analogue 6a, aligning with established trends in triazole-based kinase inhibitors although the structural basis for this difference was not examined.

The ester-functionalized triazoles 8a–c exhibited diminished potency (IC_50_ > 60 µM). A plausible explanation is that the polar ester groups increase the topological polar surface area and limit membrane permeability; this was not tested directly.

The most significant findings emerged from the 1,3-dicarbonyl derivatives. The 4-acetyl-5-methyl motif in 10a was the most active motif in this series, yielding sub-micromolar IC_50_ affinities for both EGFR (0.082 µM) and CDK1 (0.131 µM). Docking suggests that this substituent may engage the hinge region of the ATP pocket; this has not been verified experimentally. Replacing the 5-methyl group with a 5-ethoxy group (10b), or exchanging the 4-acyl for a 4-benzoyl aryl system (10c), severely compromised enzymatic activity. Interestingly, 10c demonstrated unexpected cytotoxicity selectivity against HT29 cells (IC_50_ = 9.38 µM) while sparing normal Vero cells (IC_50_ = 103.48 µM). The basis of this HT29 selectivity is unknown; it may reflect differences in cellular uptake, metabolism or target expression, and was not investigated further.

While 10a demonstrates highly potent enzymatic inhibition, its moderate antiproliferative IC_50_ values (58–150 µM) may reflect pharmacokinetic barriers, such as restricted passive diffusion, active *P*-glycoprotein efflux, or competition with high intracellular ATP concentrations. The G0/G1 accumulation and apoptosis observed in HT29 cells occur at micromolar concentrations close to the cellular IC_50_ and are consistent with, but do not demonstrate, engagement of EGFR or CDK1 in cells; compound stability, solubility and intracellular exposure were not measured.

## 3. Experimental

### 3.1. Chemistry

#### 3.1.1. General remarks

Uncorrected melting points were taken in a digital melting point apparatus (AELAB Touch Screen Melting Point Device DMP-800/DMP-600. The IR spectra were recorded by ATR technique (ATR = Attenuated Total Reflection) with a FT device (FT-IR Bruker IFS 88), Minia University, Faculty of science, The NMR spectra were measured in DMSO-*d*_6_ on a Bruker AV-400 spectrometer, 300 MHz for ^1^H, and 75 MHz for ^13^C; and the chemical shifts are expressed in *δ* (ppm), *versus* internal tetramethylsilane (TMS) = 0 for ^1^H and ^13^C, and external liquid ammonia = 0. The description of signals includes: s = singlet, d = doublet, t = triplet, q = quartet, m = multiplet. Mass spectra were recorded on a FAB (fast atom bombardment) Thermo Finnigan Mat 95 (70 eV). Elemental analyses were carried out at the Microanalytical Center, Cairo University, Egypt. TLC was performed on analytical Merck 9385 silica aluminum sheets (Kieselgel 60) with Pf_254_ indicator; TLC's were viewed at *λ*_max_ = 254 nm.

##### 3.1.1.1. Synthesis of 2-chloro-*N*-(1,3-dimethyl-2,6-dioxo-1,2,3,6-tetrahydropyrimidin-4-yl)acetamid (3)

6-Amino-1,3-dimethylpyrimidine-2,4(1*H*,3*H*)-dione (1, 1.55 g, 10 mmol) was dissolved in DMF (15 mL), and the solution was cooled to 0 °C. To this cold solution, chloroacetyl chloride (2, 1.56 mL, 20 mmol) in dioxane (5 mL) was added dropwise during a period of 30 min, with vigorous stirring at 0–10 °C. The obtained product 3 was then recrystallized from ethanol as a white precipitate.

###### 3.1.1.1.1 2-Chloro-*N*-(1,3-dimethyl-2,6-dioxo-1,2,3,6-tetrahydropyrimidin-4-yl)acetamide (3)

Yield: 78%; mp 186–188 °C, IR (KBr) *υ*_max_/cm^−1^ = 3242 (NH), 3014 (aliphatic-CH), 1793 (CO), 1643 (CO), 1629 (CO), 1610 (C=C), 1530, 1536 (CH_3_). ^1^H NMR (400 MHz, DMSO-*d*_6_): *δ*_H_ = 3.13 (s, 3H, NCH_3_), 3.29 (s, 3H, NCH_3_), 4.90 (s, 2H, CH_2_), 8.42 (s, 1H, CH-5), 10.78 (bs, 1H, NH). ^13^C NMR (100 MHz, DMSO-*d*_6_): *δ*_C_ = 27.6 (CH_3_), 29.7 (CH_3_), 51.2 (CH_2_), 89.3 (C-5), 149.5 (C-6), 158.2 (^2^C=O), 161.3 (^4^C=O), 189.0 (C=O). MS: *m*/*z* 234 (M^+3^, 10), 232 (M^+1^, 52), 231 (M^+^, 5), 230 (M^−1^, 36), 200 (10), 184 (5), 171 (40), 134 (28), 102 (22), 77 (18). Anal. Calcd for C_8_H_10_ClN_3_O_3_ (231.64): C, 50.37; H, 4.35; N, 18.14. Found: C, 50.47; H, 4.39; N, 18.25.

##### 3.1.1.2. Synthesis of 2-azido-*N*-(1,3-dimethyl-2,6-dioxo-1,2,3,6-tetrahydropyrimidin-4-yl)acetamide (4)

A mixture of 2-chloro-*N*-(1,3-dimethyl-2,6-dioxo-1,2,3,6-tetrahydropyrimidin-4-yl)acetamide (3, 2.31 g, 10 mmol) and sodium azide (1.95 g, 30 mmol) was stirred in DMF at 90 °C for 2 h. The cold reaction mixture was then poured in ice-cold water. The pink solid that separated out was filtered and recrystallized from ethanol.

###### 3.1.1.2.1 2-Azido-*N*-(1,3-dimethyl-2,6-dioxo-1,2,3,6-tetrahydropyrimidin-4-yl)acetamide (4)

Yield: 75%; mp 153–155 °C, IR (KBr) *υ*_max_/cm^−1^ = 3240 (NH), 3157 (olefinic-CH), 2110 (N_3_), 2947 (aliphatic-CH), 1708 (CO), 1643 (CO), 1630 (CO), 1591 (C=C), 1530, 1536 (CH_3_). ^1^H NMR (400 MHz, DMSO-*d*_6_): *δ*_H_ = 3.12 (s, 3H, NCH_3_), 3.28 (s, 3H, NCH_3_), 4.52 (s, 2H, CH_2_), 8.42 (s, 1H, CH-5), 10.76 (bs, 1H, NH). ^13^C NMR (100 MHz, DMSO-*d*_6_): *δ*_C_ = 27.5 (CH_3_), 29.6 (CH_3_), 51.6 (CH_2_), 89.0 (C-5), 149.5 (C-6), 158.1 (^2^C=O), 161.2 (^4^C=O), 192.2 (C=O). MS: *m*/*z* 238 (M^+^, 28), 197 (5), 153 (100), 139 (30), 127 (70), 115 (30), 77 (90), 63 (94). Anal. Calcd for C_8_H_10_N_6_O_3_ (238.20): C, 40.34; H, 4.23; N, 35.28. Found: C, 40.43; H, 4.27; N, 35.30.

##### 3.1.1.3. General procedure for the synthesis of compounds 6a and 6b

A mixture of alkynes 5a,b (ref. 29 and 30) (1 mmol) in 10 mL DMF, CuSO_4_·5H_2_O (0.4 mmol) and (0.4 mmol) of sodium ascorbate was stirred for 10 min at room temperature. To the above mixture, azido compound 4 (1.0 mmol) in 10 mL DMF were added dropwise. The reaction mixture was stirred at room temperature for 20 h. The reaction mixtures were monitored with TLC. After completion, the mixture was diluted with 100 gm ice, and the formed precipitate was filtered off and washed four times with cold water to give compounds 6a,b in good yields.

###### 3.1.1.3.1 2-(4-((Benzo[*d*]oxazol-2-ylthio)methyl)-1*H*-1,2,3-triazol-1-yl)-*N*-(1,3-dimethyl-2,6-dioxo-1,2,3,6-tetrahydropyrimidin-4-yl)acetamide (6a)

Yield: 76%; mp 265–267 °C, IR (KBr) *υ*_max_/cm^−1^ = 3140 (NH), 3000 (aliphatic-CH), 1708 (CO), 1643 (CO), 1629 (CO), 1591 (C=C), 1530, 1536 (CH_3_). ^1^H NMR (400 MHz, DMSO-*d*_6_): *δ*_H_ = 3.18 (s, 3H, NCH_3_), 3.31 (s, 3H, NCH_3_), 4.71 (s, 2H, SCH_2_), 5.76 (s, 2H, NCH_2_), 7.31–7.35 (m, 2H, Ar–H), 7.65–7.67 (m, 2H, Ar–H), 8.06 (bs, 1H, CH-5), 8.42 (s, 1H, CH-5_triazol_), 10.58 (bs, 1H, NH). ^13^C NMR (100 MHz, DMSO-*d*_6_): *δ*_C_ = 26.6 (NCH_3_), 27.6 (NCH_3_), 29.7 (SCH_2_), 58.4 (NCH_2_), 89.0 (C-5),110.2, 118.4, 124.3, 124.6 (CH-Ar), 125.7 (C-5_triazol_), 141.3 (C-4_triazol_), 149.5, 151.4 (C–Ar), 158.1 (C-6), 161.5 (^2^C=O), 163.6 (^4^C=O), 189.4 (C=O). MS: *m*/*z* 427 (M^+^, 28), 289 (100), 166 (20), 151 (15), 157 (100), 129 (20), 91 (82). Anal. Calcd for C_18_H_17_N_7_O_4_S (427.44): C, 50.58; H, 4.01; N, 22.94. Found: C, 50.67; H, 4.05; N, 23.01.

###### 3.1.1.3.2 2-(4-(((1*H*-Benzo[*d*]imidazole-2-yl)thio)methyl)-1*H*-1,2,3-triazol-1-yl)-*N*-(1,3-dimethyl-2,6-dioxo-1,2,3,6-tetrahydropyrimidin-4-yl)acetamide (6b)

Yield: 77%; mp 250–252 °C, IR (KBr) *υ*_max_/cm^−1^ = 3220 (NH), 3180 (Ar-CH), 2950 (aliphatic-CH), 1720 (CO), 1711 (CO), 1620 (CO), 1613 (C=C), 1530, 1535 (CH_3_). ^1^H NMR (400 MHz, DMSO-*d*_6_): *δ*_H_ = 3.17 (s, 3H, NCH_3_), 3.31 (s, 3H, NCH_3_), 4.70 (s, 2H, SCH_2_), 5.76 (s, 2H, NCH_2_), 7.08 (bs, 1H, NH_imidazol_), 7.44–7.57 (m, 2H, Ar–H), 7.89–7.94 (m, 2H, Ar–H), 8.06 (bs, 1H, CH-5), 8.42 (s, 1H, CH-5_triazol_), 10.58 (bs, 1H, NH), 12.45 (bs, 1H, NH). ^13^C NMR (100 MHz, DMSO-*d*_6_): *δ*_C_ = 27.6 (NCH_3_), 29.7 (NCH_3_), 35.8 (SCH_2_), 58.6 (NCH_2_), 88.0 (C-5), 114.2, 117.9, 124.9, 125.0 (CH-Ar), 138.3, 138.3 (C–Ar), 125.4 (C-5_triazol_), 139.8 (C-4_triazol_), 149.5 (C-6), 158.1 (^2^C=O), 161.5 (^4^C=O), 189.4 (C=O). MS: *m*/*z* 426 (M^+^, 38), 365 (5), 278 (5), 182 (40), 154 (100), 118 (100), 82 (42). Anal. Calcd for C_18_H_18_N_8_O_3_S (426.45): C, 50.70; H, 4.25; N, 26.28. Found: C, 50.80; H, 4.28; N, 26.39.

##### 3.1.1.4. General procedure for the synthesis of 1,2,3-triazoles 8a–c

To a solution of 2-azido-*N*-(1,3-dimethyl-2,6-dioxo-1,2,3,6-tetrahydropyrimidin-4-yl)acetamide (4) (1.0 mmol) in dry acetone (30 mL) was added acetylenedicarboxylates 7a,b and/or ethyl propiolate 7c (10 mmol). The reaction mixture was refluxed for 10 h monitored by tlc for completion and cooled. The obtained white precipitate was filtered off and washed with ethanol to give compounds 8a–c.

###### 3.1.1.4.1 Dimethyl 1-(2-((1,3-dimethyl-2,6-dioxo-1,2,3,6-tetrahydropyrimidin-4-yl)amino)-2-oxoethyl)-1*H*-1,2,3-triazole-4,5-dicarboxylate (8a)

Yield: 80%; mp 245–247 °C, IR (KBr) *υ*_max_/cm^−1^ = 3335 (NH), 3165 (olefinic-CH), 2960 (aliphatic-CH), 1736 (CO), 1708 (CO), 1693 (CO), 1642 (CO), 1614 (CO), 1562 (C=C), 1535, 1502 (CH_3_). ^1^H NMR (400 MHz, DMSO-*d*_6_): *δ*_H_ = 3.20 (s, 3H, NCH_3_), 3.32 (s, 3H, NCH_3_), 3.82 (s, 3H, OCH_3_), 3.89 (s, 3H, OCH_3_), 6.04 (s, 2H, CH_2_), 8.49 (s, 1H, CH-5), 10.45 (bs, 1H, NH). ^13^C NMR (100 MHz, DMSO-*d*_6_): *δ*_C_ = 27.6 (NCH_3_), 29.7 (NCH_3_), 52.6 (OCH_3_), 53.2 (OCH_3_), 59.3 (CH_2_), 88.9 (C-5), 130.4 (C-4_triazol_), 139.4 (C-5_triazol_), 149.5 (C-6), 158.1 (^2^C=O, CO_2_Me), 160.6 (CO_2_Me), 161.7 (^4^C=O), 187.7 (C=O). MS: *m*/*z* 380 (M^+^, 40), 264 (5), 195 (100), 166 (90), 140 (66), 82 (100). Anal. Calcd for C_14_H_16_N_6_O_7_ (380.31):C, 44.21; H, 4.24; N, 22.10. Found: C, 44.30; H, 4.28; N, 22.20.

###### 3.1.1.4.2 Diethyl 1-(2-((1,3-dimethyl-2,6-dioxo-1,2,3,6-tetrahydropyrimidin-4-yl)amino)-2-oxoethyl)-1*H*-1,2,3-triazole-4,5-dicarboxylate (8b)

Yield: 79%; mp 165–167 °C, IR (KBr) *υ*_max_/cm^−1^ = 3340 (NH), 3160 (olefinic-CH), 2984 (aliphatic-CH), 1728 (CO), 1710 (CO), 1693 (CO), 1645 (CO), 1615 (CO), 1568 (C=C), 1535, 1510 (CH_3_). ^1^H NMR (400 MHz, DMSO-*d*_6_): *δ*_H_ = 1.18–1.22 (t, 3H, CH_3_), 1.28–1.32 (t, 3H, CH_3_), 3.19 (s, 3H, NCH_3_), 3.28 (s, 3H, NCH_3_), 4.24–4.29 (q, 2H, OCH_2_), 4.34–4.37 (q, 2H, OCH_2_), 5.99 (s, 2H, CH_2_), 8.48 (s, 1H, CH-5), 10.45 (bs, 1H, NH). ^13^C NMR (100 MHz, DMSO-*d*_6_): *δ*_C_ = 13.6 (CH_3_), 13.9 (CH_3_), 27.6 (NCH_3_), 29.7 (NCH_3_), 59.3 (CH_2_), 61.5 (OCH_2_), 62.2 (OCH_2_), 88.9 (C-5), 130.2 (C-4_triazol_), 139.7 (C-5_triazol_), 149.5 (C-6), 157.5 (^2^C=O), 158.1 (^4^C=O), 160.3 (CO_2_Et), 161.6 (CO_2_Et), 187.8 (C=O). MS: *m*/*z* 408 (M^+^, 24), 316 (5), 235 (5), 195 (42), 182 (78), 151 (100), 63 (10). Anal. Calcd for C_16_H_20_N_6_O_7_ (408.37): C, 47.06; H, 4.94; N, 20.58. Found: C, 47.15; H, 4.98; N, 20.69.

###### 3.1.1.4.3 Ethyl 1-(2-((1,3-dimethyl-2,6-dioxo-1,2,3,6-tetrahydropyrimidin-4-yl)amino)-2-oxoethyl)-1*H*-1,2,3-triazole-4-carboxylate (8c)

Yield: 80%; mp 274–276 °C, IR (KBr) *υ*_max_/cm^−1^ = 3240 (NH), 3150 (olefinic-CH), 2982 (aliphatic-CH), 1731 (CO), 1719 (CO), 1658 (CO), 1658 (CO), 1613 (C=C), 1529, 1530 (CH_3_). ^1^H NMR (400 MHz, DMSO-*d*_6_): *δ*_H_ = 1.31 (bt, 3H, CH_3_), 3.21 (s, 3H, NCH_3_), 3.34 (s, 3H, NCH_3_), 4.33 (bq, *J* = 6.3 Hz, 2H, OCH_2_), 5.87 (s, 2H, CH_2_), 8.59 (bs, 1H, CH-5), 8.65 (s, 1H, CH-5_triazol_), 10.57 (bs, 1H, NH),. ^13^C NMR (100 MHz, DMSO-*d*_6_): *δ*_C_ = 14.2 (CH_3_), 27.6 (NCH_3_), 29.7 (NCH_3_), 58.6 (CH_2_), 60.4 (OCH_2_), 89.0 (C-5), 131.0 (C-4_triazol_), 138.5 (C-5_triazol_), 149.5 (C-6), 158.1 (^2^C=O), 160.4 (^4^C=O), 161.6 (CO_2_Et), 188.70 (CO). MS: *m*/*z* 336.26 (M^+^, 24), 308 (10), 255 (10), 235 (10), 195 (18), 155 (20), 81 (20). Anal. Calcd for C_13_H_16_N_6_O_5_ (336.30): C, 46.43; H, 4.80; N, 24.99. Found: C, 46.51; H, 4.84; N, 25.05.

##### 3.1.1.5. General procedure for the synthesis of 1,2,3-triazoles 10a–c

In 20 mL of ethanol, a mixture of azido compound 4 (1.0 mmol) with active methylene ketones 9a–c (1.0 mmol), and triethylamine Et_3_N (1.0 mmol) was heated at 50 °C for 12–15 h, compounds 10a–c were obtained after the reaction was completed by filtering the resulted precipitate after cooling.

###### 3.1.1.5.1 2-(4-Acetyl-5-methyl-1*H*-1,2,3-triazol-1-yl)-*N*-(1,3-dimethyl-2,6-dioxo-1,2,3,6-tetrahydro-pyrimidin-4-yl)acetamide (10a)

Yield: 70%; mp > 340–342 °C, IR (KBr) *υ*_max_/cm^−1^ = 3400 (NH), 3185 (olefinic-CH), 3054 (aliphatic-CH), 1720 (CO), 1710 (CO), 1693 (CO), 1645 (CO), 1620 (CO), 1580 (C=C), 1529 (CH_3_). ^1^H NMR (400 MHz, DMSO-*d*_6_): *δ*_H_ = 2.26 (s, 3H, CH_3_), 3.10 (s, 3H, NCH_3_), 3.30 (s, 3H, COCH_3_), 3.34 (s, 3H, NCH_3_), 5.79 (s, 2H, CH_2_), 8.00 (s, 1H, CH-5_triazol_). 10.50 (bs, 1H, NH). ^13^C NMR (100 MHz, DMSO-*d*_6_): *δ*_C_ = 10.0 (CH_3_), 26.9 (COCH_3_), 27.7 (NCH_3_), 29.7 (NCH_3_), 59.5 (NCH_2_), 61.5 (OCH_2_), 89.0 (C-5), 130.4 (C-4_triazol_), 139.4 (C-5_triazol_), 149.6 (C-6), 157.1 (^2^C=O), 161.3 (^4^C=O), 188.2 (CO), 194.3 (COCH_3_). MS: *m*/*z* 320.26 (M^+^, 40), 288.73 (53), 193.56 (49), 149.35 (32), 112.96 (51), 85.36 (74), 78 (34). Anal. Calcd for C_13_H_16_N_6_O_4_ (320.30):C, 48.75; H, 5.03; N, 26.24. Found: C, 48.86; H, 5.07; N, 26.37.

###### 3.1.1.5.2 2-(4-Acetyl-5-ethoxy-1*H*-1,2,3-triazol-1-yl)-*N*-(1,3-dimethyl-2,6-dioxo-1,2,3,6-tetrahydro-pyrimidin-4-yl)acetamide (10b)

Yield: 75%; mp = 330–332 °C, IR (KBr) *υ*_max_/cm^−1^ = 3350 (NH), 3185 (olefinic-CH), 2975 (aliphatic-CH), 1735 (CO), 1710 (CO), 1674 (CO), 1645 (CO), 1592 (C=C), 1524, 1507 (CH_3_). ^1^H NMR (400 MHz, DMSO-*d*_6_): *δ*_H_ = 1.22–1.25 (t, *J* = 6.3 Hz, 3H, CH_3_), 3.20 (s, 3H, NCH_3_), 3.31 (s, 3H, COCH_3_), 3.34 (s, 3H, NCH_3_), 4.22 (q, *J* = 6.3 Hz, 2H, CH_2_), 5.77 (s, 2H, CH_2_), 8.10 (s, 1H, CH-5_triazol_). 10.50 (bs, 1H, NH). ^13^C NMR (100 MHz, DMSO-*d*_6_): *δ*_C_ = 13.9 (CH_3_), 27.6 (COCH_3_), 27.9 (NCH_3_), 28.4 (NCH_3_), 59.4 (CH_2_), 88.0 (C-5), 131.3 (C-4_triazol_), 140.5 (C-5_triazol_), 149.5 (C-6), 159.5 (^2^C=O), 164.1 (^4^C=O), 187.2 (CO), 194.2 (COCH_3_). Anal. Calcd for C_14_H_18_N_6_O_5_ (350.33): C, 48.00; H, 5.18; N, 23.99. Found: C, 48.10; H, 5.22; N, 24.08.

###### 3.1.1.5.3 2-(4-Benzoyl-5-ethoxy-1*H*-1,2,3-triazol-1-yl)-*N*-(1,3-dimethyl-2,6-dioxo-1,2,3,6-tetrahydropyrimidin-4-yl)acetamide (10c)

Yield: 75%; mp 323–325 °C, IR (KBr) *υ*_max_/cm^−1^ = 3370 (NH), 3165 (olefinic-CH), 2970 (aliphatic-CH), 1721 (CO), 1700 (CO), 1685 (CO), 1640 (CO), 1600 (C=C), 1520, 1502 (CH_3_). ^1^H NMR (400 MHz, DMSO-*d*_6_): *δ*_H_ = 1.23–1.28 (t, *J* = 6.3 Hz, 3H, CH_3_), 3.21 (s, 3H, NCH_3_), 3.30 (s, 3H, COCH_3_), 3.34 (s, 3H, NCH_3_), 4.12 (q, *J* = 6.3 Hz, 2H, CH_2_), 5.78 (s, 2H, CH_2_), 7.26–7.40 (m, 6H, Ar–H), 8.32 (s, 1H, CH-5_triazol_). 10.50 (bs, 1H, NH). ^13^C NMR (100 MHz, DMSO-*d*_6_): *δ*_C_ = 13.9 (CH_3_), 27.6 (COCH_3_), 27.7 (NCH_3_), 27.9 (NCH_3_), 59.4 (CH_2_), 88.0 (C-5), 131.3 (C-4_triazol_), 140.5 (C-5_triazol_), 149.5 (C-6), 159.5 (^2^C=O), 164.1 (^4^C=O), 187.2 (CO), 194.0 (COCH_3_). MS: *m*/*z* 412.73 (M^+^, 36), 411.90 (46), 387.33 (34), 339.36 (48), 289.62 (100), 276.06 (30), 209.91 (50), 121.16 (54). Anal. Calcd for C_19_H_20_N_6_O_5_ (412.40): C, 55.34; H, 4.89; N, 20.38. Found: C, 55.46; H, 4.92; N, 20.46.

### 3.2. Biology

#### 3.2.1. *In vitro* cytotoxicity evaluation (MTT assay)

The cytotoxic activity of the twelve synthesized uracil derivatives was assessed against A549 (lung adenocarcinoma), MCF-7 (breast adenocarcinoma), PANC-1 (pancreatic carcinoma), and HT-29 (colorectal adenocarcinoma) cell lines, alongside the non-tumorigenic Vero cell line as a normal cell reference, using the MTT colorimetric assay, with doxorubicin serving as the positive control. Cells were seeded at 1 × 10^5^ cells per mL (100 µL per well) in 96-well plates and incubated at 37 °C/5% CO_2_ for 24 h to establish confluent monolayers. Following medium removal and washing, serial two-fold dilutions of each compound in RPMI-1640/2% FBS were added in triplicate for 48 h. MTT solution (5 mg mL^−1^ in PBS; BioBasic Canada Inc., 20 µL per well) was then added, and after 4 h of incubation, the formazan crystals were dissolved in DMSO (200 µL) and absorbance was recorded at 560 nm (ref. 620 nm). Cell viability was expressed as a percentage relative to untreated controls, and IC_50_ values were derived from dose–response curves using [GraphPad Prism. 8]. Morphological examination was performed in parallel to corroborate quantitative findings, with apoptosis identified by nuclear condensation and fragmentation, and necrosis by nuclear swelling and chromatin flocculation.^42–44^

#### 3.2.2. *In vitro* EGFR kinase inhibition assay

The inhibitory activity of the synthesized uracil derivatives against wild-type EGFR kinase was evaluated using the EGFR Kinase Assay Kit (BPS Bioscience, Cat. #40321) in a luminescence-based 96-well format, with erlotinib as the positive control reference inhibitor. Reaction mixtures (50 µL per well) were assembled by combining 5× kinase assay buffer (6 µL), ATP (500 µM, 1 µL), PTK substrate poly(Glu : Tyr 4 : 1) (50×, 1 µL), and water (17 µL), followed by the addition of 5 µL of each test compound solution. Reactions were initiated by the addition of recombinant EGFR (wild-type, 1 ng µL^−1^, 20 µL) and incubated at 30 °C for 40 min. Blank wells received 1× kinase assay buffer in place of enzyme. Kinase-Glo MAX luminescence reagent (Promega, 50 µL per well) was then added, and after 15 min at room temperature in the dark, luminescence was recorded using a microplate reader. Residual kinase activity was calculated relative to the enzyme-only control, and IC_50_ values were derived from dose–response curves using [GraphPad Prism.8]. All samples were tested in duplicate.^45^

#### 3.2.3. *In vitro* CDK1/CyclinB1 kinase inhibition assay

The inhibitory activity of the synthesized uracil derivatives against CDK1/CyclinB1 was evaluated using the CDK1 Assay Kit (BPS Bioscience, Cat. #79597) in a luminescence-based 96-well format, with roscovitine serving as the positive control reference inhibitor. Reaction mixtures (50 µL per well) were assembled by combining 5× kinase assay buffer 1 (6 µL), ATP (500 µM, 1 µL), 10× CDK substrate peptide 1 (5 µL), and distilled water (13 µL), followed by the addition of 5 µL of each test compound solution. Reactions were initiated by the addition of recombinant CDK1/CyclinB1 enzyme (∼1.0 ng µL^−1^, 20 µL) and incubated at 30 °C for 45 min. Blank wells received 1× kinase assay buffer in place of enzyme, and blank values were subtracted from all readings. Kinase-Glo MAX luminescence reagent (Promega, 50 µL per well) was then added, and after 15 min at room temperature in the dark, luminescence was recorded using a microplate reader set to luminescence mode. Residual CDK1 activity was calculated relative to the enzyme-only control, and IC_50_ values were derived from dose–response curves using [GraphPad Prism.8]. All samples were tested in duplicate.

#### 3.2.4. Quantification of apoptotic markers by ELISA (Bax, Bcl-2, and Caspase-3)

The intracellular expression levels of the pro-apoptotic proteins Bax and Caspase-3, and the anti-apoptotic protein Bcl-2, were quantified in compound-treated HT-29 and MCF-7 cells using commercially available sandwich ELISA kits, following a common cell preparation protocol. Briefly, cells were seeded at 1 × 10^5^ cells per mL (2 mL/plate) in 3.5 cm tissue culture plates and incubated at 37 °C for 24 h to form confluent monolayers, after which the growth medium was replaced with DMEM supplemented with 2% FBS containing two concentrations of each test compound (IC_50_ and IC_50_/10), with PBS-treated plates serving as controls. After 24 h of compound exposure, cells were washed with ice-cold PBS, detached by trypsinization, pelleted by centrifugation at 1000×*g* for 5 min, washed three times in pre-cooled PBS, resuspended in fresh PBS, and lysed by sonication; cellular debris was removed by centrifugation at 1500×*g* for 10 min and the clarified lysates were assayed immediately. For ELISA quantification, 100 µL of each standard or sample lysate was added to the designated wells of the 96-well plate and incubated for 80 min at 37 °C, followed by three washes with 1× wash solution (200 µL per well). Biotinylated detection antibody (100 µL per well) was then added and incubated for 50 min at 37 °C, followed by three washes, and Streptavidin-HRP working solution (100 µL per well) was subsequently added and incubated for 50 min at 37 °C, followed by five washes. TMB substrate solution (90 µL per well) was added and the plate was incubated for 20 min at 37 °C in the dark, after which the reaction was terminated by the addition of stop reagent (50 µL per well), converting the blue color to yellow. Absorbance was measured at 450 nm using a microplate reader, and protein concentrations (ng mL^−1^) were interpolated from the respective standard calibration curves. Results were expressed as fold-change relative to untreated control cells.

#### 3.2.5. Cell cycle analysis by flow cytometry

HT29 cells were prepared using the COULTER DNA PREP Reagents Kit (PN 6607055) according to the manufacturer's protocol. Cells were first permeabilized with DNA PREP LPR solution (comprising nonionic detergents, saline, and stabilizers, with <0.1% potassium cyanide and <0.1% NaN_3_), and subsequently stained with DNA PREP Stain, consisting of propidium iodide (50 µg mL^−1^) and RNase Type III-A (4 KU per mL). DNA content was measured by flow cytometry using 488 nm excitation, with fluorescence emission collected between 560 and 680 nm, allowing the cell population to be resolved into G0/G1, S, and G2/M phases based on single-parameter DNA histograms. Prior to analysis, the instrument was calibrated with 10 µm Flow-Check Fluorospheres to maintain a half-peak coefficient of variation of ≤2% in the red fluorescence channel. All procedures were carried out at room temperature (20–25 °C), and reagents were stored at 2–8 °C between uses.

#### 3.2.6. Docking study

##### 3.2.6.1. Software and scoring

Docking was performed with MOE (2014.2) in standard-precision (SP) mode, and the *S* score reported in Table 6 is the Glide docking score in kcal mol^−1^. C-Docker energies were calculated and visualized in Discovery Studio Visualizer.

##### 3.2.6.2. Ligand preparation

Compound 10a was drawn in ChemDraw (v22.2.0) and converted to 3D with LigPrep in Maestro (v13.4). Tautomers and ionization states were generated with Epik, and the structure was energy-minimized with the OPLS4 force field.

##### 3.2.6.3. Protein preparation

Crystal structures of EGFR (PDB: 1M17) and CDK1 (PDB: 4YC6) were obtained from the RCSB Protein Data Bank. Each structure was processed with the Protein Preparation Workflow (OPLS4): hydrogens were added and hydrogen-bond networks optimized, water molecules and heteroatoms other than the co-crystallized ligand were removed, and only the chain containing the bound ligand was retained. Restrained minimization was then performed with OPLS4.

##### 3.2.6.4. Receptor grid

Receptor grids were generated with the Glide grid module, centred on the co-crystallized ligand in each structure.

##### 3.2.6.5. Protocol validation

The docking protocol was validated by redocking erlotinib into 1M17 and roscovitine into 4YC6. Heavy-atom RMSDs between the redocked and crystallographic poses were calculated; values below 2.0 Å were considered successful reproduction (Table 6).

##### 3.2.6.6. Ligand docking

Flexible-ligand docking was performed in Glide SP mode, with Epik state penalties added to the docking score and penalties applied to non-cis/trans amide bonds. Partial-charge cutoff and van der Waals scaling for ligand atoms were 0.15 and 0.80, respectively. The top-scoring pose for each compound was retained for interaction analysis, which was performed in Discovery Studio Visualizer. Interactions were classified as hydrogen bonds (donor or acceptor), π–H, or π–π contacts, and distances are reported in Table 6.

##### 3.2.6.7. ADMETlab 3.0

Pharmacokinetic, physicochemical, drug-likeness, and toxicity properties of the most active compounds were predicted using the ADMETlab 3.0 online platform (https://admetlab3.scbdd.com/). Parameters related to absorption, distribution, metabolism, excretion, toxicity, and medicinal chemistry profiles were evaluated to estimate the druglikeness and pharmacokinetic behavior of the investigated compounds.^39,40^

## 4. Conclusion

A series of uracil–1,2,3-triazole hybrids was successfully synthesized in this study *via* three click chemistry routes and were characterized completely by spectroscopic and analytical methods. Biological evaluation revealed that compound 3 is the most-broad antiproliferative agent and compound 10c is the most selective against the HT29 cell line of colon cancer, while compound 10a is the most potent dual EGFR/CDK1 inhibitor in the series, approximately 2.4-fold less potent than erlotinib against EGFR and about 4.8-fold more potent than roscovitine against CDK1. ELISA quantification of apoptosis markers showed that the active hybrids, and in particular 10c, upregulate Caspase-3 and BAX together with a larger rise in Bcl-2, so the apoptotic mechanism remains to be established. The findings show that the target compounds are associated with G0/G1 cell-cycle accumulation and increased apoptosis; these phenotypic observations are consistent with, but do not establish, dual EGFR/CDK1 targeting in cells. Molecular docking predicts that compound 10a can occupy the ATP sites of both EGFR and CDK1, and normal-mode analysis supports the structural plausibility of these complexes; experimental confirmation is required. Additionally, compound 10a shows several predicted pharmacokinetic and safety liabilities, including hepatotoxicity risk, low predicted oral absorption and a short half-life, which must be addressed in optimization. These results collectively suggest that the uracil–triazole hybrid scaffold is a viable chemical platform to develop dual-kinase targeted anticancer therapeutics, as compound 10a is a promising lead compound for further optimization, more comprehensive mechanistic studies, and *in vivo* evaluation.

## Author contributions

A. H. M. (supervision, writing, editing, and revision), A. A. A. (concept, supervision, writing, editing, revision, and submission), S. M. N. A. (writing, editing, and revision), A. A. F. (experimental), A. B. Brown (editing), S. M. G. (supervision, editing, revision). All authors have read and agreed to the published version of the manuscript.

## Conflicts of interest

The authors declare no conflict of interest.

## Supplementary Material

RA-OLF-D6RA09088E-s001

## Data Availability

Supplementary information (SI): all pertinent data, including high-resolution NMR, MS and IR spectra and raw assay data. See DOI: https://doi.org/10.1039/d6ra09088e.

## References

[cit1] Dai J., Tian S., Yang X., Liu Z. (2020). Front. Chem..

[cit2] Shaheen M. A., Darwish K. M., Kishk S. M., El-Sayed M. A.-A., Salama I. (2025). Bioorg. Chem..

[cit3] Upadhyay D. B., Mokariya J. A., Patel P. J., Patel S. G., Parmar M. P., Vala D. P., Ferro F., Rajani D. P., Narayan M., Kumar J., Banerjee S., Patel H. M. (2025). ACS Bio Med Chem Au.

[cit4] Hu Y., Liu Z., Zha G., Long S., Sridhara M. B., Kumar K. S. S., Rakesh K. P. (2023). Process Biochem..

[cit5] Rabee A. R., Abdel-Hamid H., Soliman S. M., Ashraf S., Sobhy A. A., Ghareeb D. A., Saad A. K., Hagar M. (2025). Sci. Rep..

[cit6] Silalai P., Jaipea S., Tocharus J., Athipornchai A., Suksamrarn A., Saeeng R. (2022). ACS Omega.

[cit7] Chahal S., Rani P., Shweta, Goel K. K., Joshi G., Singh R., Kumar P., Singh D., Sindhu J. (2024). Bioorg. Chem..

[cit8] Şahin İ., Özgeriş F. B., Köse M., Bakan E., Tümer F. (2021). J. Mol. Struct..

[cit9] Hawash M. (2024). Cells.

[cit10] Wu J., Chen Y., Li R., Guan Y., Chen M., Yin H., Yang X., Jin M., Huang B., Ding X., Yang J., Wang Z., He Y., Wang Q., Luo J., Wang P., Mao Z., Huen M. S. Y., Lou Z., Yuan J., Gong F. (2024). J. Cell Biol..

[cit11] Mostafa S. M., Aly A. A., Sayed S. M., Raslan M. A., Ahmed A. E., Nafady A., Ishak E. A., Shawky A. M., Abdelhafez E.-S. M. N. (2021). J. Mol. Struct..

[cit12] Al-Hussain S. A., Dawood D. H., Farghaly T. A., Abu Alnjaa A. M., Zaki M. E. A. (2025). Pharmaceuticals.

[cit13] Youssif B. G. M., Morcoss M. M., Bräse S., Abdel-Aziz M., Abdel-Rahman H. M., Abou El-Ella D. A., Abdelhafez E. S. M. N. (2024). Molecules.

[cit14] Othman D. I. A., Hamdi A., Tawfik S. S., Elgazar A. A., Mostafa A. S. (2023). J. Enzyme Inhib. Med. Chem..

[cit15] Hassoub E. M. M., Hussein B. R. M., Mohamed M. F. A., Abdelhafez E.-S. M. N. (2026). Bioorg. Chem..

[cit16] Bass A. K. A., Nageeb E.-S. M., El-Zoghbi M. S., Mohamed M. F. A., Badr M., Abuo-Rahma G. E.-D. A. (2022). Bioorg. Chem..

[cit17] El-Sayed W. A., Alminderej F. M., Mounier M. M., Nossier E. S., Saleh S. M., Kassem A. F. (2022). Molecules.

[cit18] Krakisha F. M., Othman D. I., El Husseiny W. M., Nasr M. N. (2024). Future Med. Chem..

[cit19] Abu Al Rub H. M., Eissa A. G. (2026). Pharmaceuticals.

[cit20] Yang T., Zhu W., Yang J., Zhou F., Yan L., Liu L., Le Y. (2026). Bioorg. Chem..

[cit21] Ahmed A. A. Y., Mohammed A. F., Almarhoon Z. M., Bräse S., Youssif B. G. M. (2025). Front. Chem..

[cit22] Mohamed A. M., Abou-Ghadir O. M. F., Mostafa Y. A., Almarhoon Z. M., Bräse S., Youssif B. G. M. (2024). RSC Adv..

[cit23] Fan Y., Huang Z., Wang X., Ma Y., Li Y., Yang S., Shi Y. (2020). Molecules.

[cit24] Ahmed N. M., Youns M. M., Soltan M. K., Said A. M. (2021). Molecules.

[cit25] Reymova F., Sever B., Topalan E., Sevimli-Gur C., Can M., Tuyun A. F., Başoğlu F., Ece A., Otsuka M., Fujita M., Demirci H., Ciftci H. (2025). Pharmaceuticals.

[cit26] El-Kalyoubi S., El-Sebaey S. A., El-Sayed A. A., Abdelhamid M. S., Agili F., Elfeky S. M. (2023). J. Enzyme Inhib. Med. Chem..

[cit27] Alshammari M. B., Aly A. A., Youssif B. G. M., Bräse S., Ahmad A., Brown A. B., Ibrahim M. A. A., Mohamed A. H. (2022). Front. Chem..

[cit28] Aly A. A., Alshammari M. B., Ahmad A., Gomaa H. A. M., Youssif B. G. M., Bräse S., Ibrahim M. A. A., Mohamed A. H. (2023). Arab. J. Chem..

[cit29] El-Sheref E. M., Ameen M. A., El-Shaieb K. M., Abdel-Latif F. F., Abdel-naser A. I., Brown A. B., Bräse S., Fathy H. M., Ahmad I., Patel H., Gomaa H. A. M., Youssif B. G. M., Mohamed A. H. (2022). Molecules.

[cit30] Kakkar S., Kumar S., Narasimhan B., Lim S. M., Ramasamy K., Mani V., Shah S. A. A. (2018). Chem. Cent. J..

[cit31] Faraji L., Shahkarami S., Nadri H., Moradi A., Saeedi M., Foroumadi A., Ramazani A., Haririan I., Ganjali M. R., Shafiee A., Khoobi M. (2017). J. Chem. Res..

[cit32] Zeghada S., Bentabed-Ababsa G., Derdour A., Abdelmounim S., Domingo L. R., Sáez J. A., Roisnel T., Nassar E., Mongin F. (2011). Org. Biomol. Chem..

[cit33] El-Kalyoubi S. A. , Gomaa H. A. M., Abdelhafez E. M. N., Ramadan M., Agili F. and Youssif B. G. M., 2023, 10.3390/ph16050716 PMC10223936 37242499

[cit34] Abdel-Hady M. Y., Morcoss M. M., Alzahrani A. Y. A., Youssif B. G. M., Abdelhafez E. S. M. N., Abdel-Aziz M. (2025). Arch. Pharm..

[cit35] Ibrahim K. H., Abdelhafez E. M. N., Fawzy M. A. (2025). J. Vet. Med. Res..

[cit36] Alley M. C., Scudiero D. A., Monks A., Hursey M. L., Czerwinski M. J., Fine D. L., Abbott B. J., Mayo J. G., Shoemaker R. H., Boyd M. R. (1988). Cancer Res..

[cit37] Desai N., Mahto M. K., Alekhya B., Naveen C. R., Bhaskar M. (2012). Int. J. Pharm. Sci. Rev. Res..

[cit38] Mashkani Z. S., Yali Z. P., Dorgalaleh A., Shams M. (2023). bioRxiv.

[cit39] Gu Y., Yu Z., Wang Y., Chen L., Lou C., Yang C., Li W., Liu G., Tang Y. (2024). Nucleic Acids Res..

[cit40] Yergaliyeva E., Khamit A., Zakiriyanova G., Kolbaeva Z. (2025). 3i intellect, idea, Innov..

[cit41] Fu L., Shi S., Yi J., Wang N., He Y., Wu Z., Peng J., Deng Y., Wang W., Wu C. (2024). Nucleic Acids Res..

[cit42] Dong J., Wang N.-N., Yao Z.-J., Zhang L., Cheng Y., Ouyang D., Lu A.-P., Cao D.-S. (2018). J. Cheminf..

[cit43] van de Loosdrecht A. A., Beelen R. H. J., Ossenkoppele G. J., Broekhoven M. G., Langenhuijsen M. M. A. C. (1994). J. Immunol. Methods.

[cit44] Slater T. F., Sawyer B., Sträuli U. (1963). Biochim. Biophys. Acta.

[cit45] Li J., Liu X., Li S., Wang Y., Zhou N., Luo C., Luo X., Zheng M., Jiang H., Chen K. (2013). Int. J. Mol. Sci..

