# Supplementary material for: Design, click synthesis, and biological evaluation of novel uracil-1,2,3-triazole hybrids as dual EGFR/CDK1 inhibitors: antiproliferative activity, mechanistic insights, and in silico studies
Source: RSC Adv. 2026 Nov 3. Online ahead of print. doi: 10.1039/d6ra09088e (PMC13730394; doi:10.1039/d6ra09088e)
Supplement: Supplementary file 1 [file RA-OLF-D6RA09088E-s001.pdf]

Supplementary Information (SI) for RSC Advances.
This journal is © The Royal Society of Chemistry

Design and click synthesis, of novel uracil-1,2,3-triazole hybrids as dual EGFR/CDK1 inhibitors: Antiproliferative activity, molecular docking study and molecular dynamics simulation

Asmaa H. Mohamed,[1] Ashraf A. Aly, [1]* El-Shimaa M. N. Abdelhafez,[2]* Aya A. Fouly,[1] Alan B. Brown,[3] Sageda A. Gedamy[1]

[1] Chemistry Department, Faculty of Science, Minia University, 61519 Minia, e-mails: asmaa.hamouda@mu.edu.eg, ashrafaly63@yahoo.com (ashraf.shehata@mu.edu.eg), ayaattia2588@gmail.com, sagedaali332@yahoo.com

[2] Department of Medicinal Chemistry, Faculty of Pharmacy, Minia University, 61519 Minia, Egypt; e-mail: shimaanaguib_80@mu.edu.eg

[3] Department of Chemistry and Chemical Engineering, Florida Institute of Technology, Melbourne, FL 32901, USA. e-mail: abrown@fit.edu

General Remarks: The IR spectra were recorded by ATR technique (ATR = Attenuated Total Reflection) with a FT device (FT-IR Bruker IFS 88), Minia University, Faculty of science, The NMR spectra were measured in DMSO-d_6 on a Bruker AV-400 spectrometer, 400 MHz for ^{1}H, and 100 MHz for ^{13}C; and the chemical shifts are expressed in *d* (ppm), versus internal tetramethylsilane (TMS) = 0 for ^{1}H and ^{13}C, and external liquid ammonia = 0. The description of signals includes: s = singlet, d = doublet, t = triplet, q = quartet, m = multiplet. Mass spectra were recorded on a FAB (fast atom bombardment) Thermo Finnigan Mat 95 (70 eV). Elemental analyses were carried out at the Microanalytical Center, Cairo University, Egypt. TLC was performed on analytical Merck 9385 silica aluminum sheets (Kieselgel 60) with Pf_{254} indicator; TLC's were viewed at l_{max} = 254 nm. All the reactions were carried out in a Milestone START Microwave Labstation (temperature control by IR sensor).

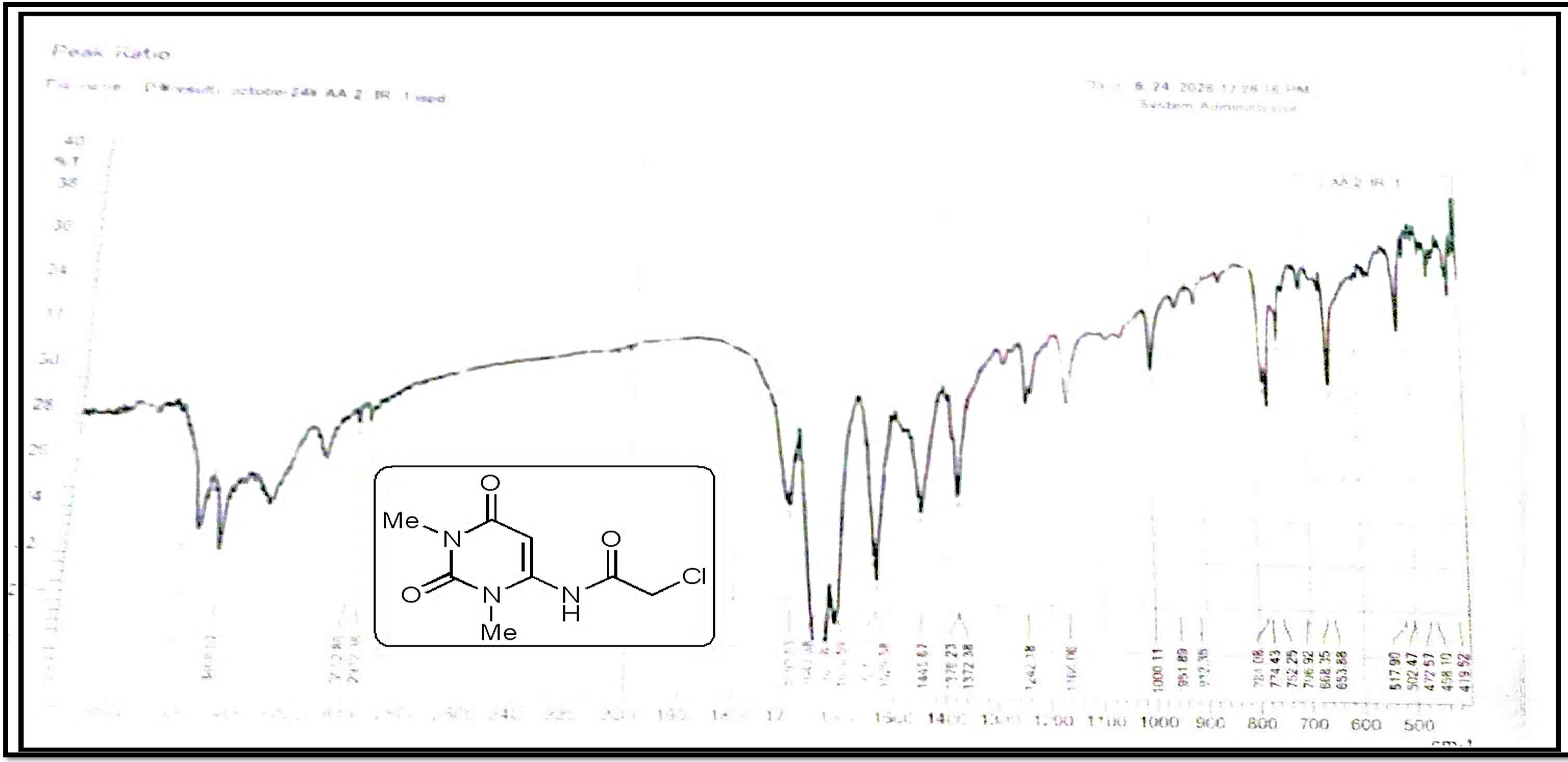

SI figure 1: IR spectrum of compound **3**

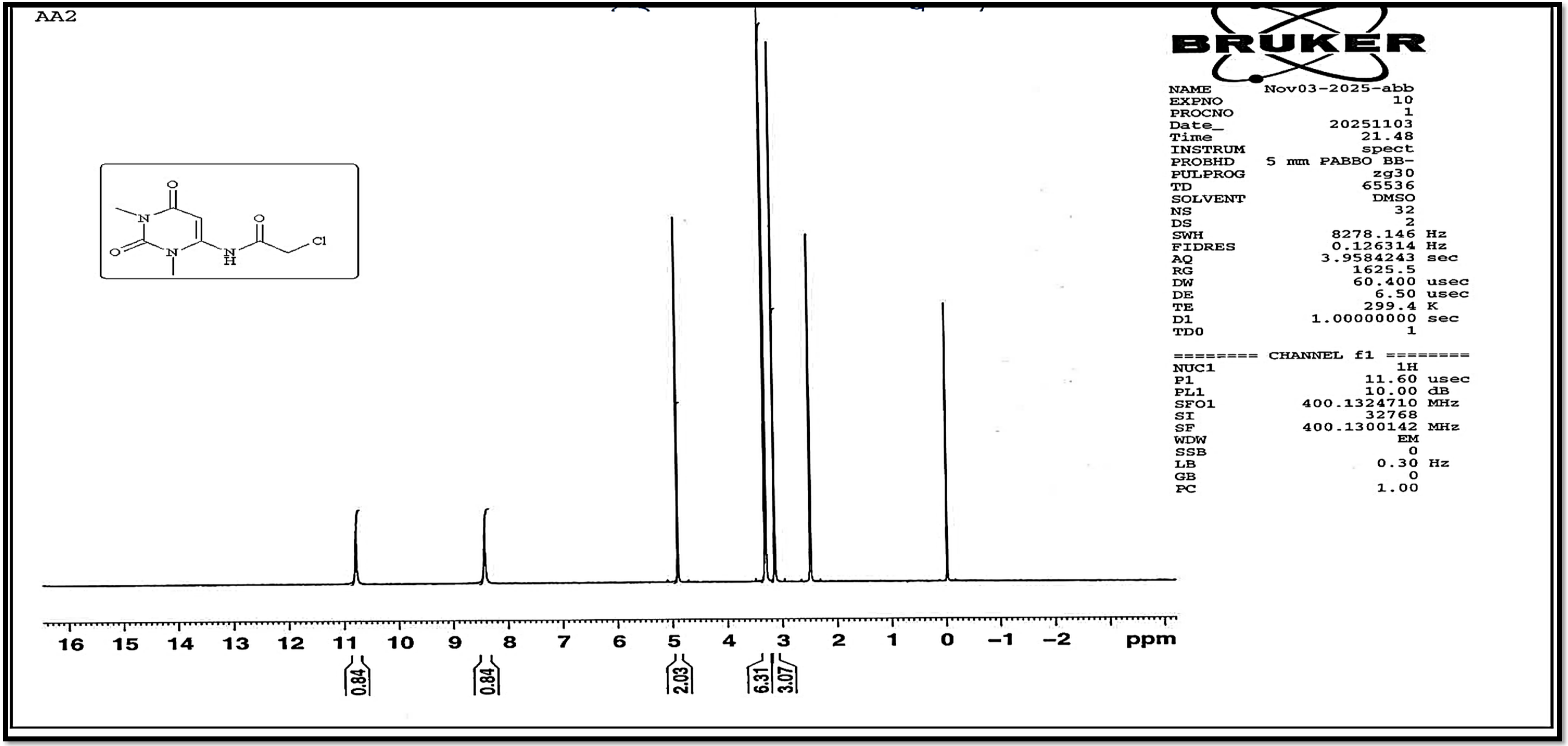

SI figure 2: ^{1}H NMR spectrum of **3**

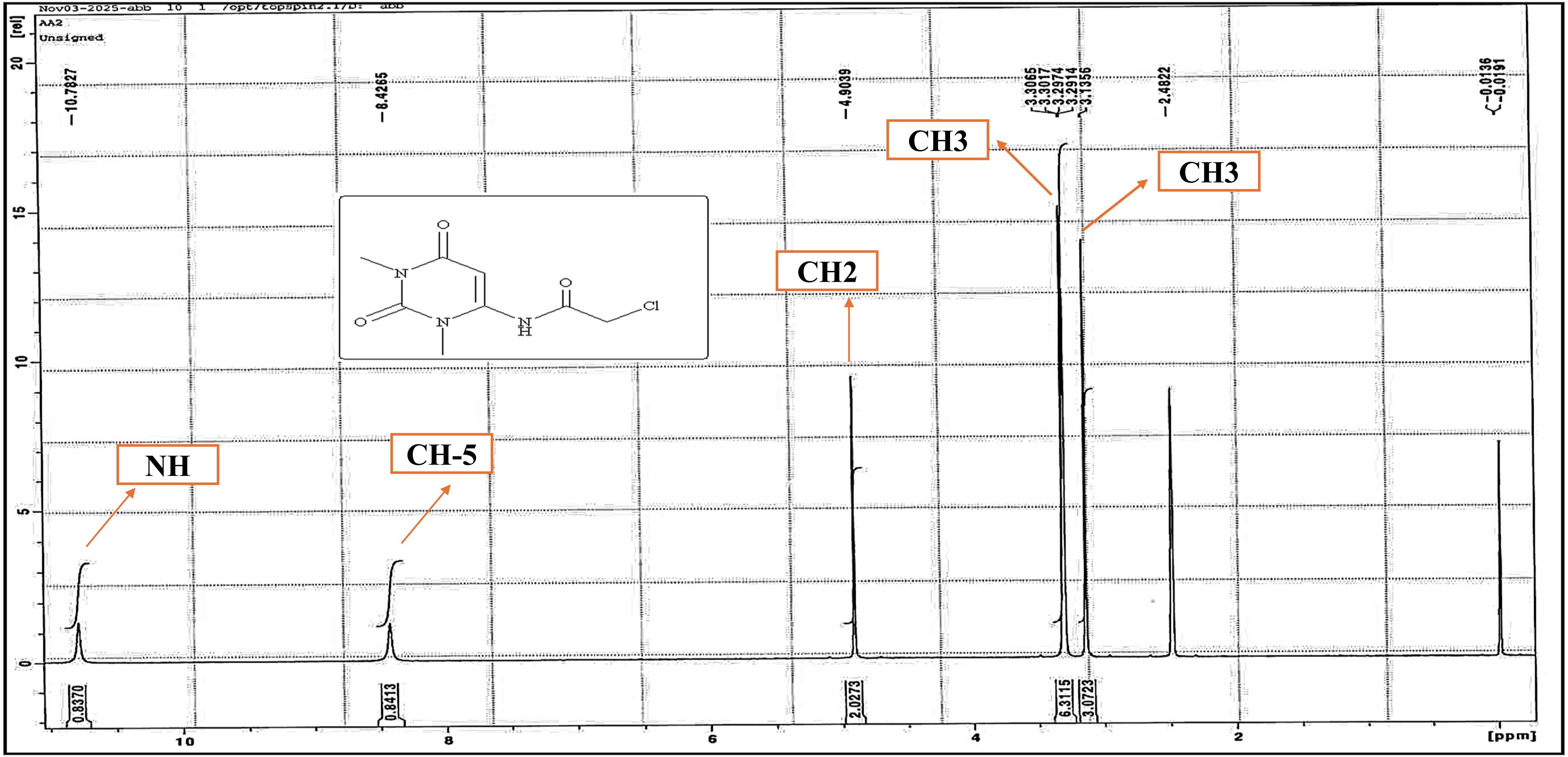

SI figure 3: Expanded ^{1}H NMR spectrum of **3**

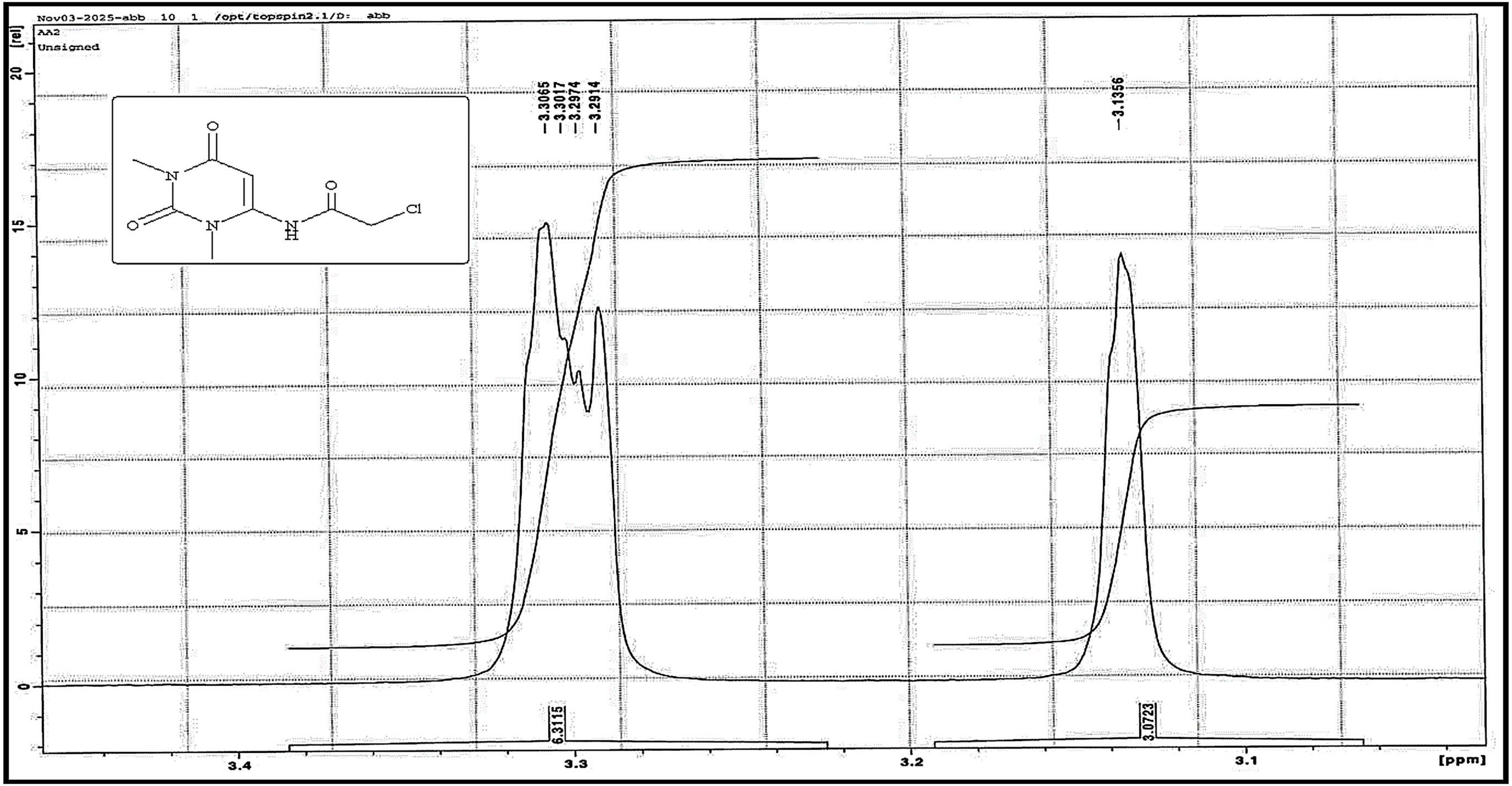

SI figure 4: Expanded ^{1}H NMR spectrum of **3**

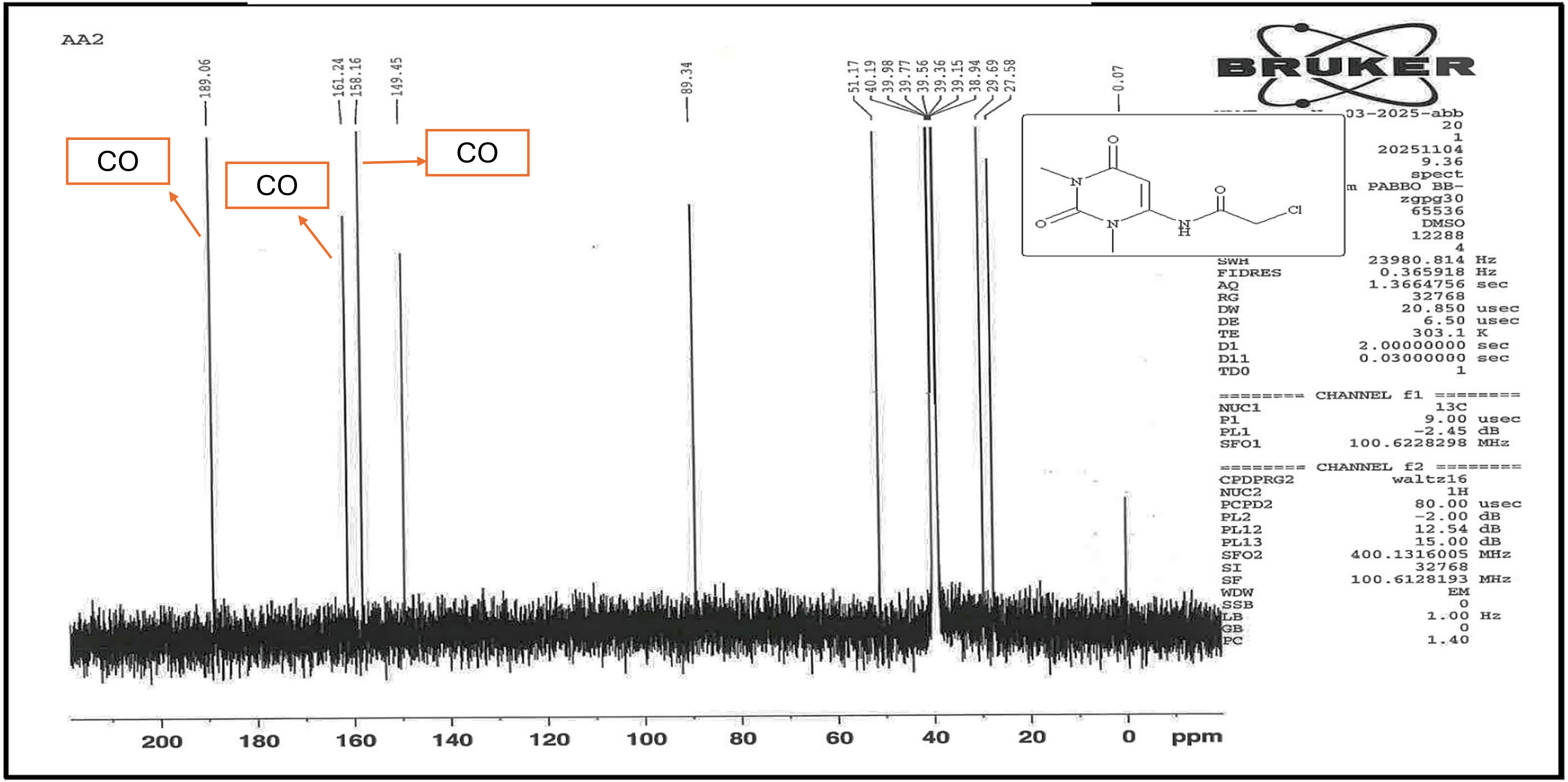

SI figure 5: ^{13}C NMR spectrum of **3**

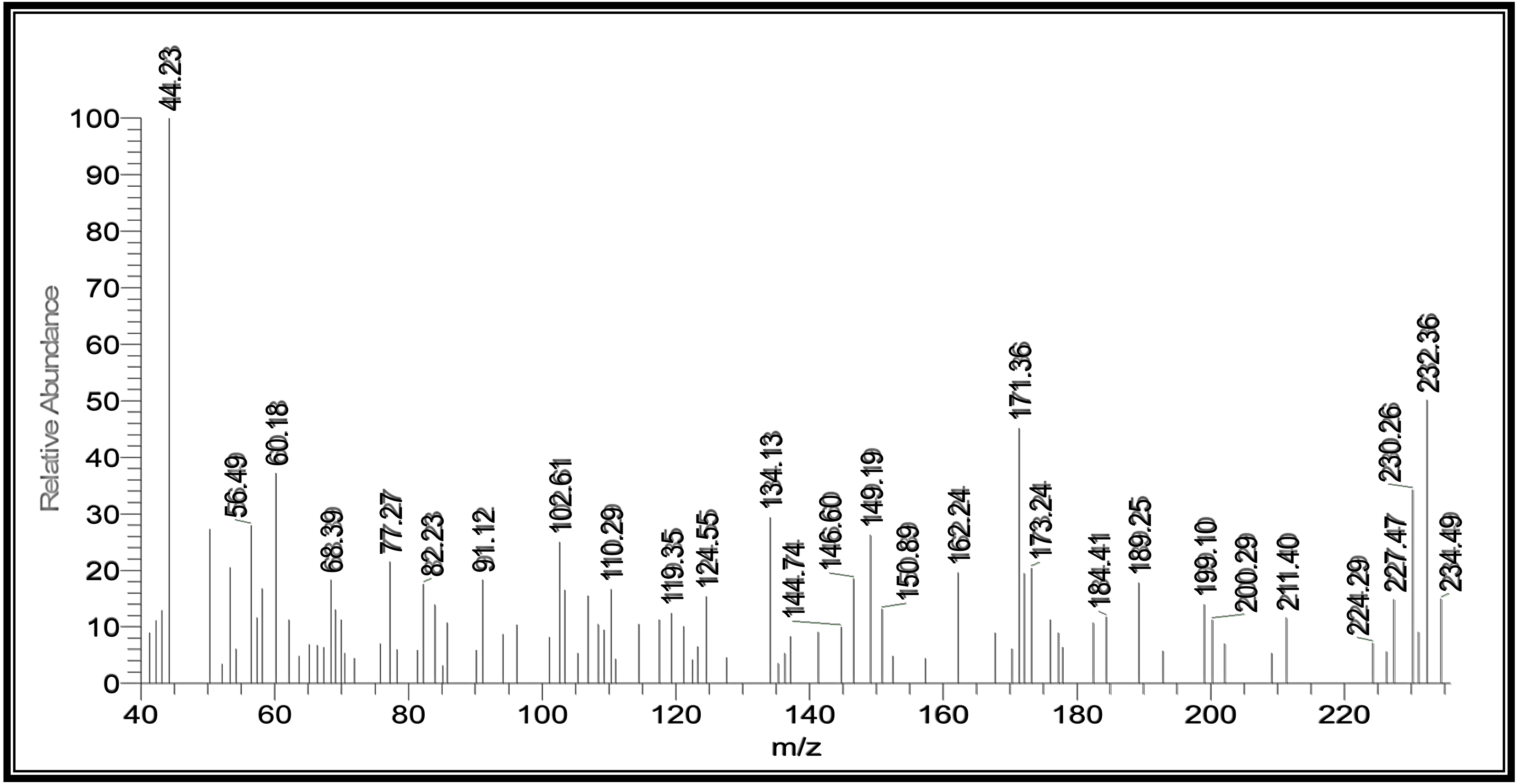

SI figure 6: Mass spectrum of **3**

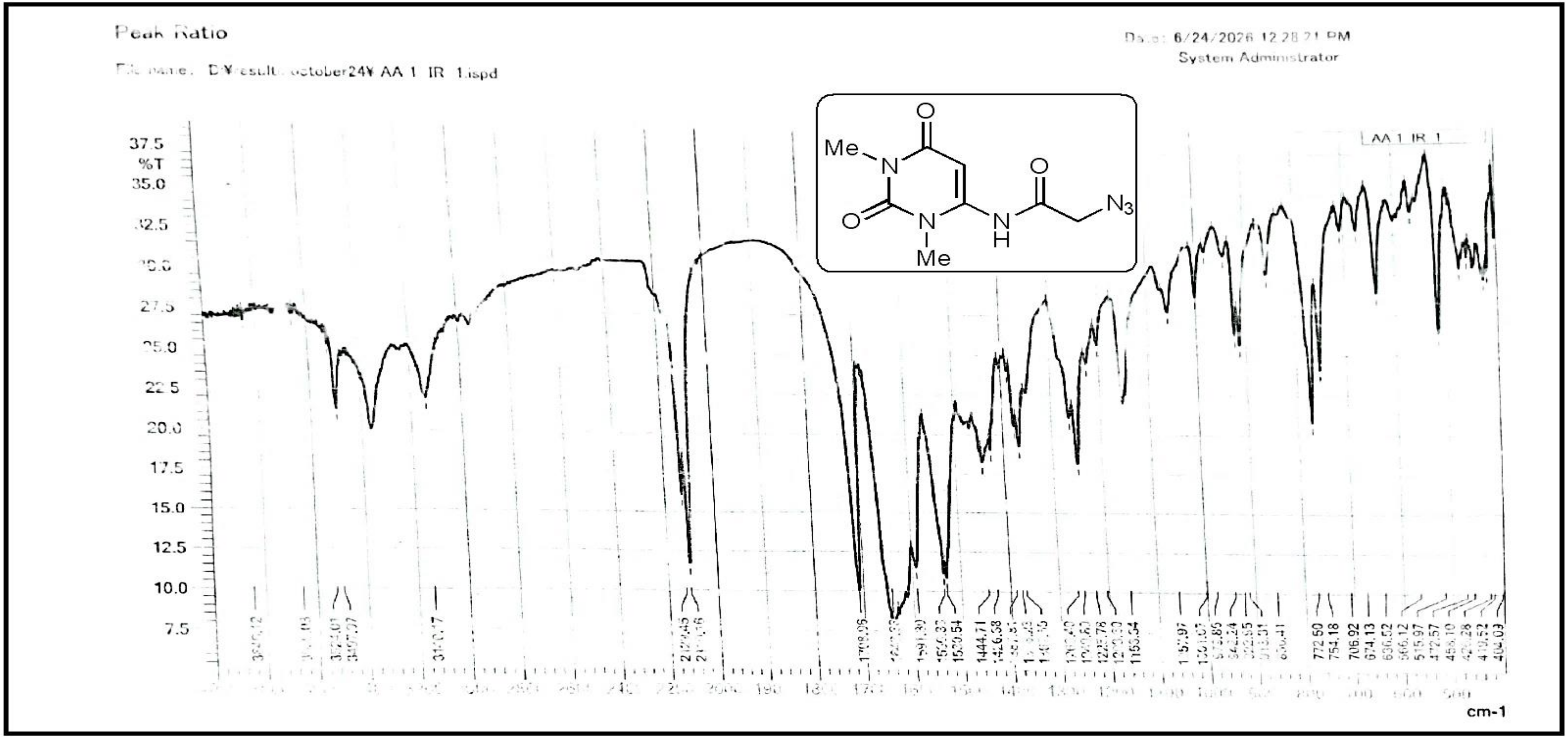

SI figure 7: IR spectrum of compound **4**

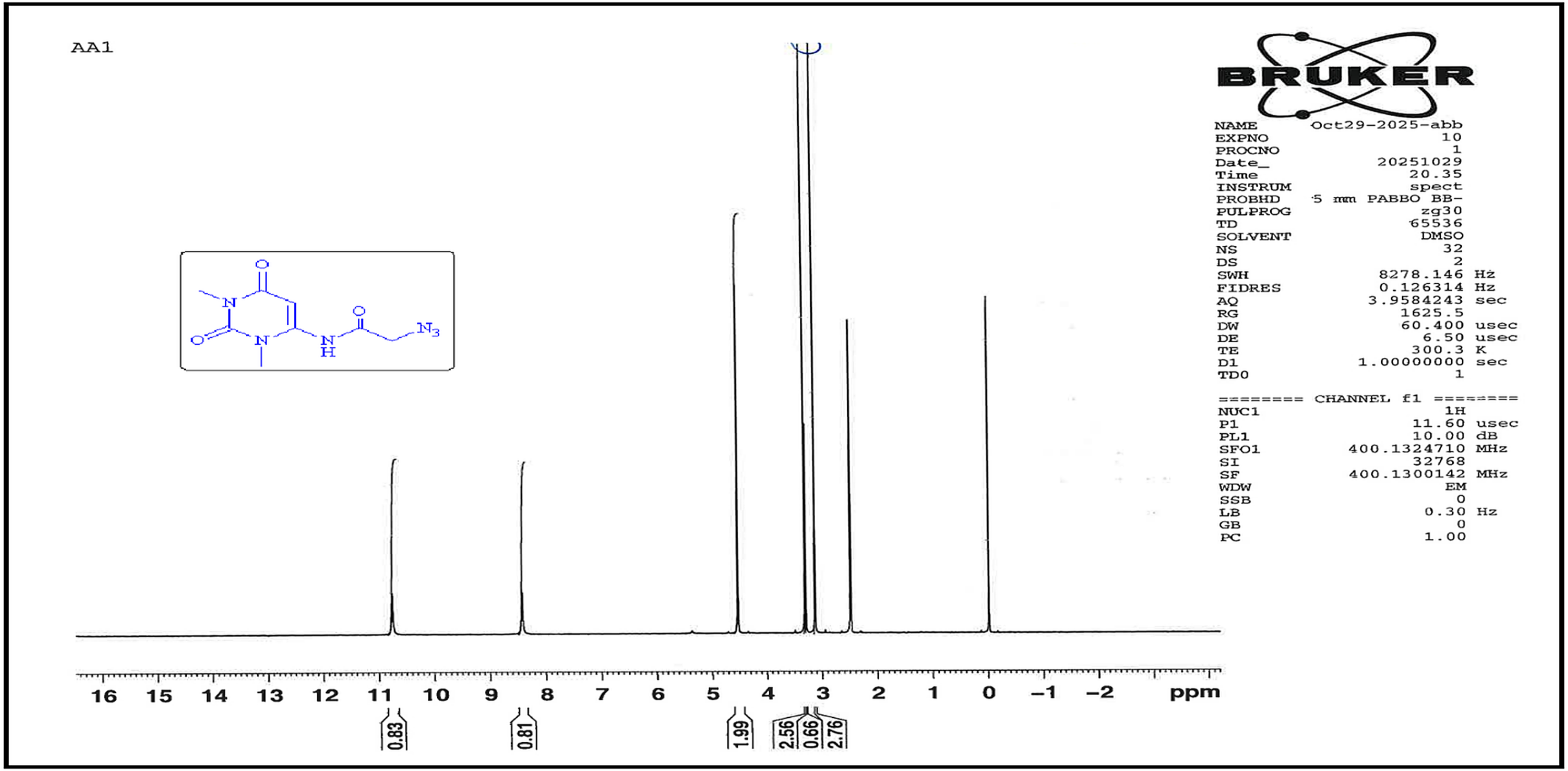

SI figure 8: ^{1}H NMR spectrum of **4**

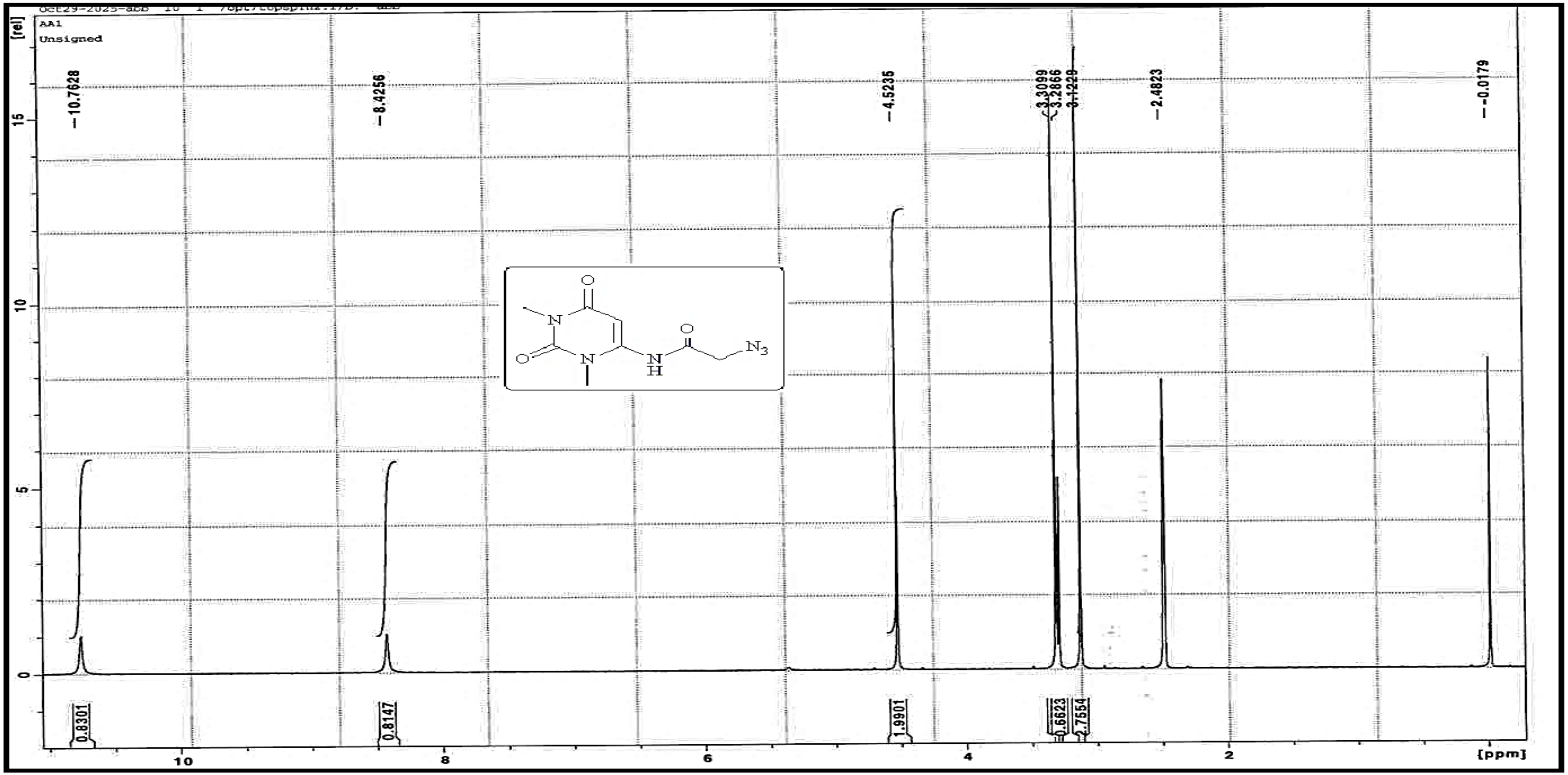

SI figure 9: Expanded ^{1}H NMR spectrum of **4**

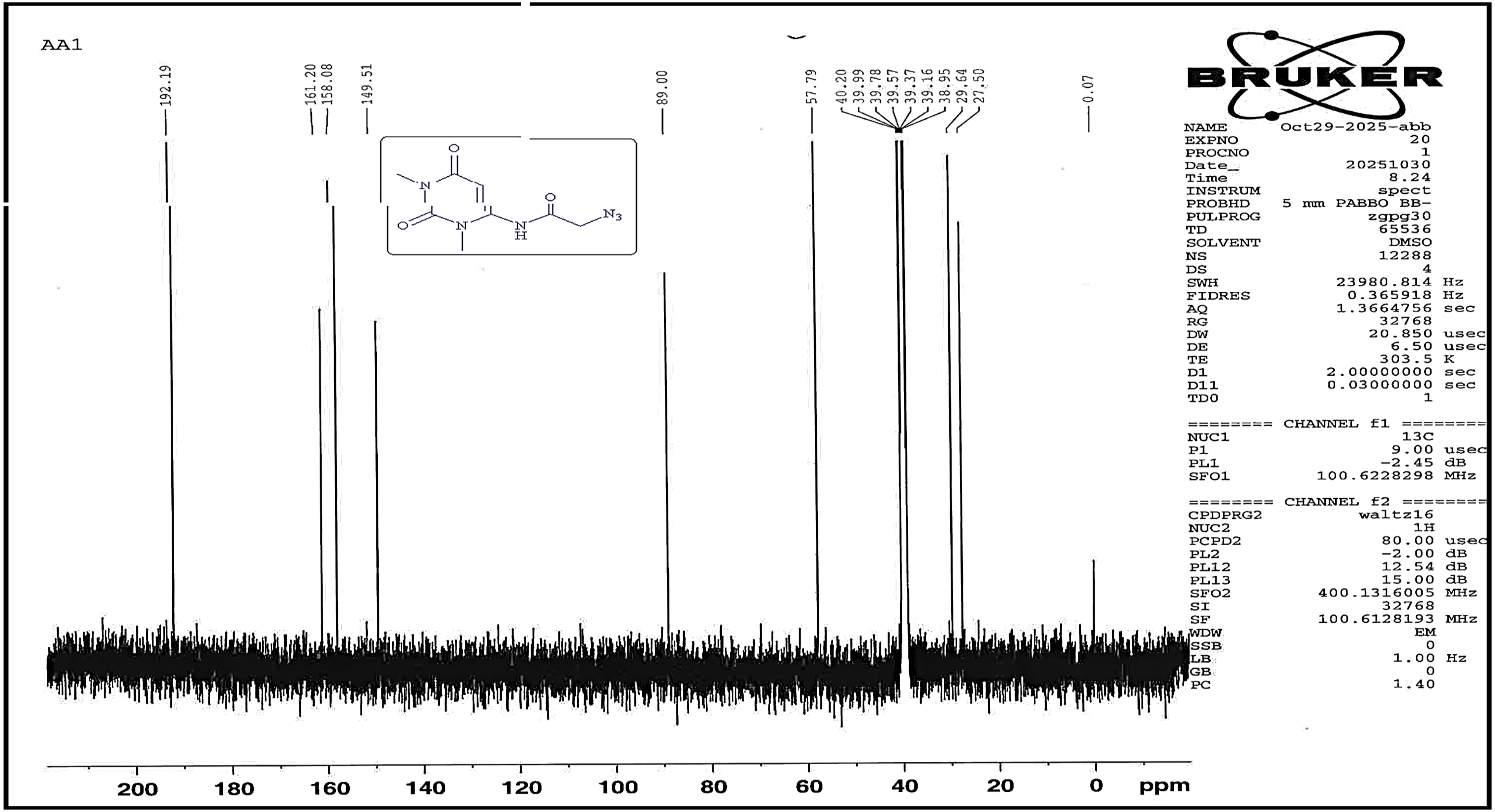

SI figure 10: ^{13}C NMR spectrum of **4**

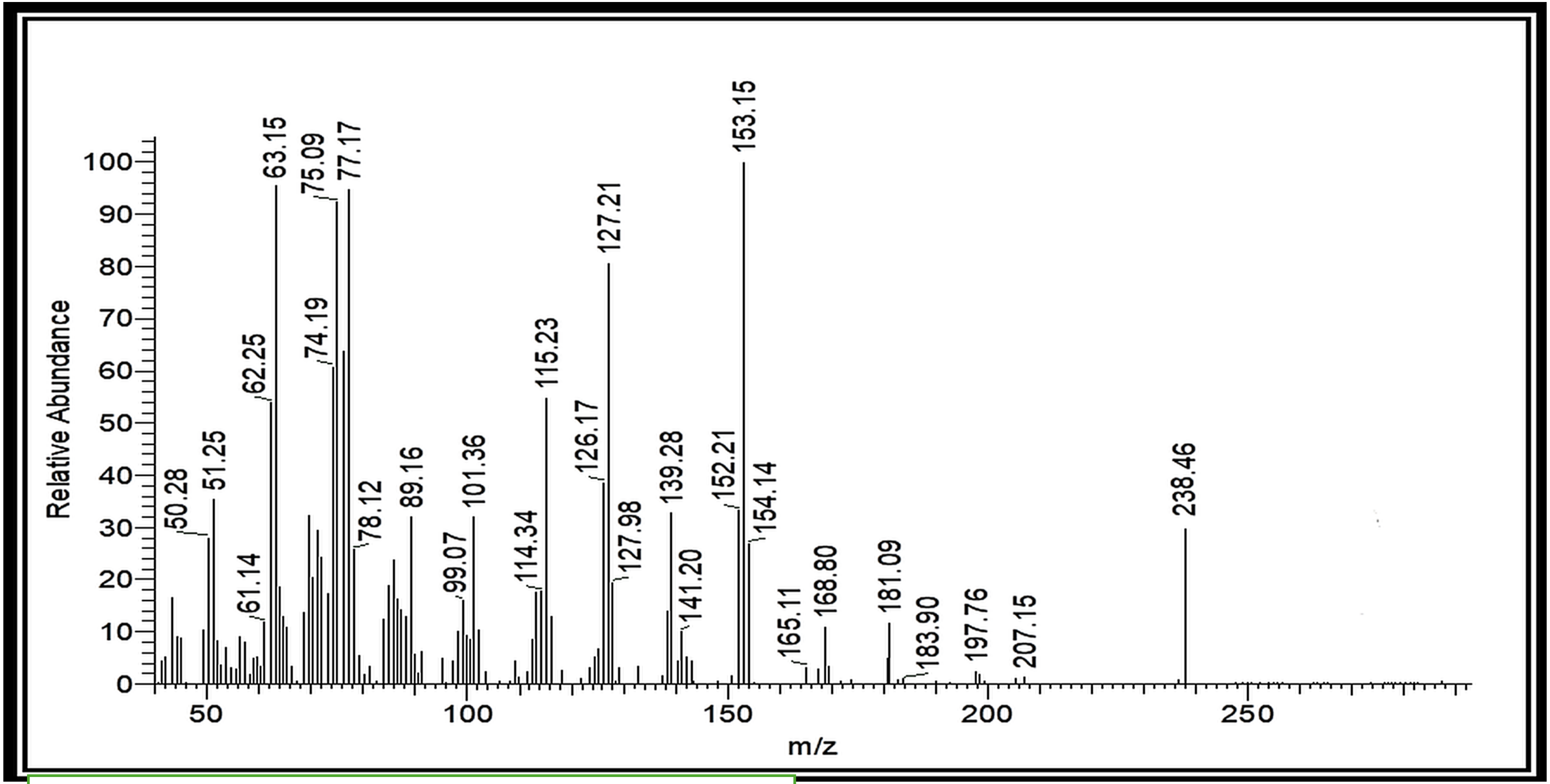

SI figure 11: Mass spectrum of **4**

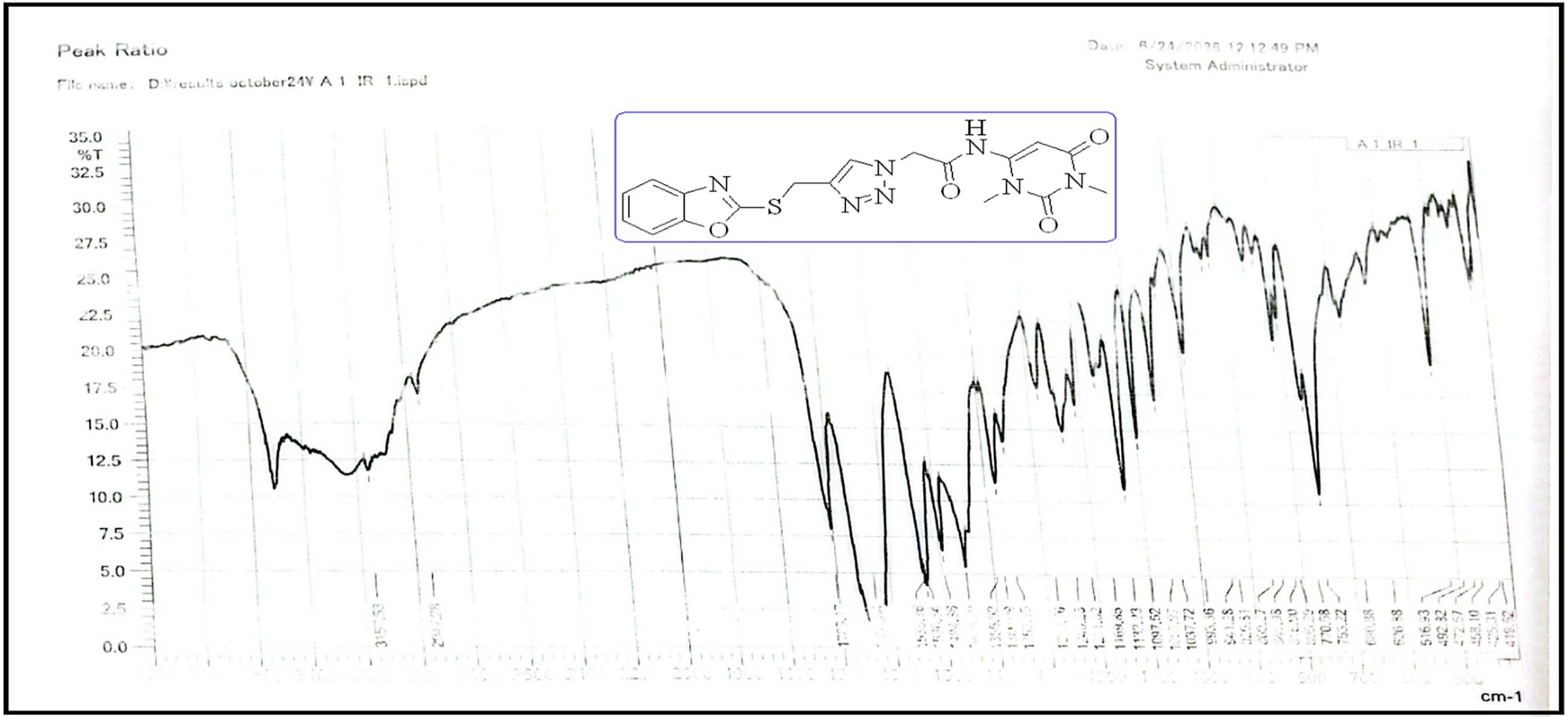

SI figure 12: IR spectrum of compound **6a**

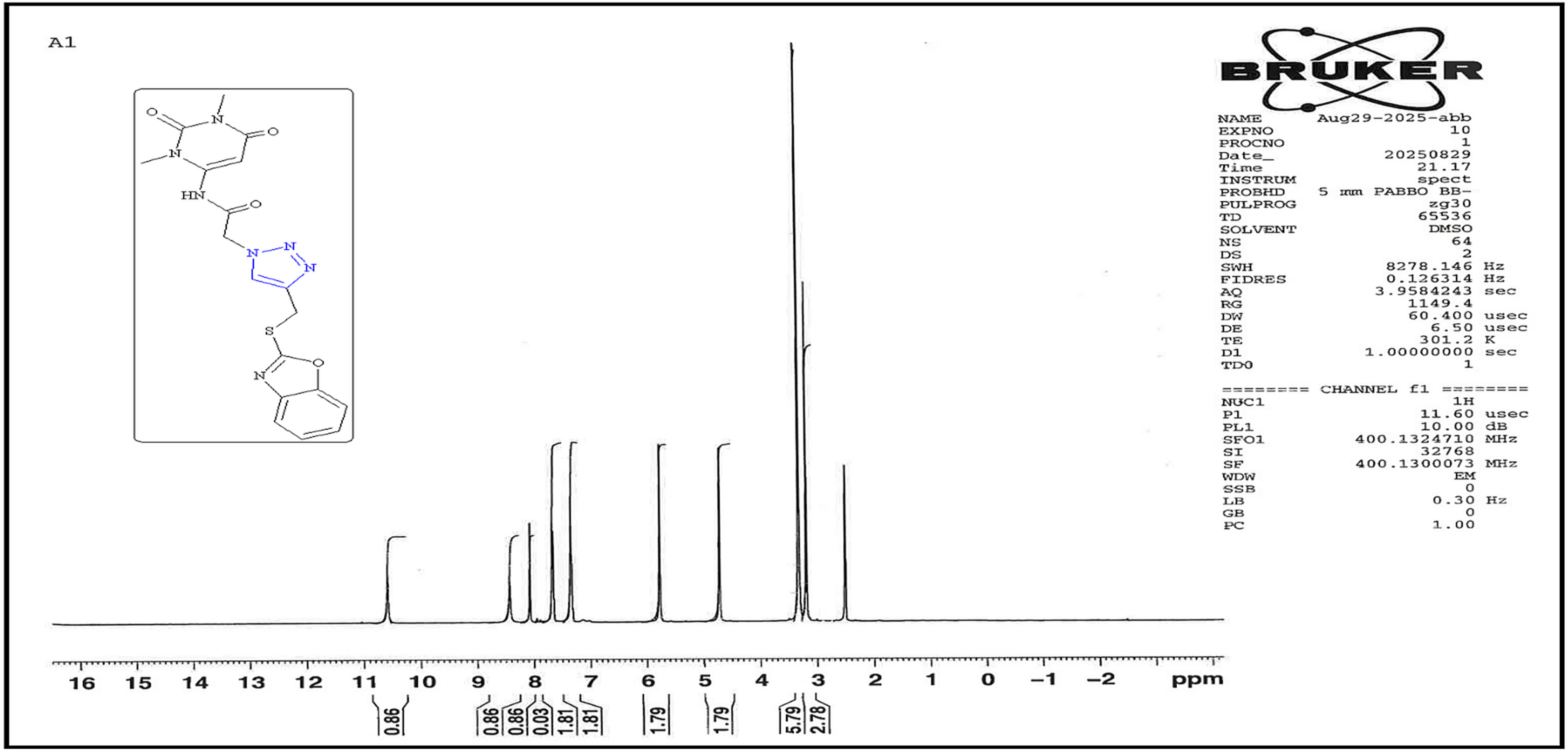

SI figure 13: ^{1}H NMR spectrum of **6a**

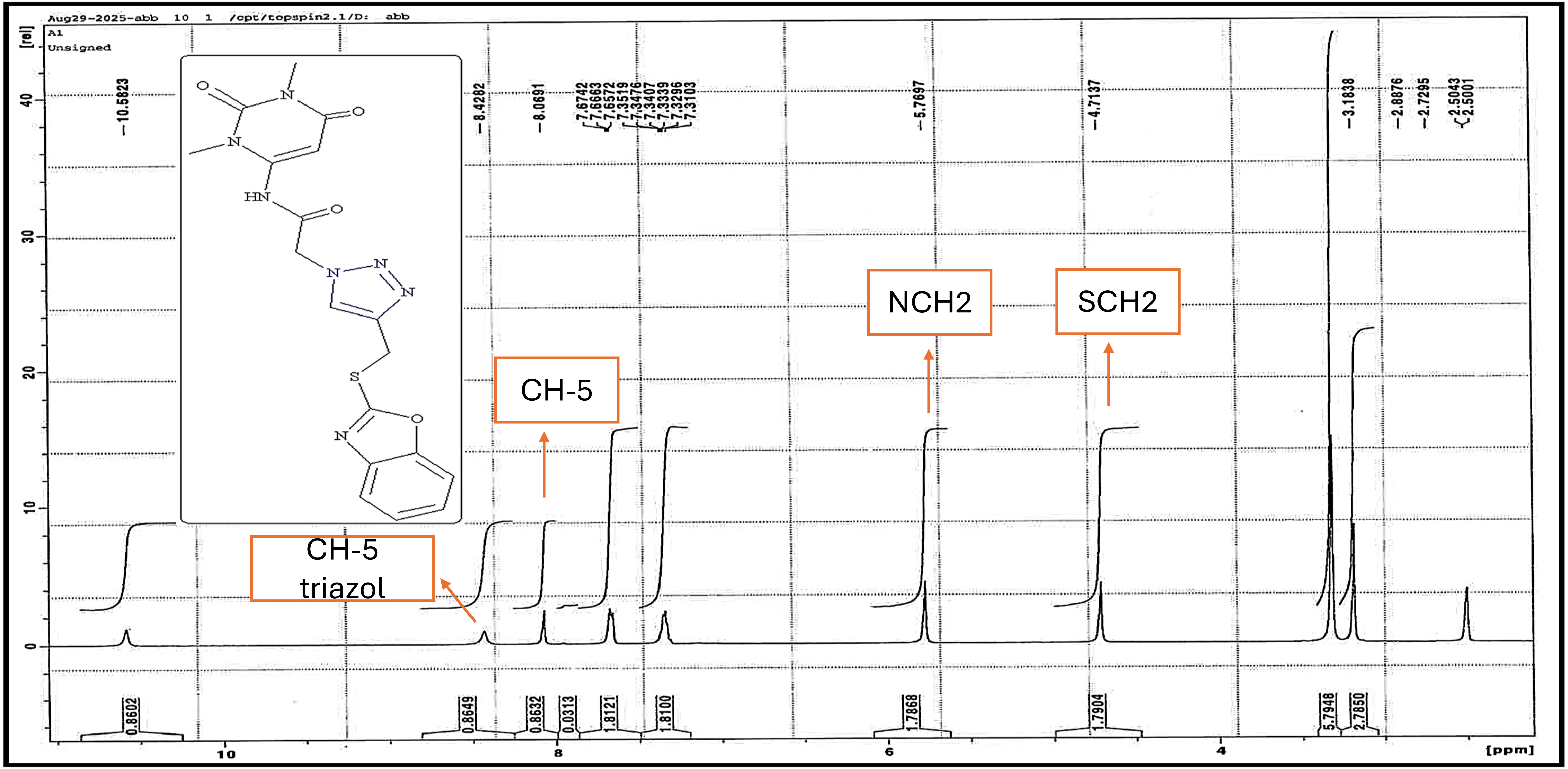

SI figure 14: Expanded ^{1}H NMR spectrum of **6a**

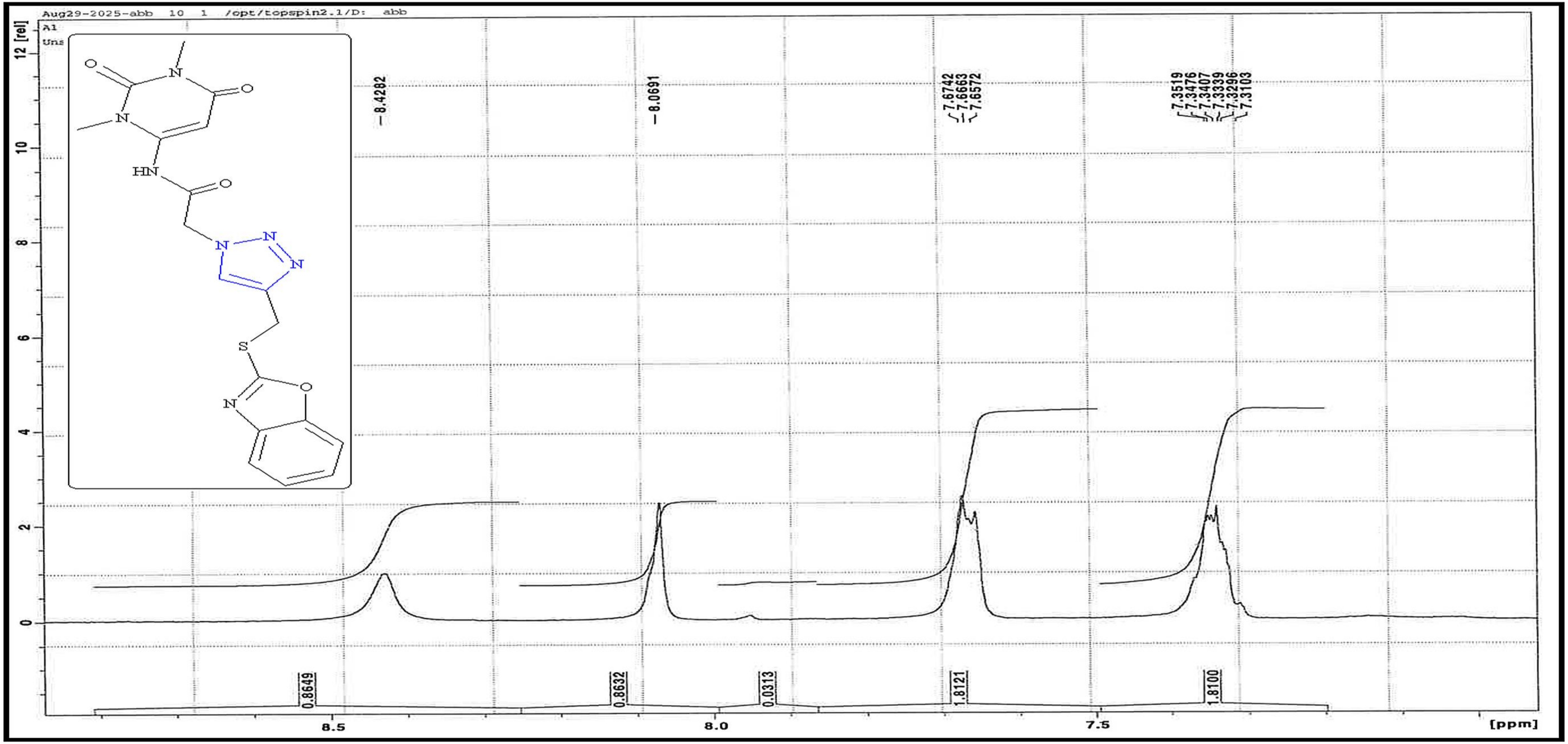

SI figure 15: Expanded ^{1}H NMR spectrum of **6a**

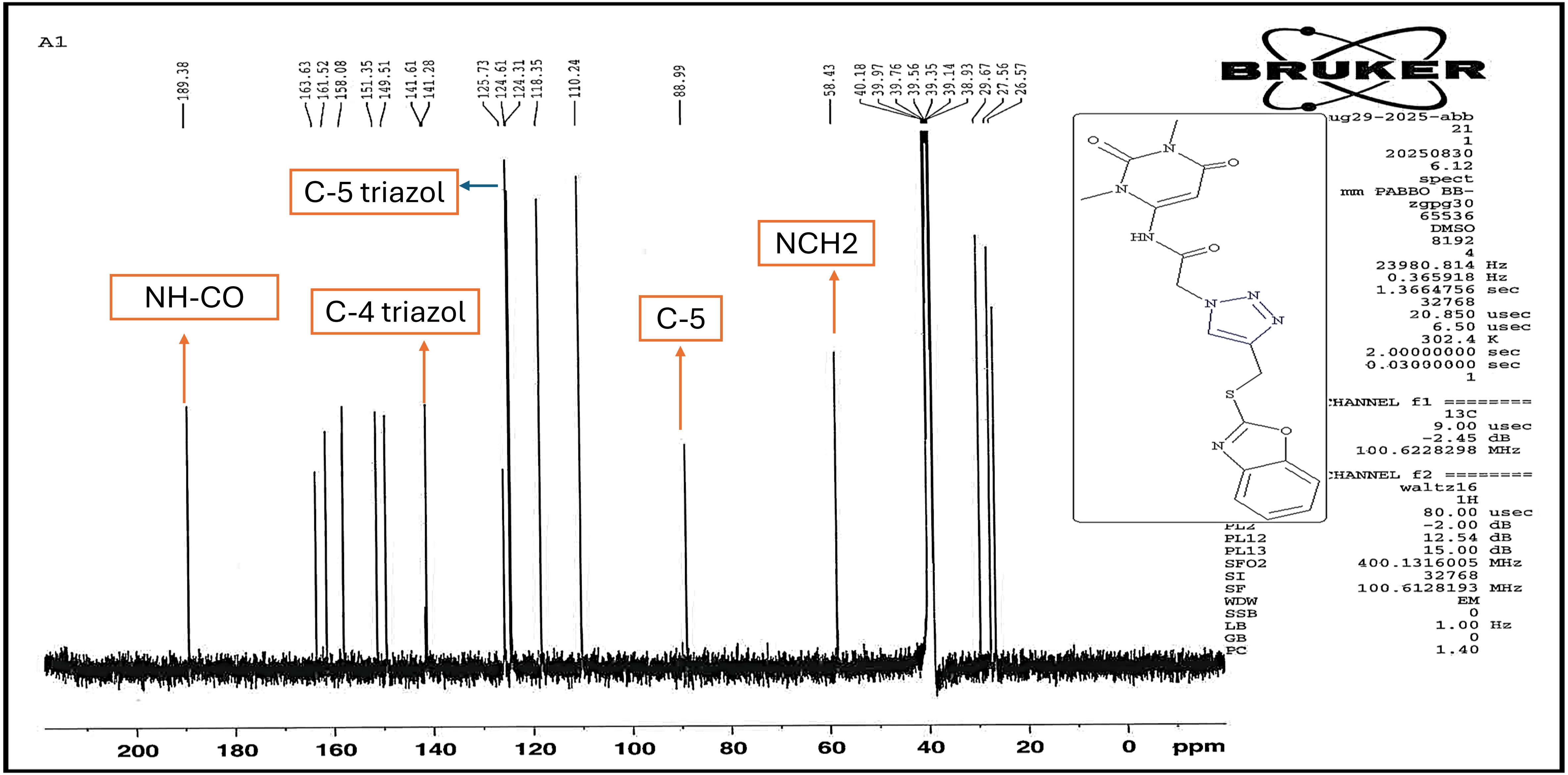

SI figure 16: ^{13}C NMR spectrum of **6a**

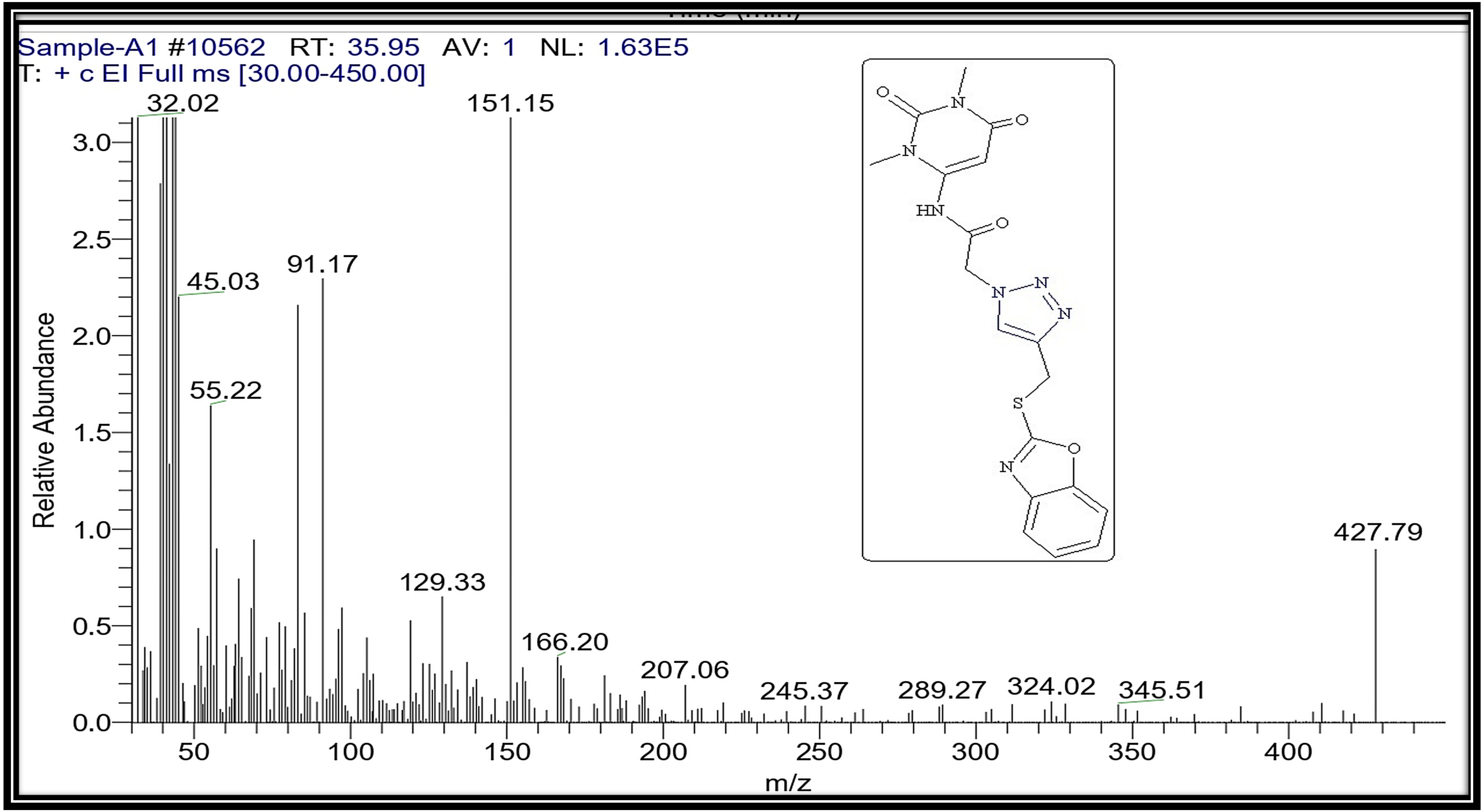

SI figure 17: Mass spectrum of **6a**

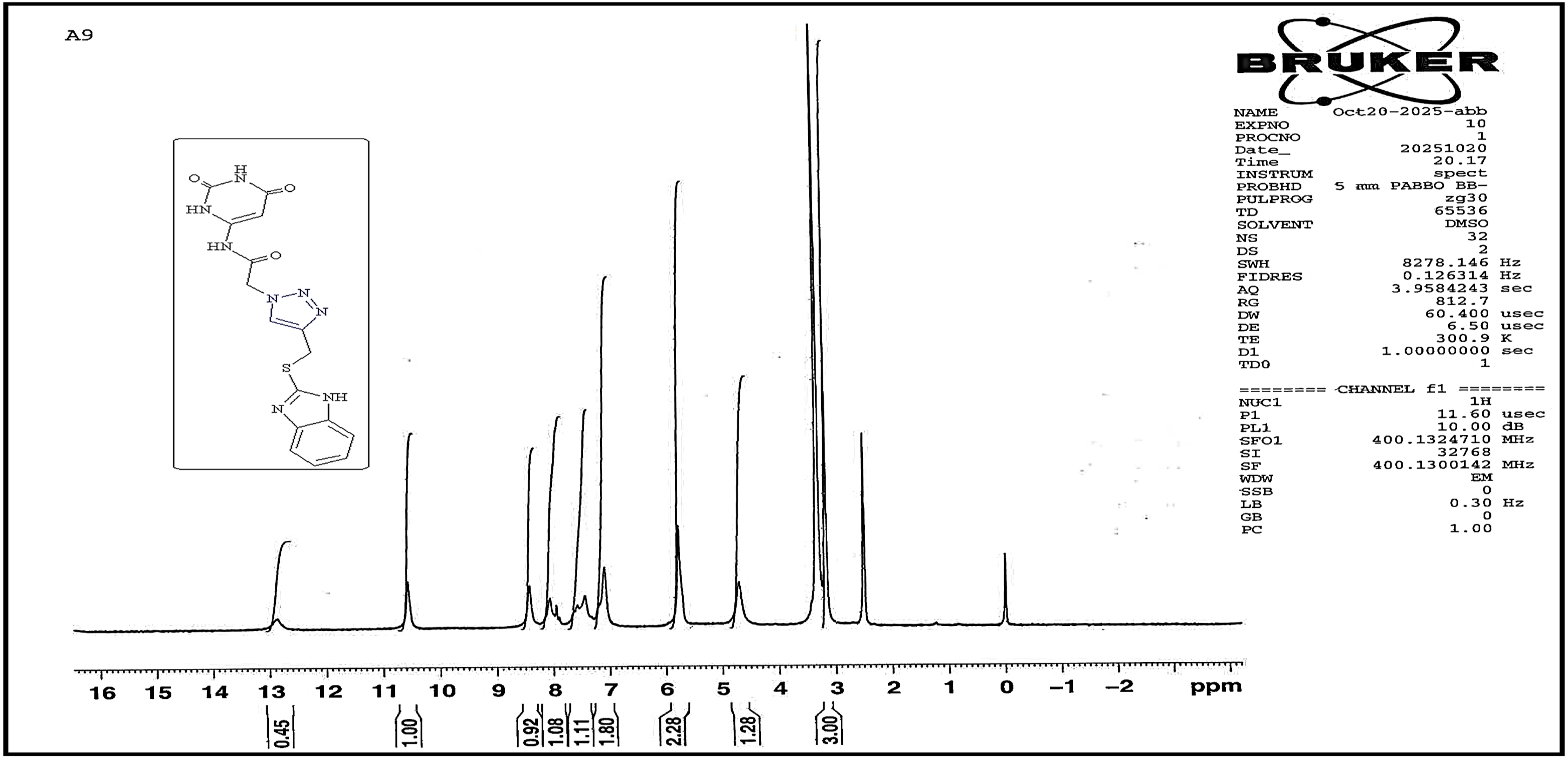

SI figure 18: ^{1}H NMR spectrum of **6b**

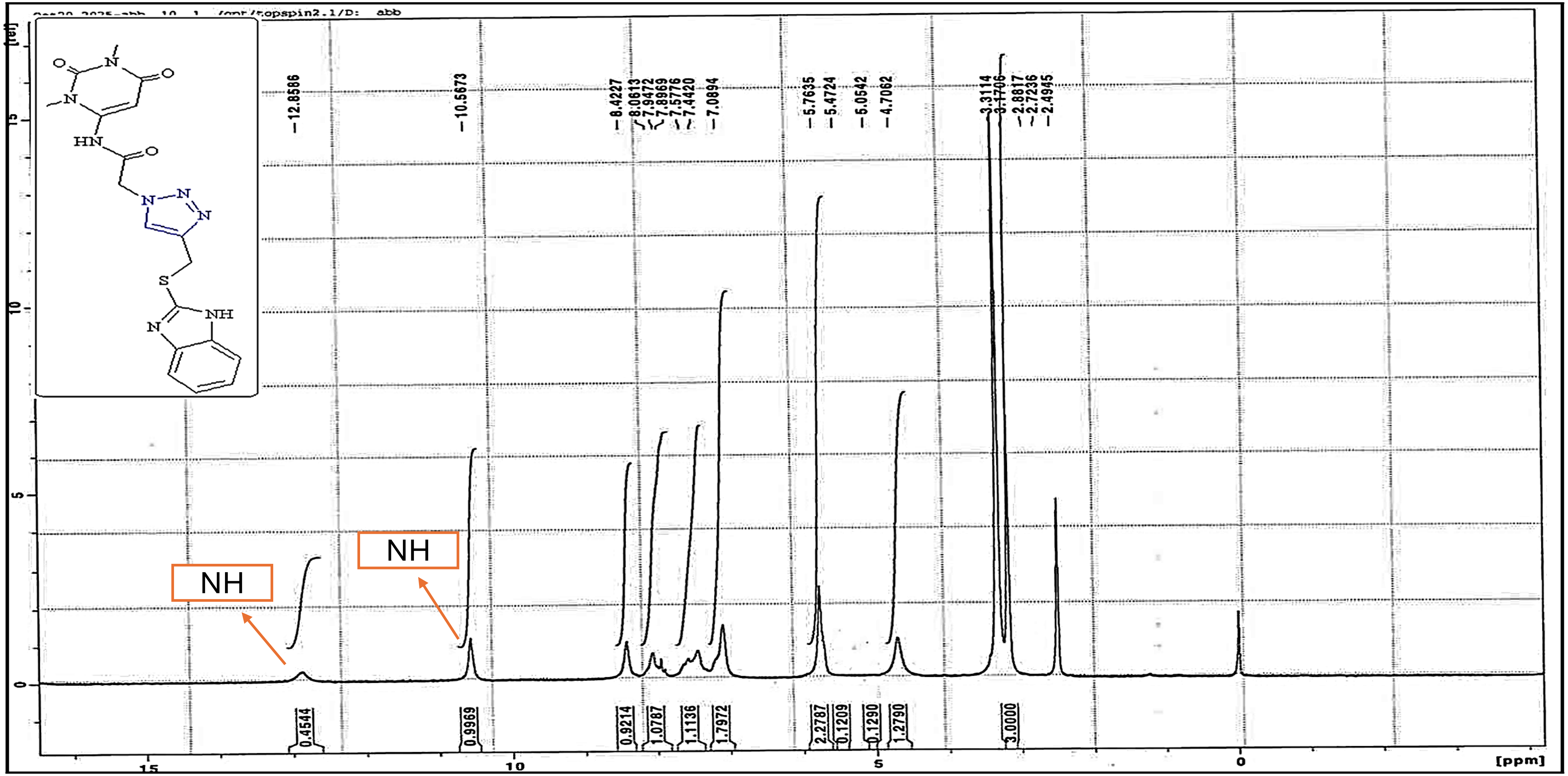

SI figure 19: Expanded ^{1}H NMR spectrum of **6b**

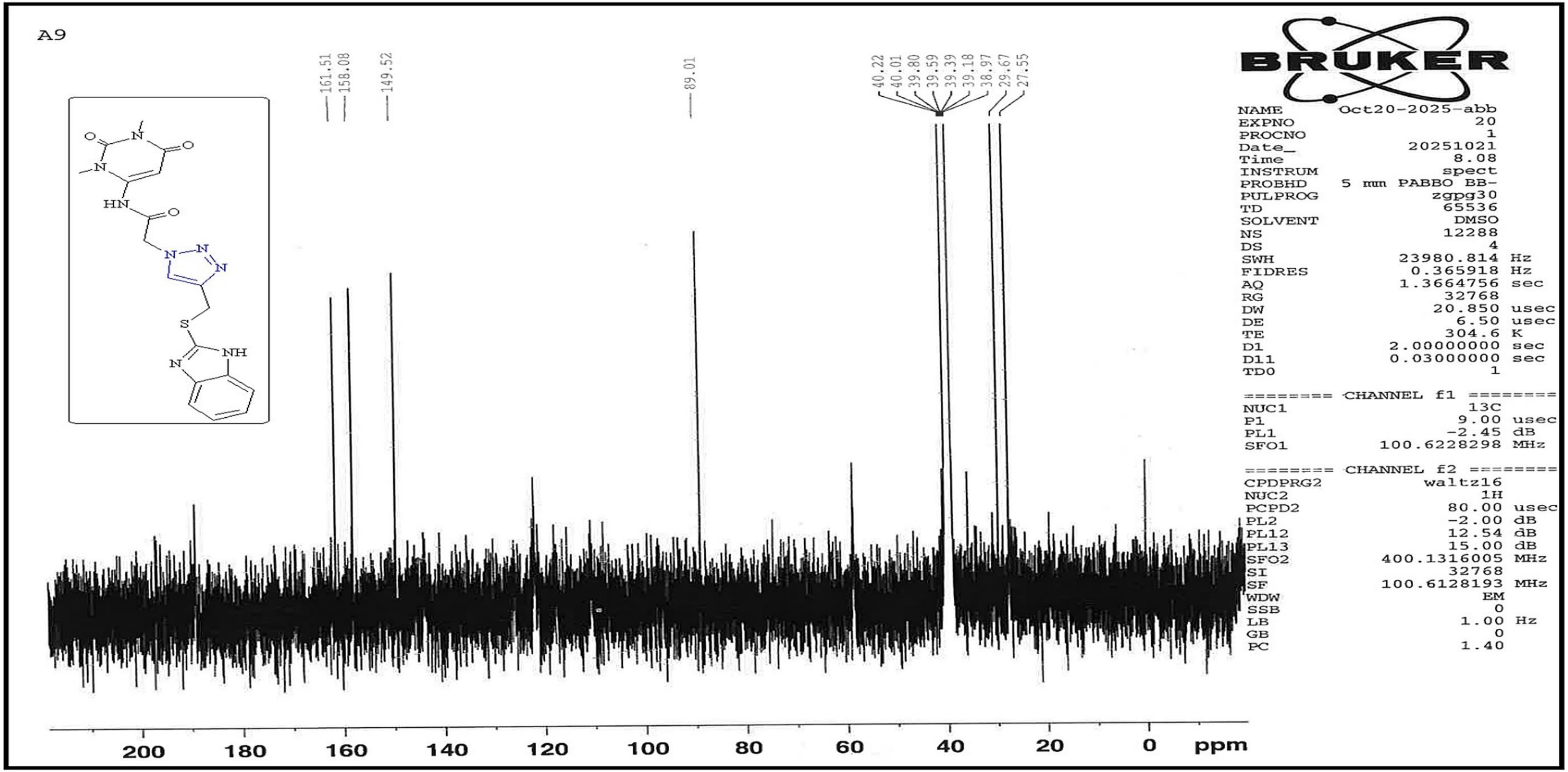

SI figure 20: ^{13}C NMR spectrum of **6b**

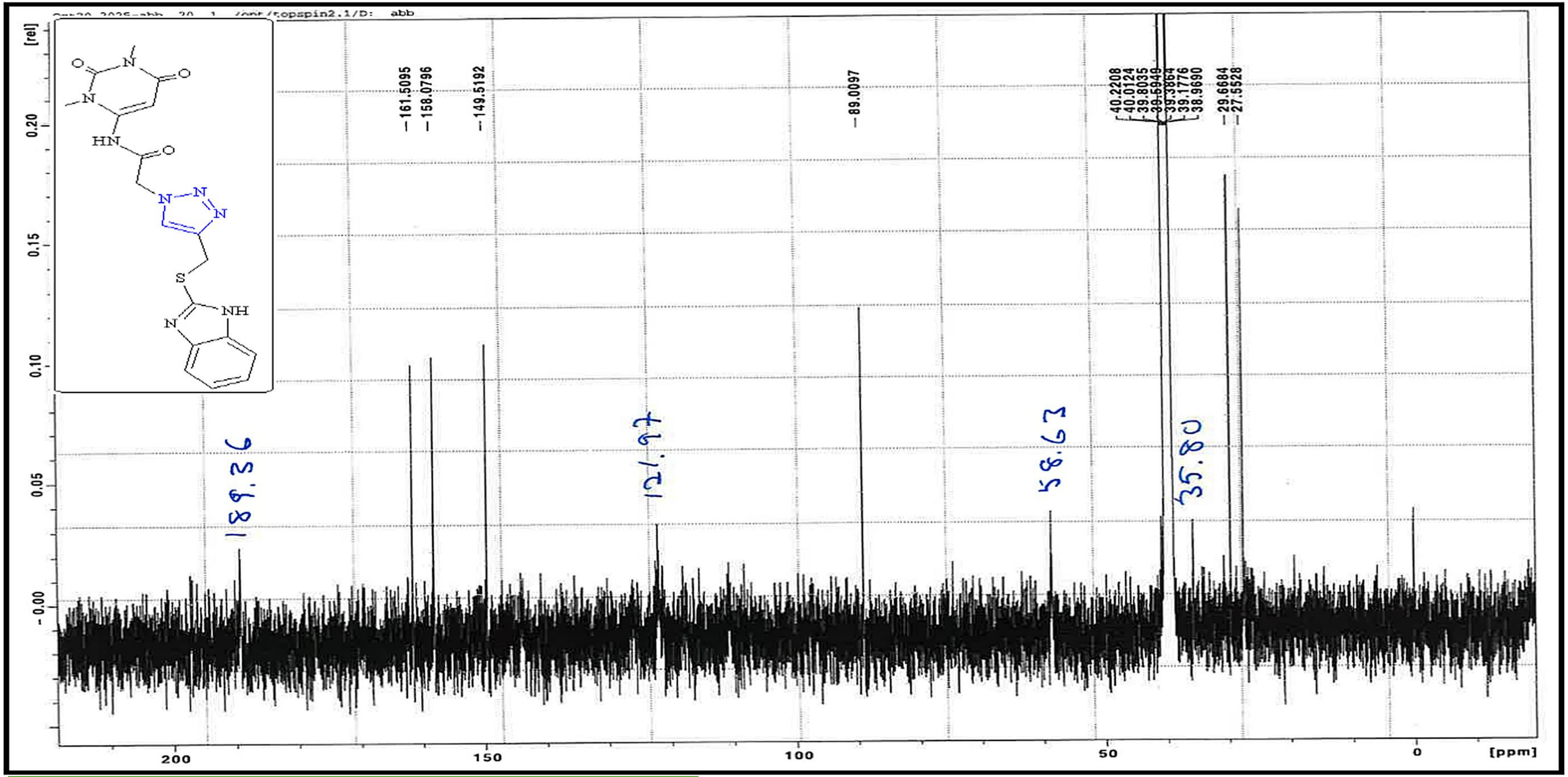

SI figure 21: Expanded ^{13}C NMR spectrum of **6b**

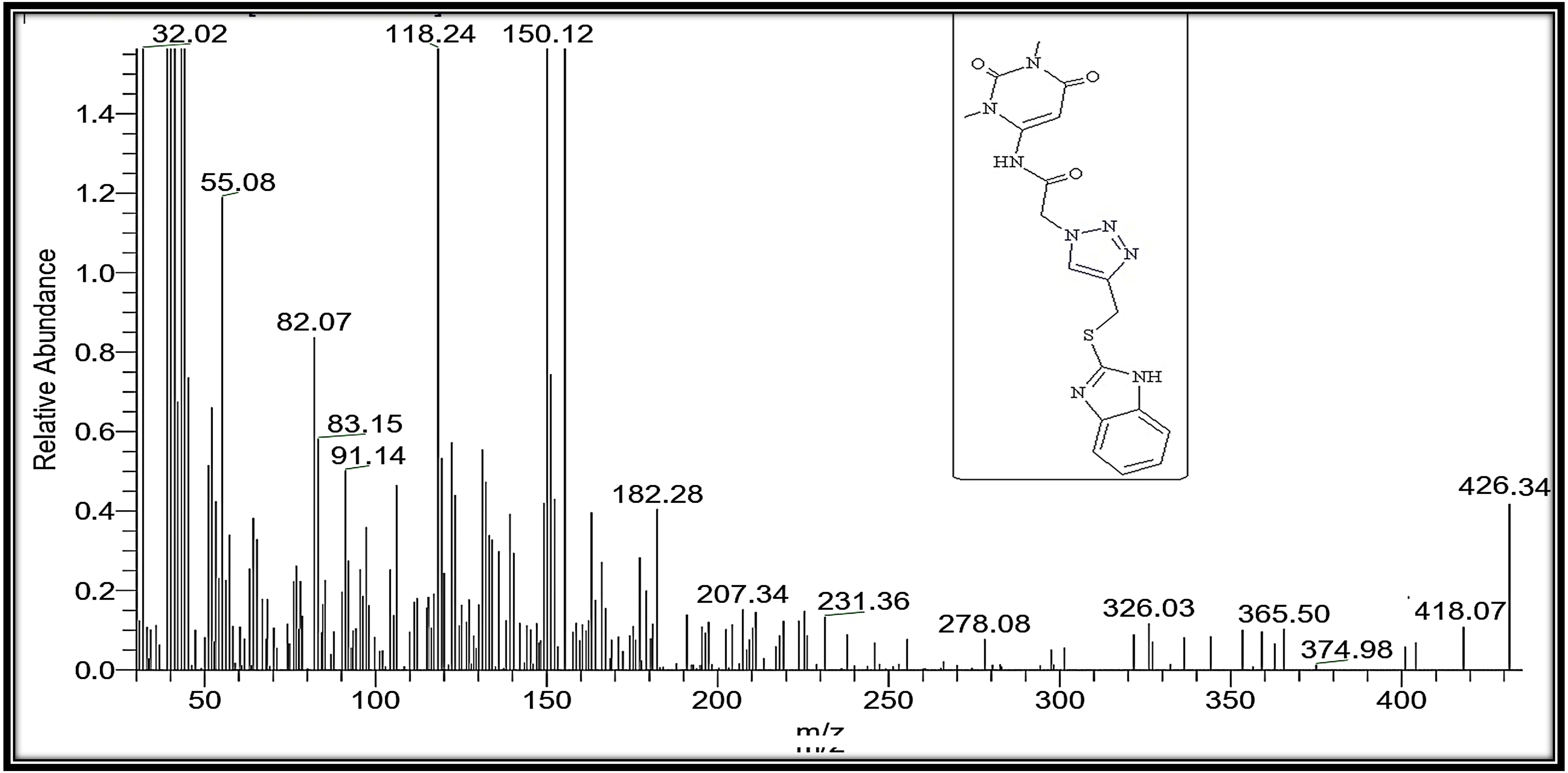

SI figure 26: Mass spectrum of **8a**

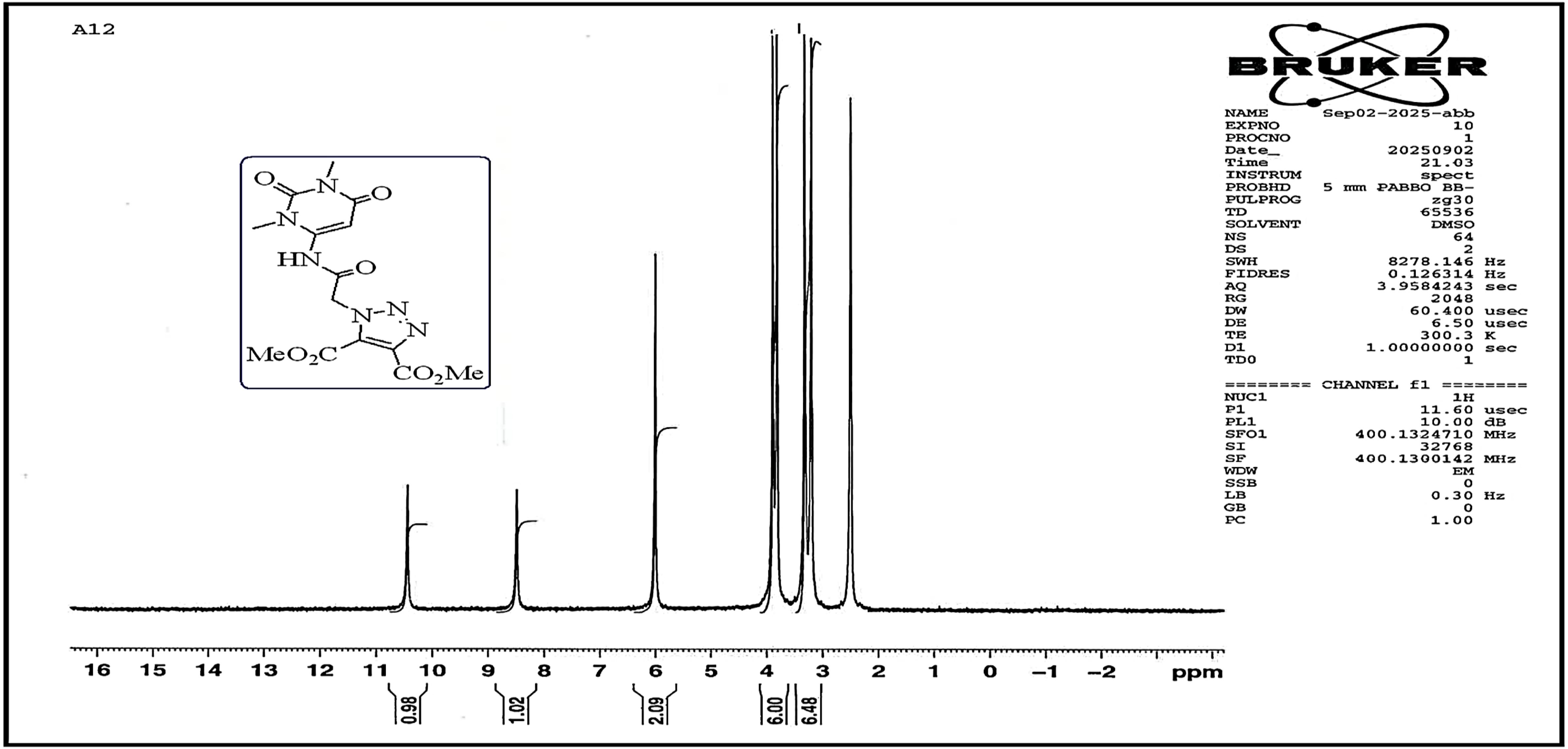

SI figure 23: ^{1}H NMR spectrum of **8a**

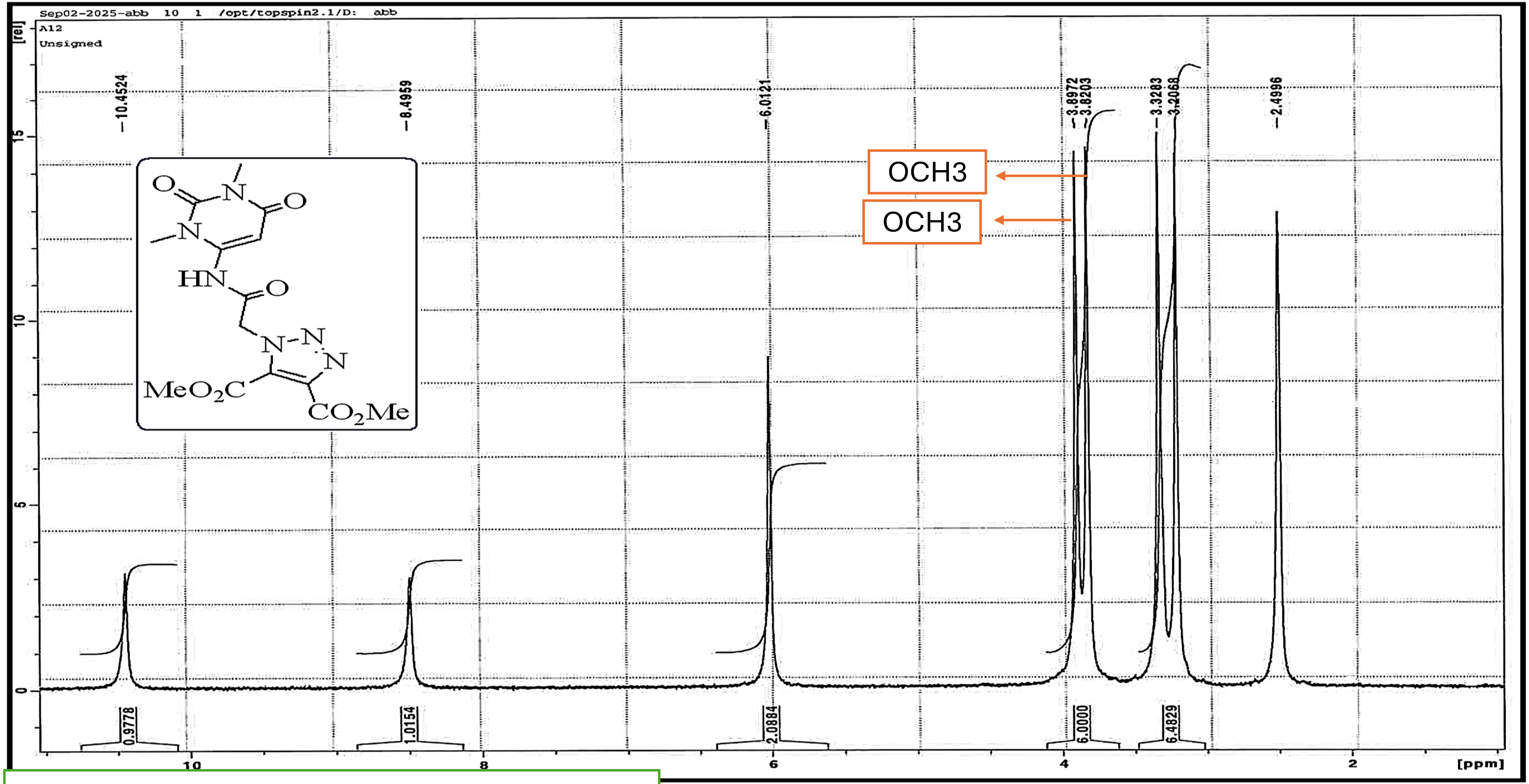

SI figure 24: Expanded ^{1}H NMR spectrum of **8a**

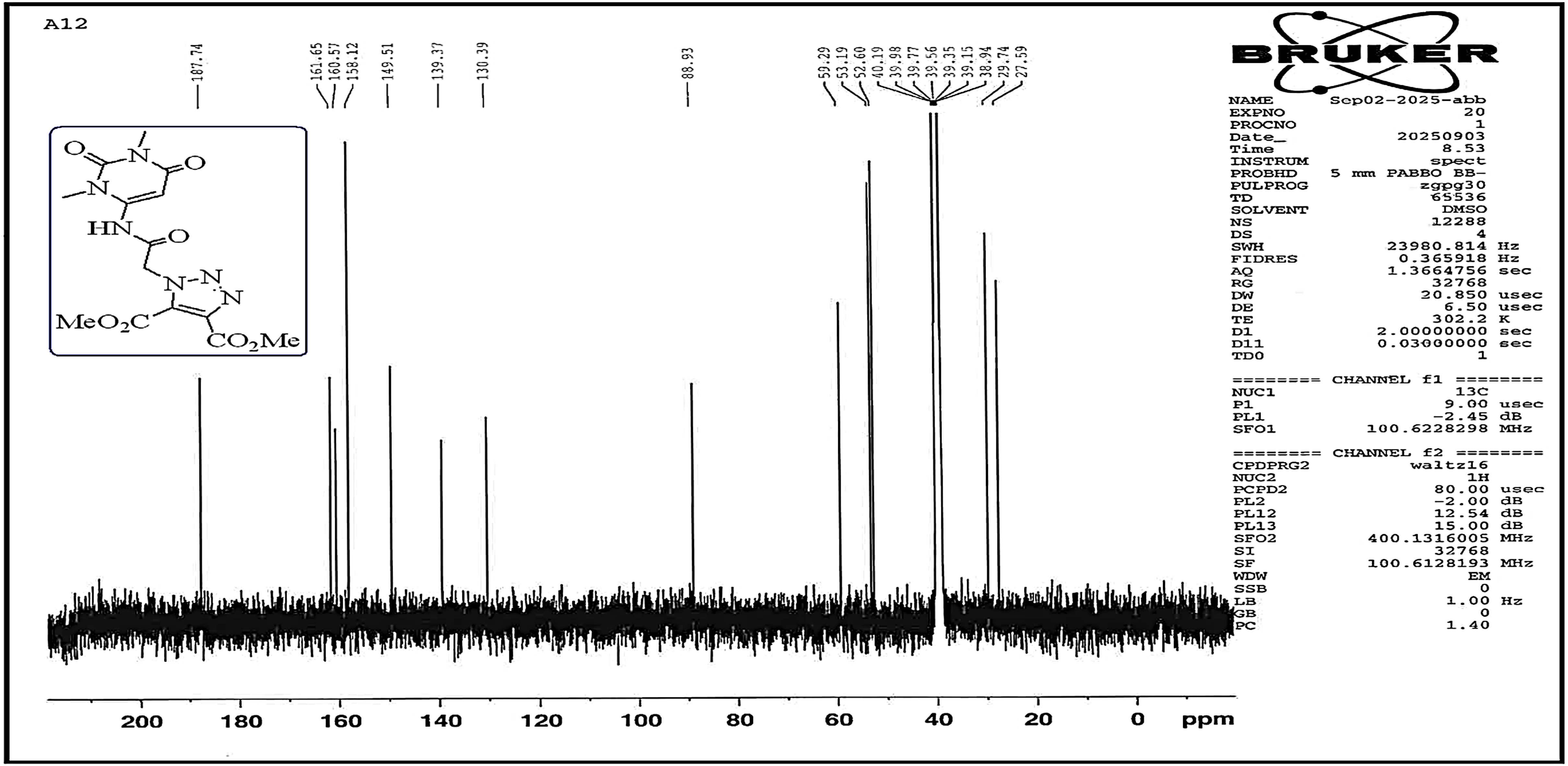

SI figure 25: ^{13}C NMR spectrum of **8a**

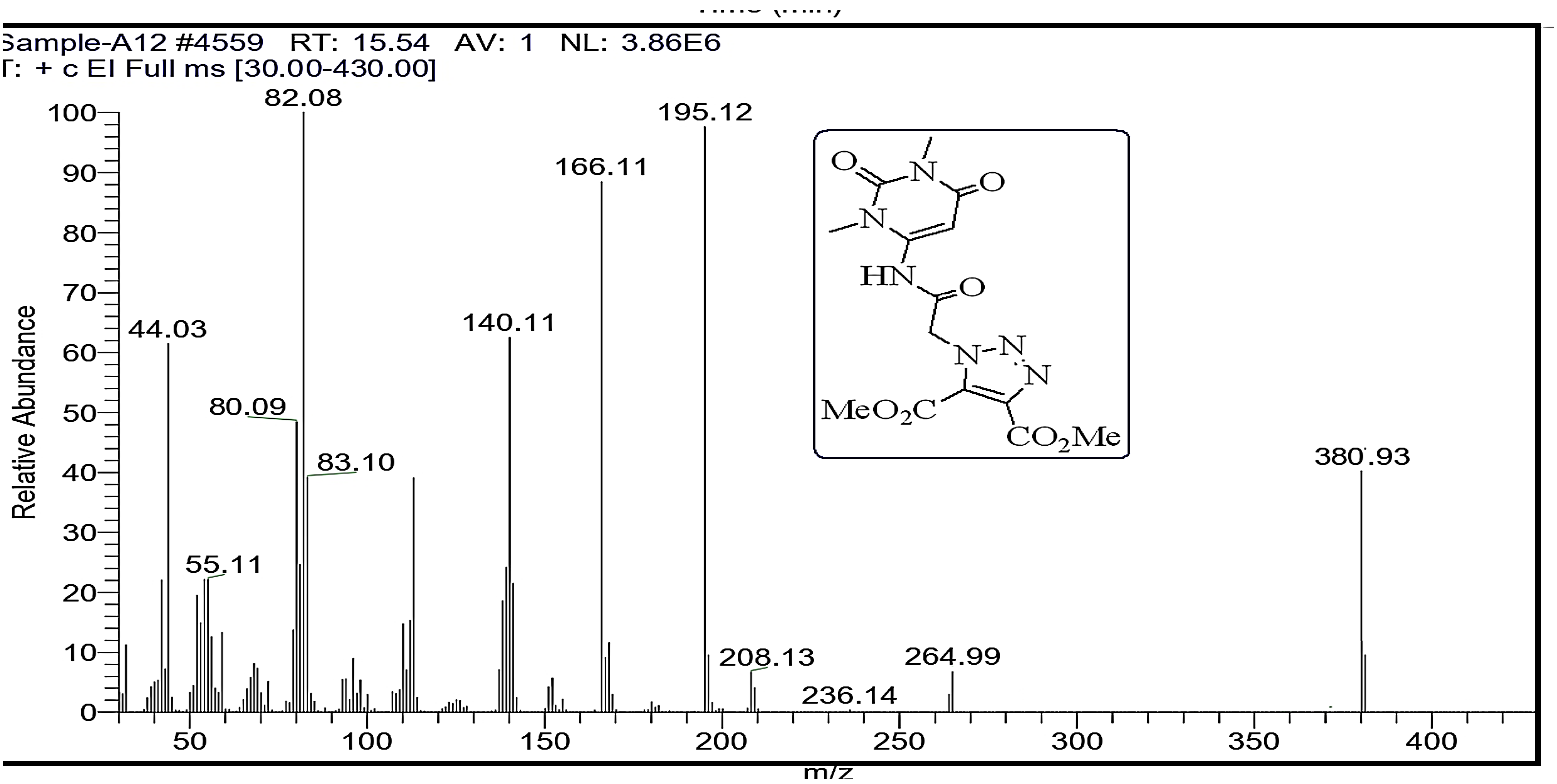

SI figure 26: Mass spectrum of **8a**

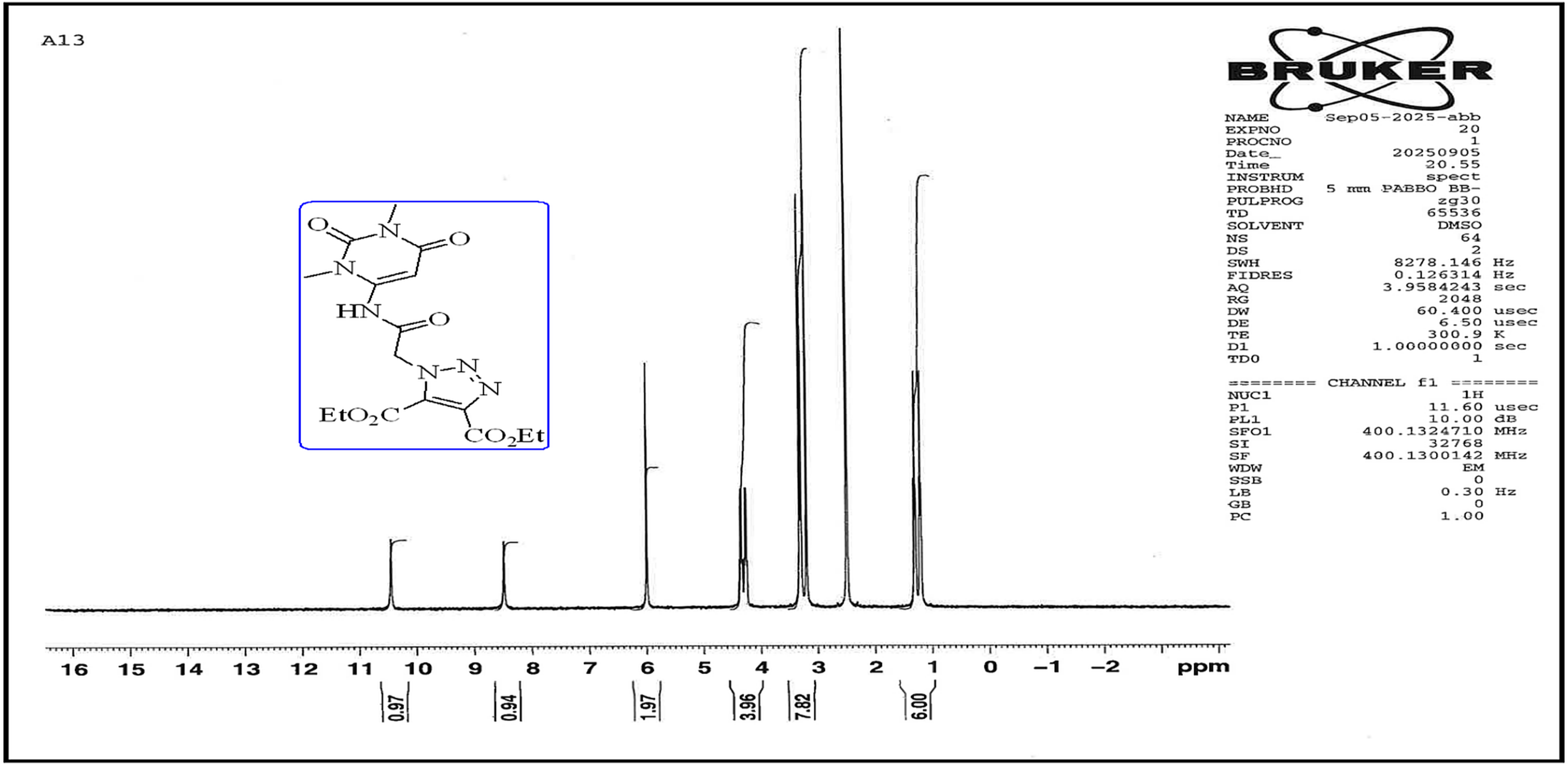

SI figure 27: ^{1}H NMR spectrum of **8b**

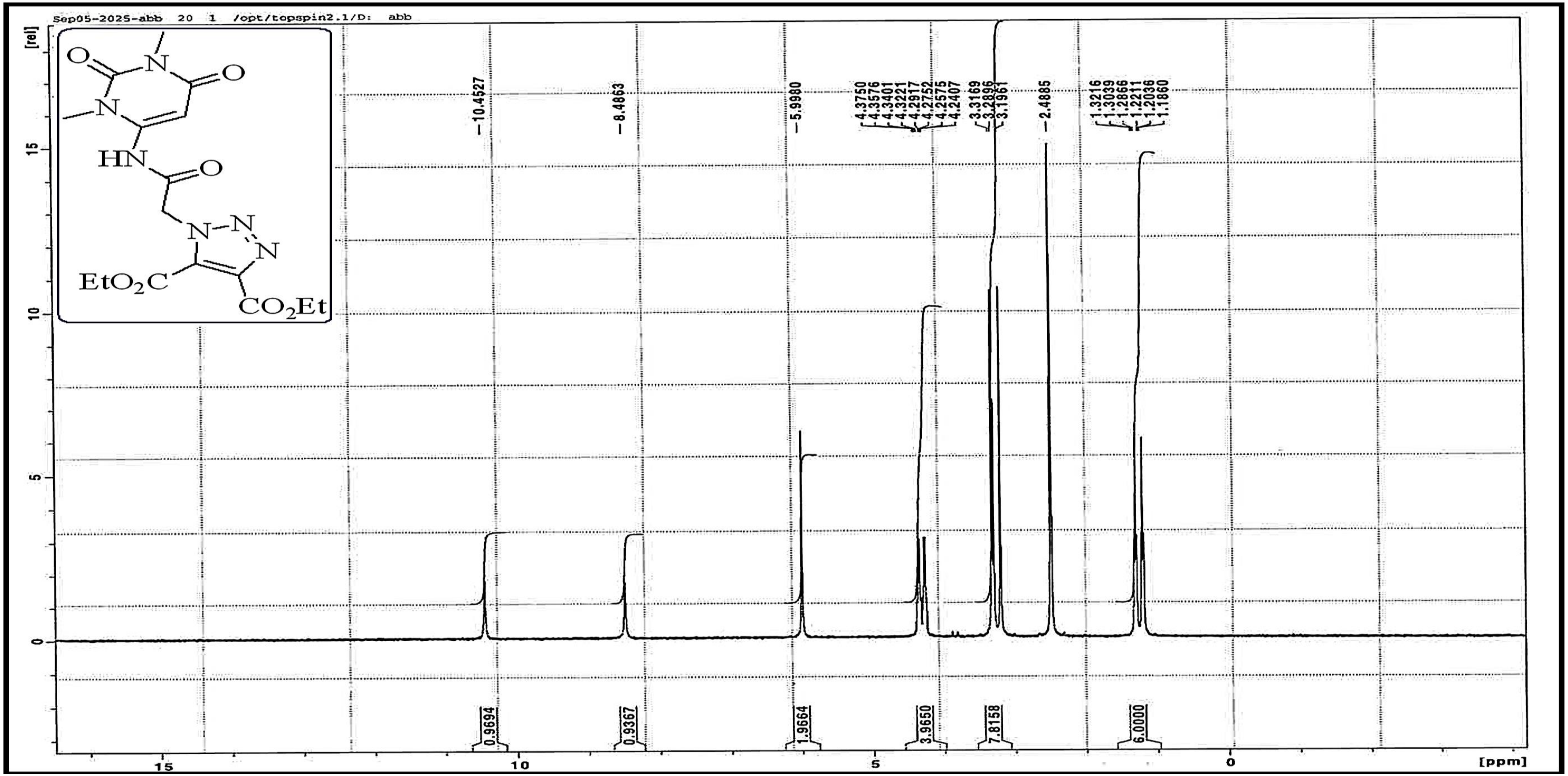

SI figure 28: Expanded ^{1}H NMR spectrum of **8b**

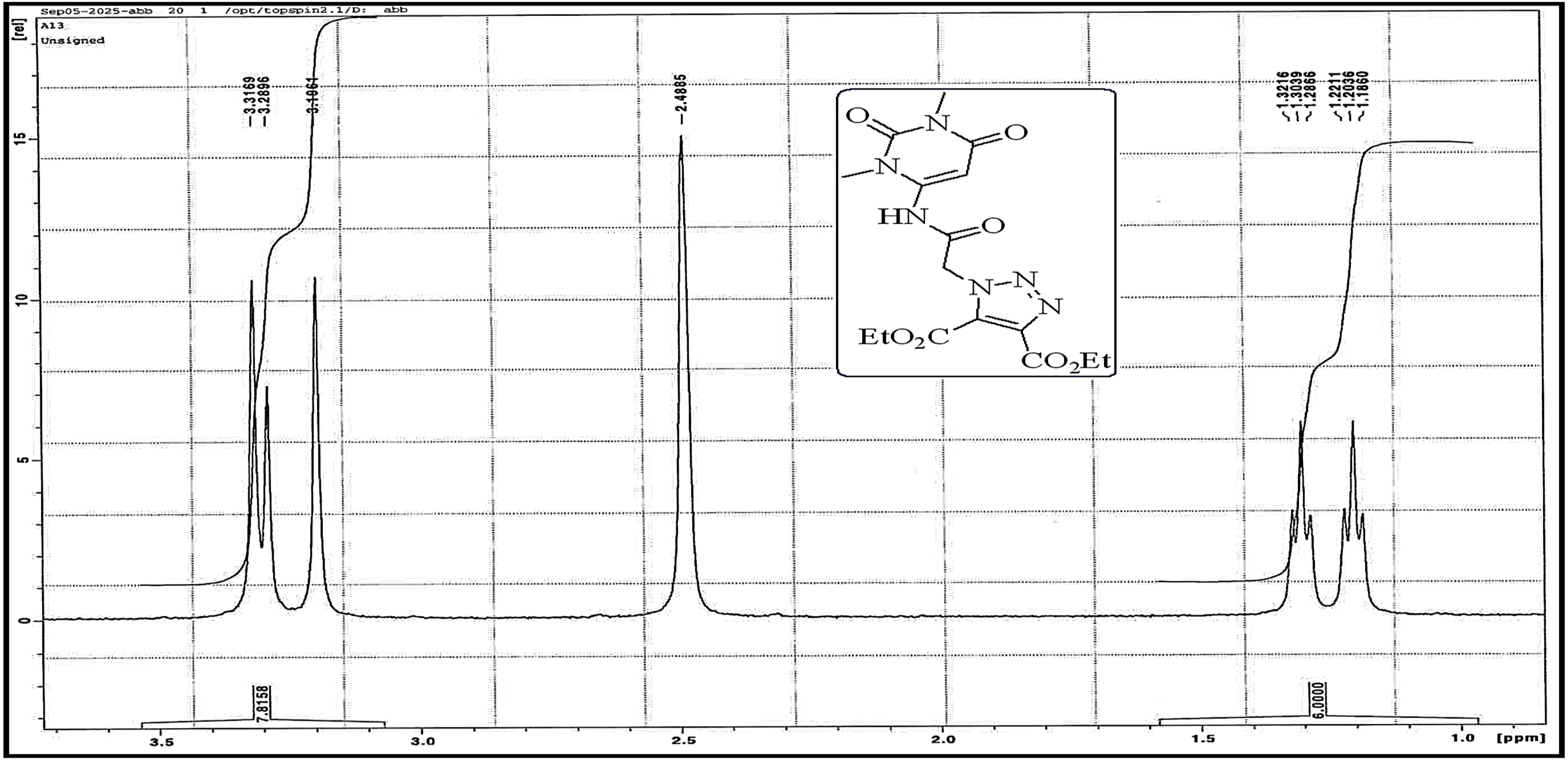

SI figure 29: Expanded ^{1}H NMR spectrum of **8b**

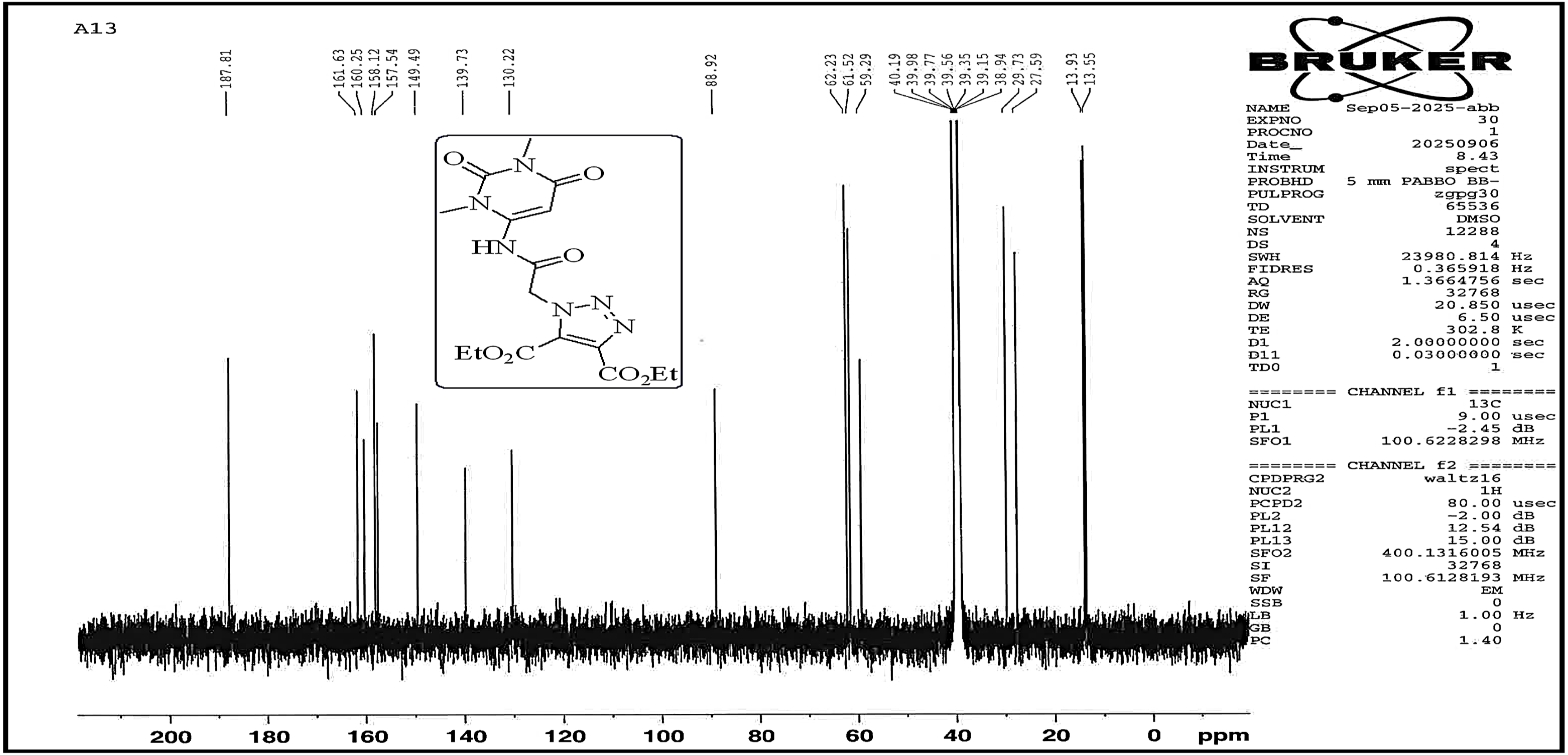

SI figure 30: ^{13}C NMR spectrum of **8b**

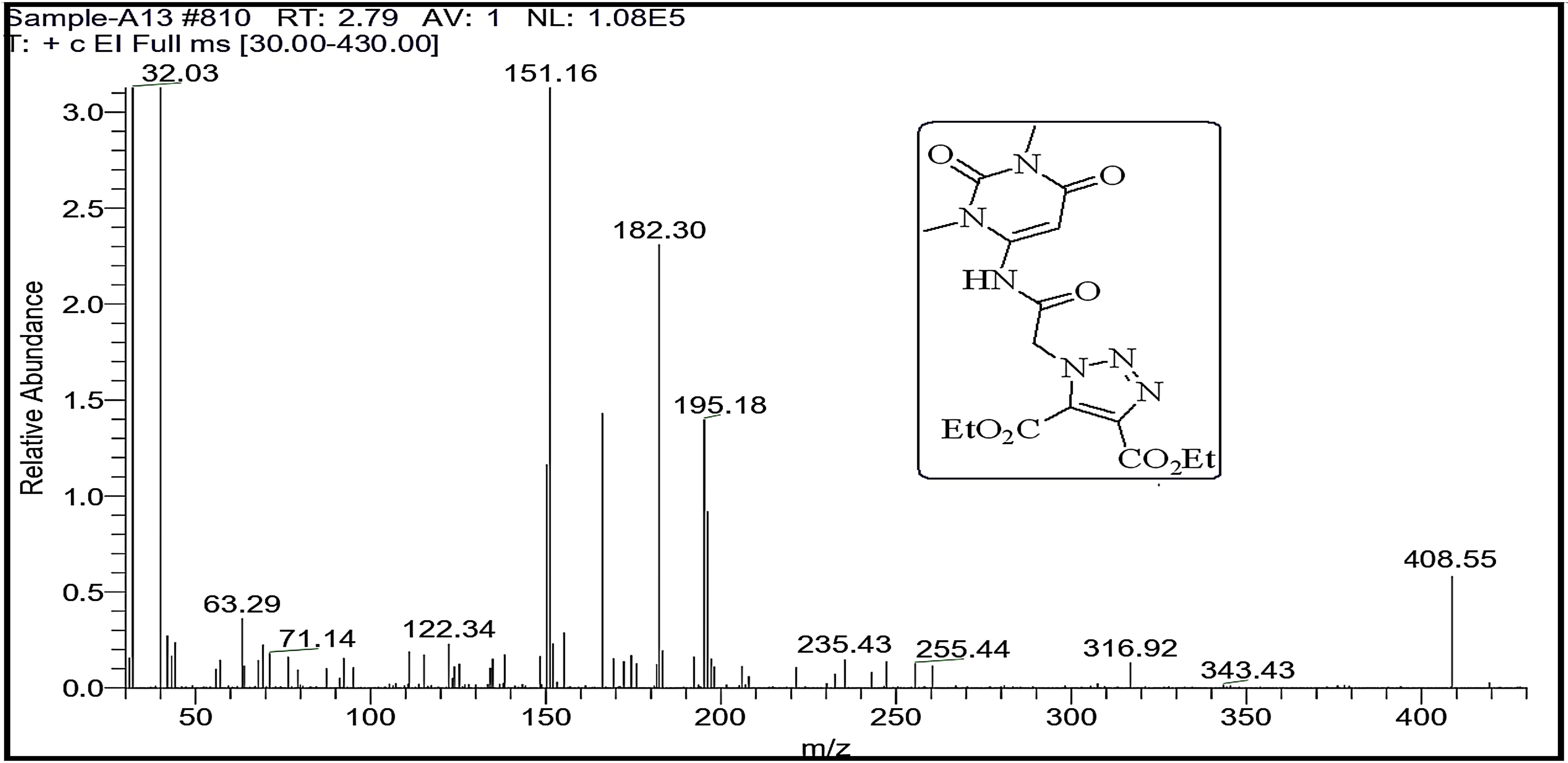

SI figure 31: Mass spectrum of **8b**

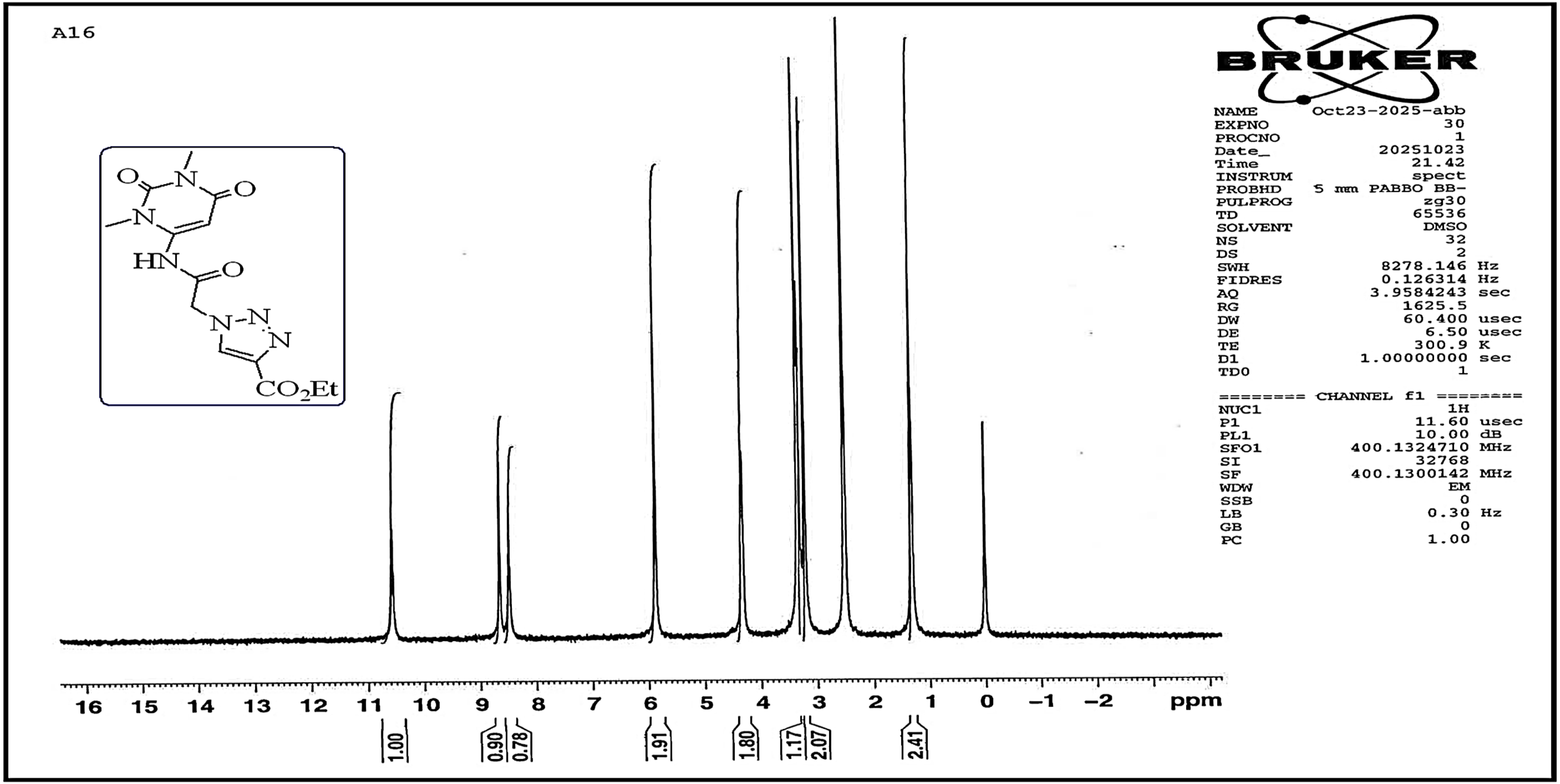

SI figure 32: ^{1}H NMR spectrum of **8c**

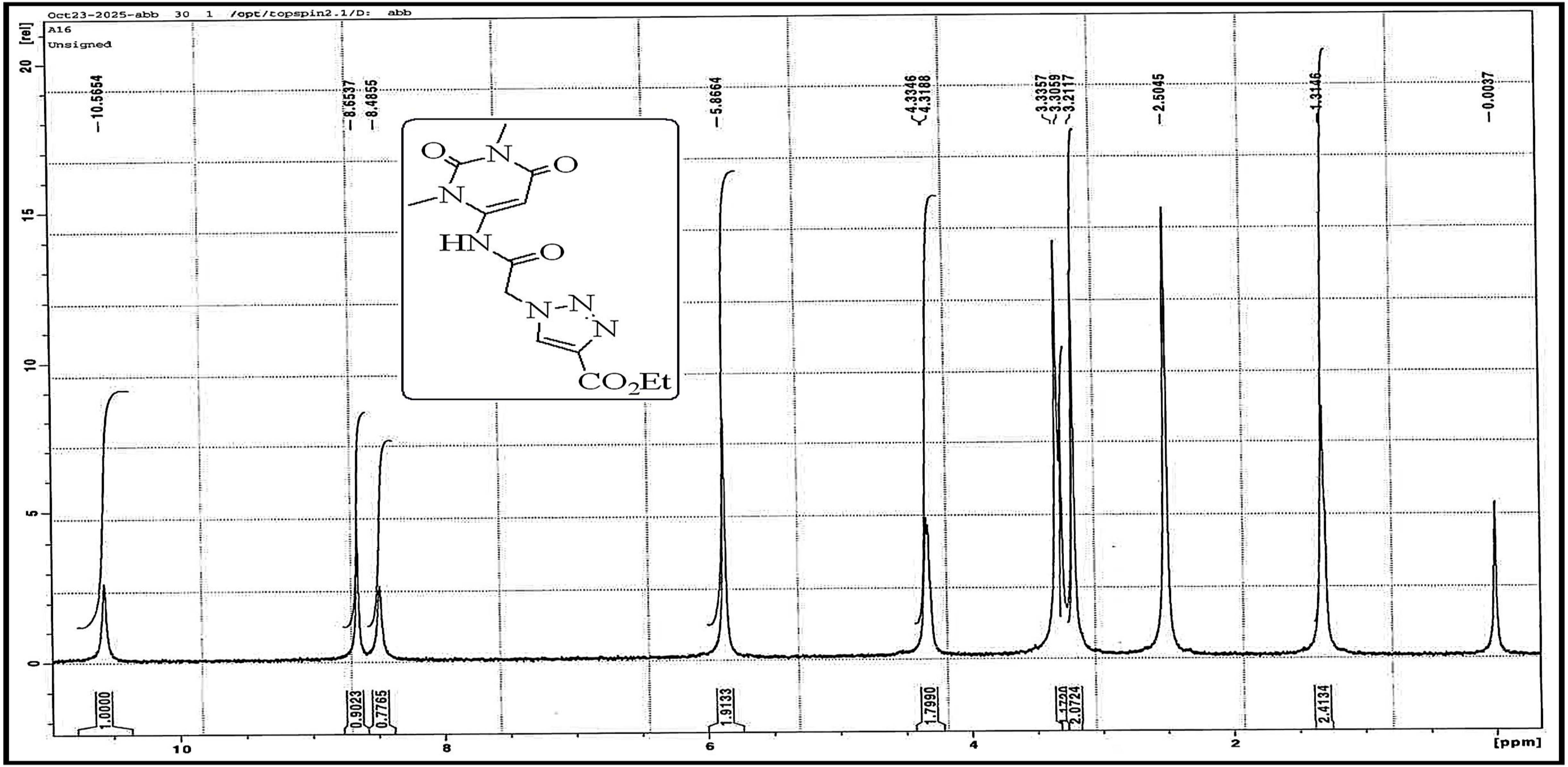

SI figure 33: Expanded ^{1}H NMR spectrum of **8c**

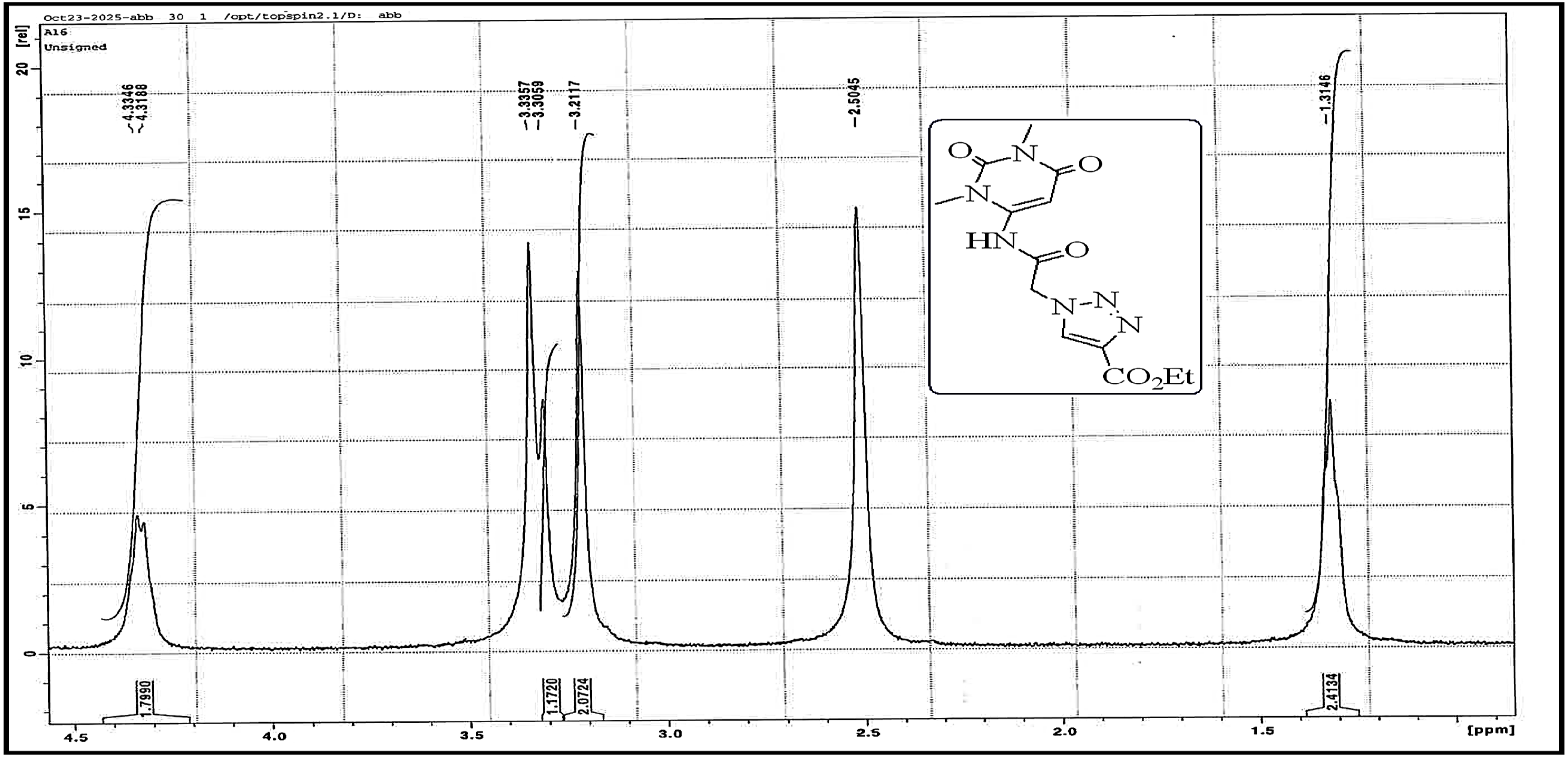

SI figure 33: Expanded ^{1}H NMR spectrum of **8c**

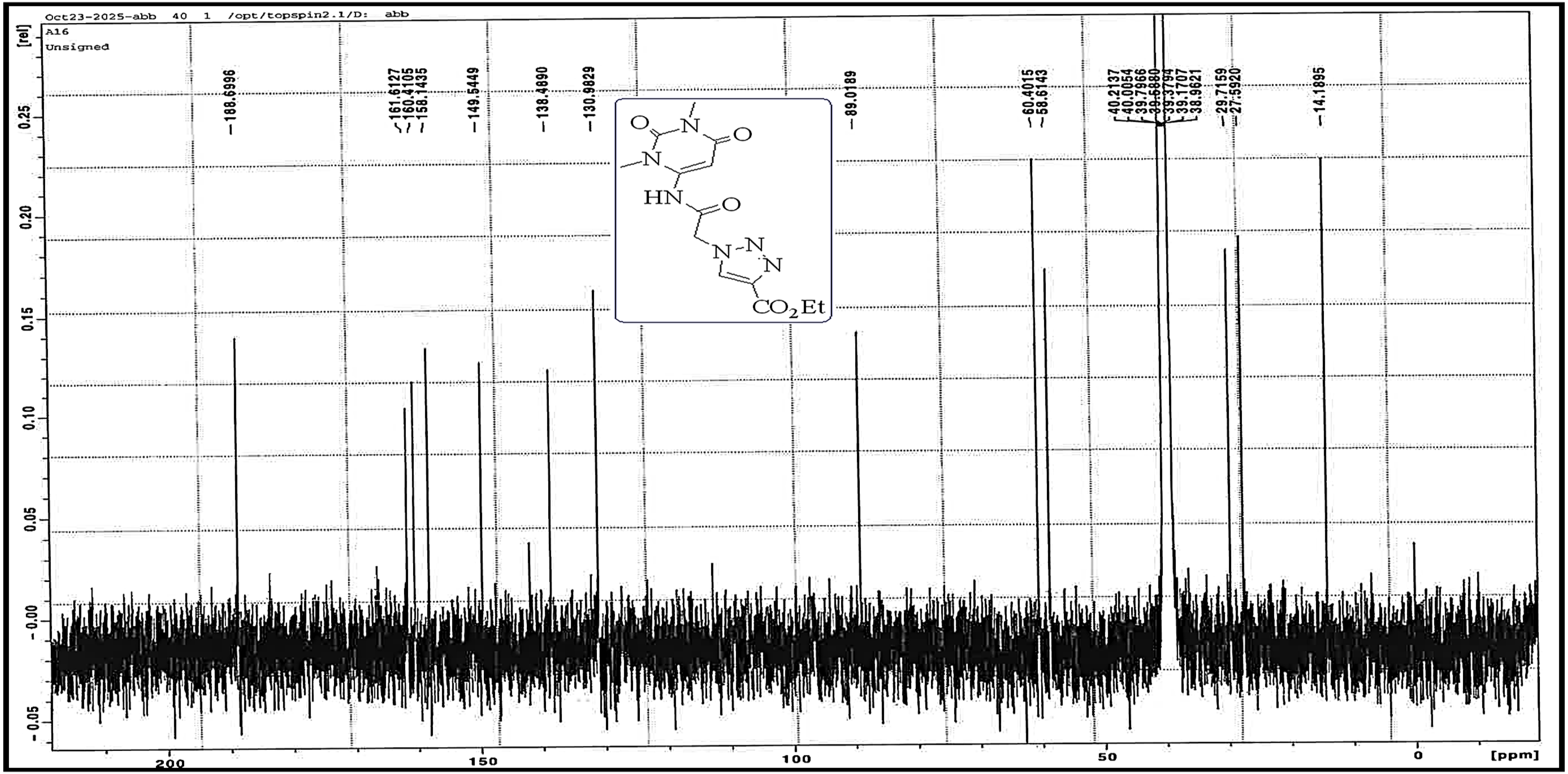

SI figure 35: ^{13}C NMR spectrum of **8c**

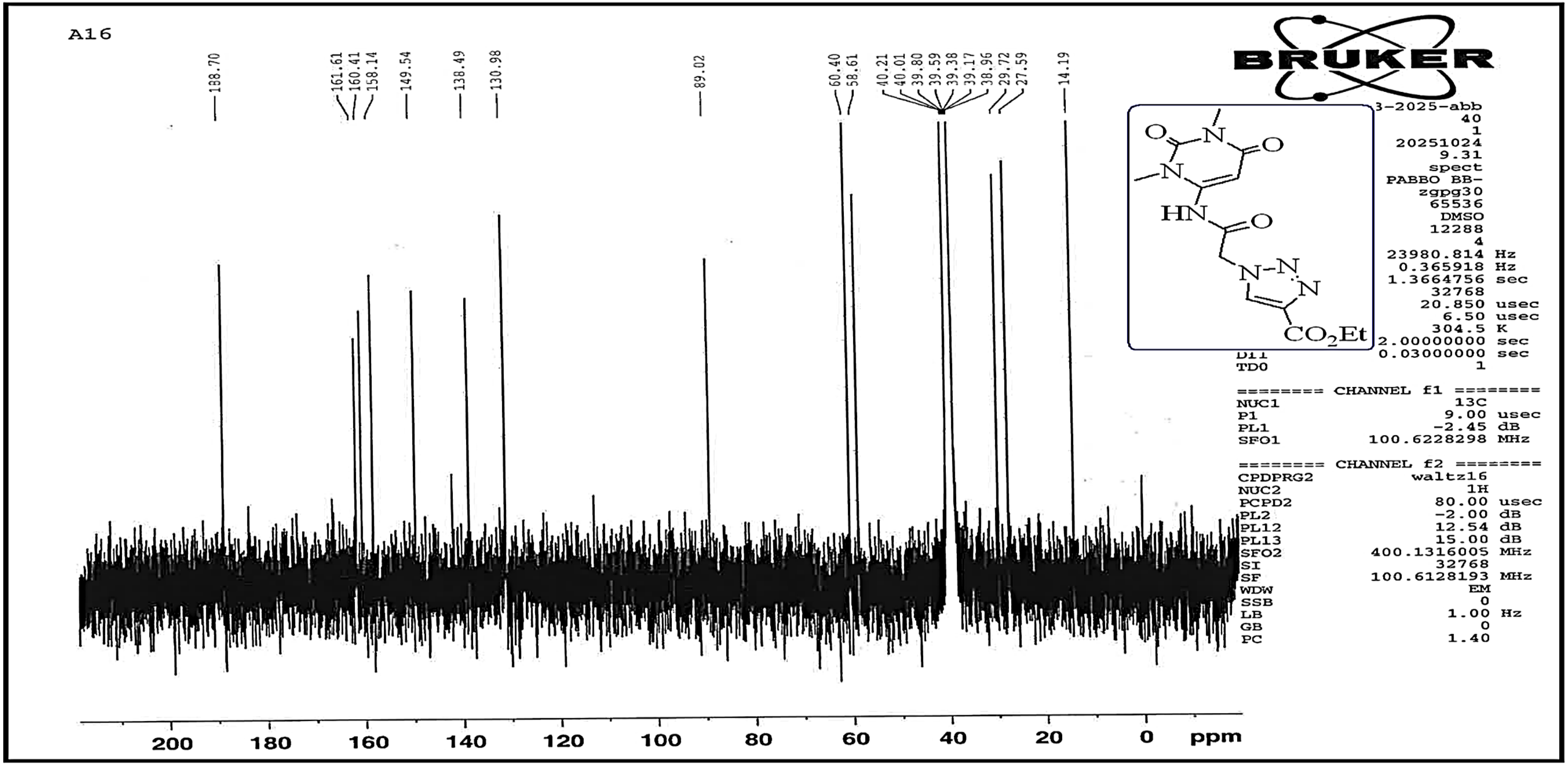

SI figure 36: ^{13}C NMR spectrum of **8c**

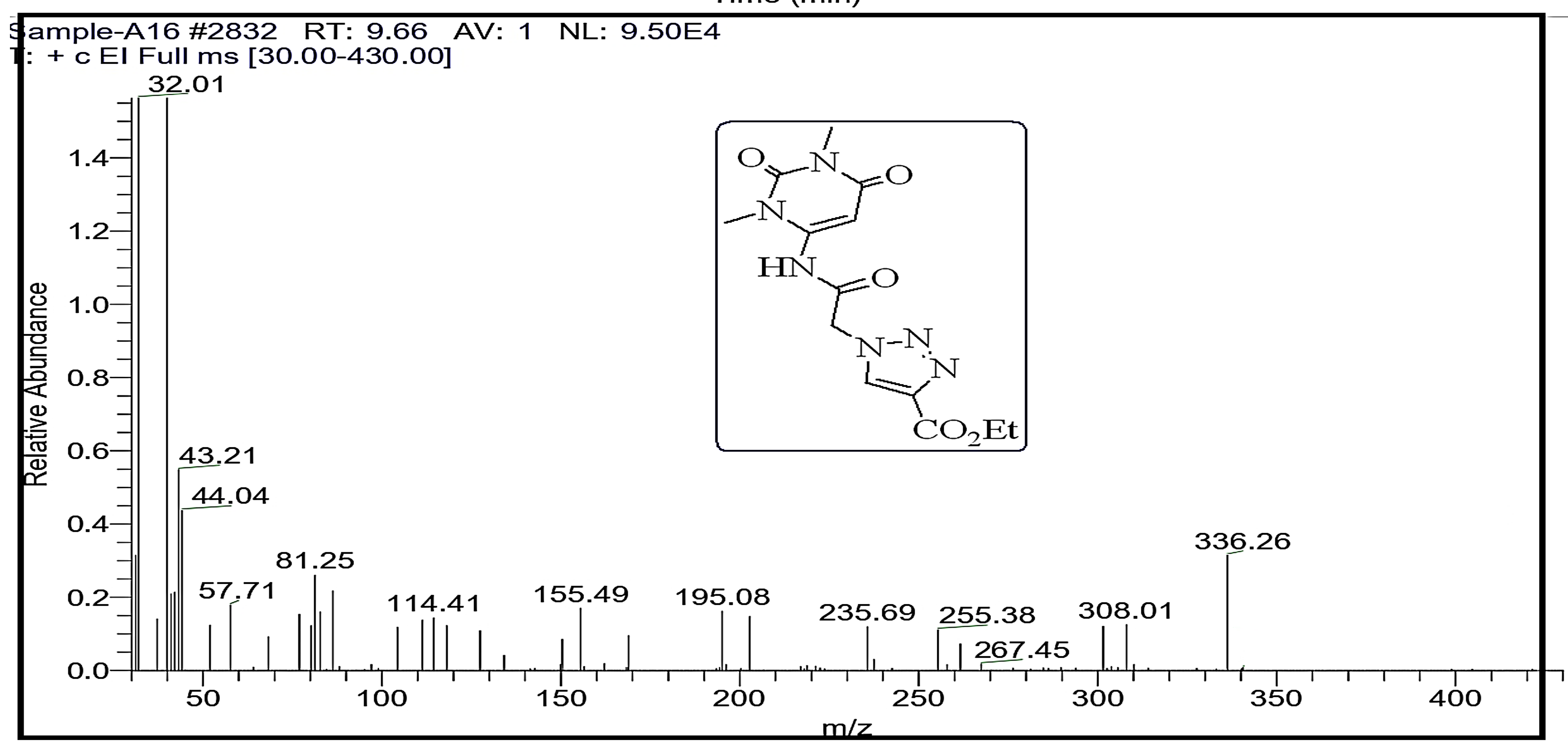

SI figure 37: Mass spectrum of **8c**

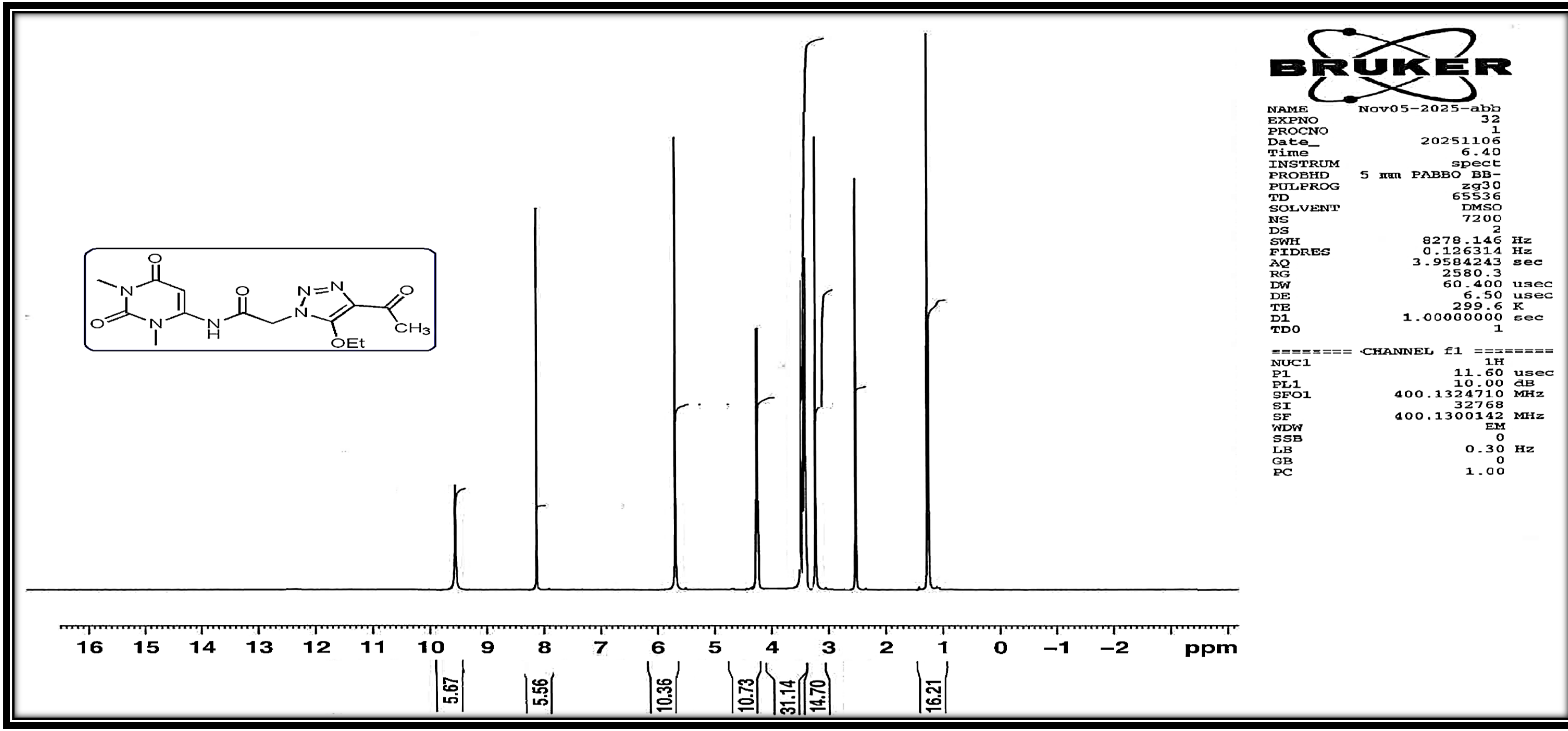

SI figure 38: ^{1}H NMR spectrum of **10b**

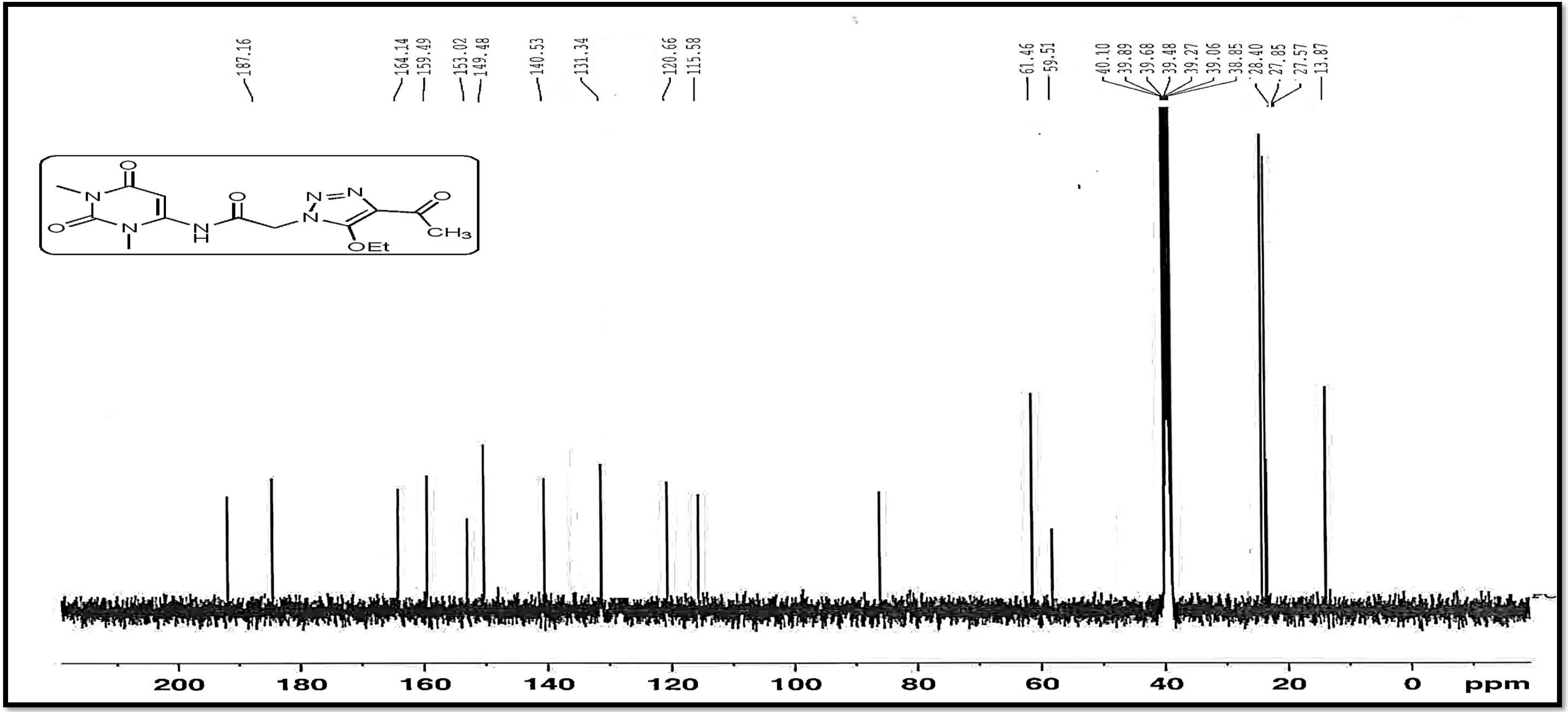

SI figure 39: ^{13}C NMR spectrum of **10b**

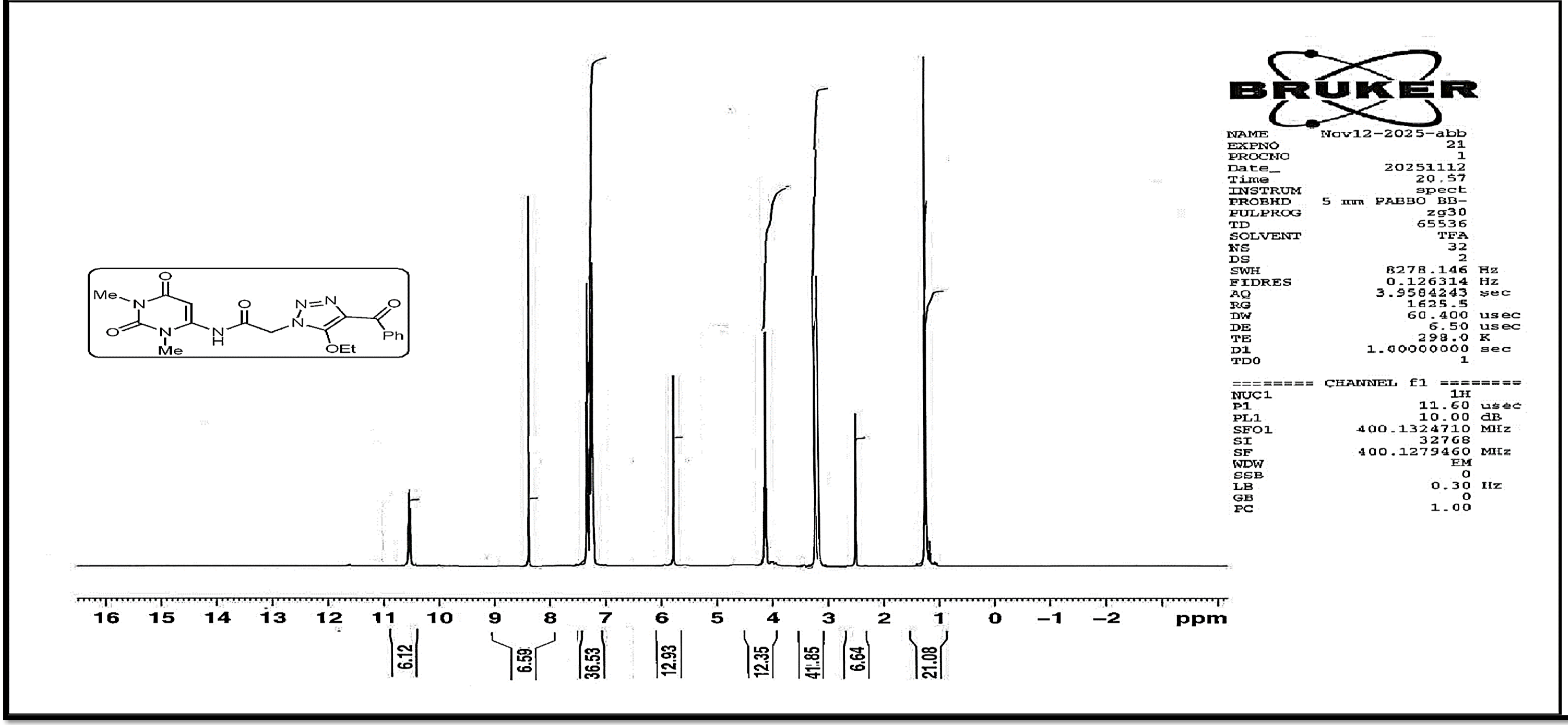

SI figure 40: ^{1}H NMR spectrum of **10c**

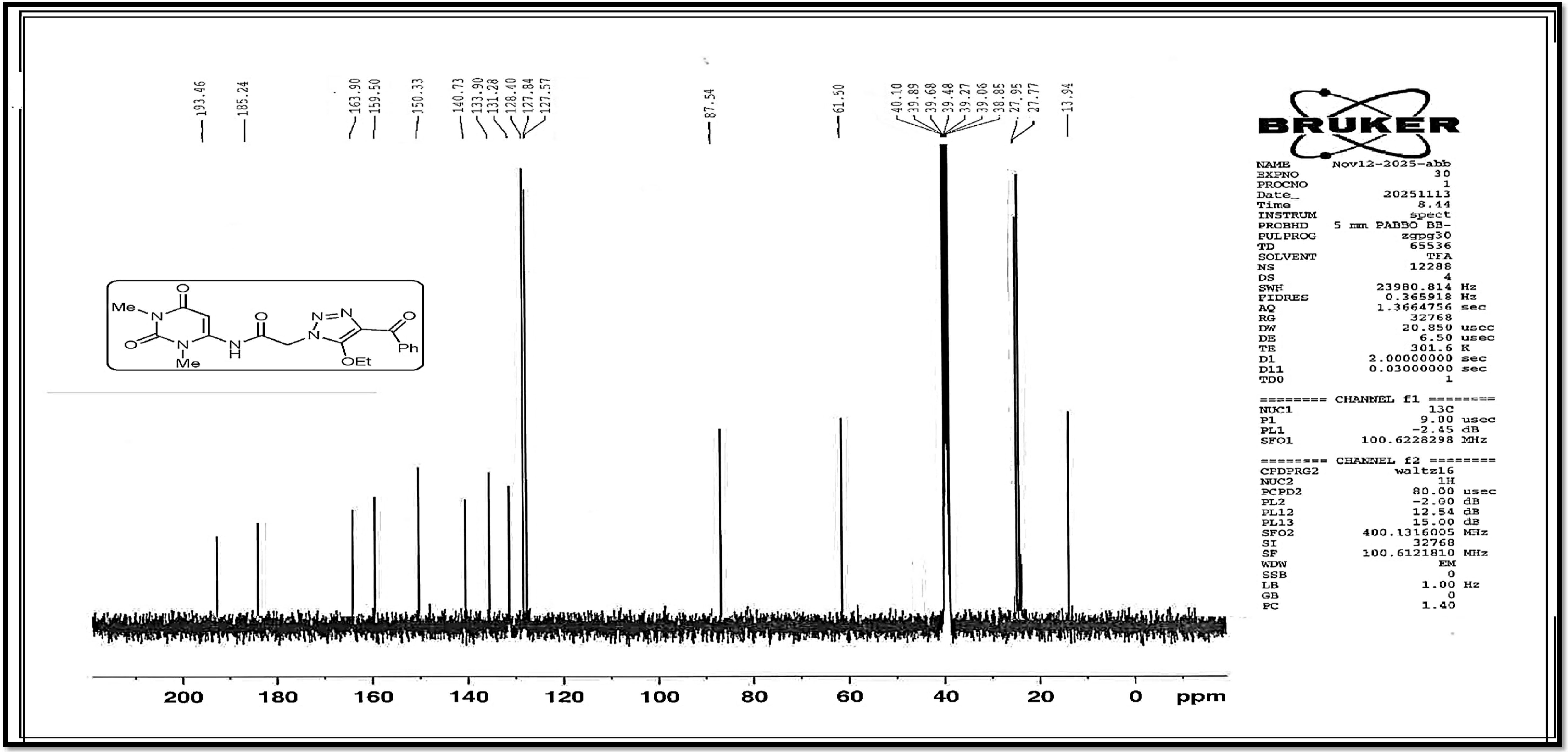

SI figure 41: ^{13}C NMR spectrum of **10c**

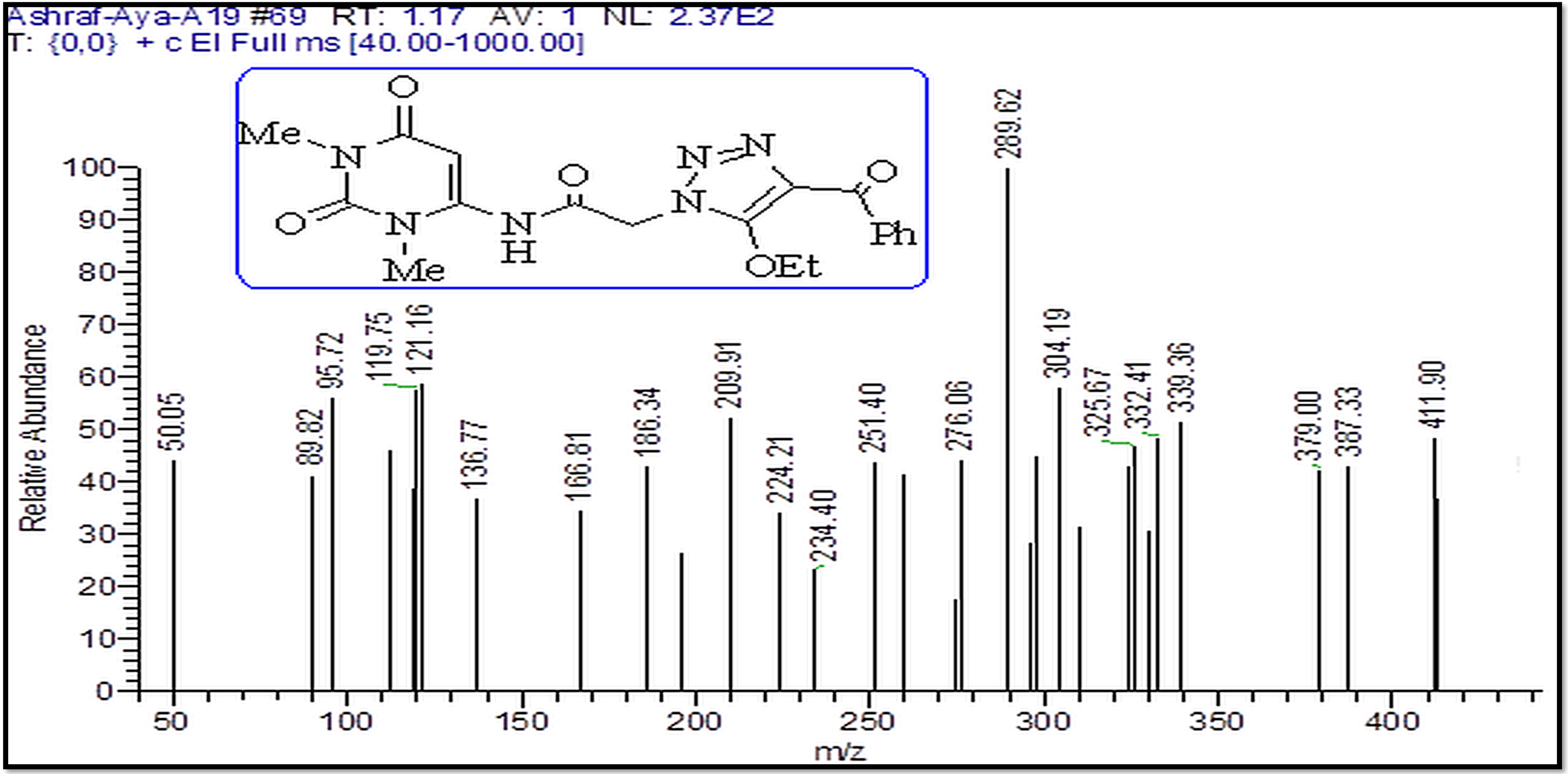

SI figure 42: Mass spectrum of **10c**
